# A study of human resource competencies required to implement community rehabilitation in less resourced settings

**DOI:** 10.1186/s12960-017-0240-1

**Published:** 2017-09-22

**Authors:** Brynne Gilmore, Malcolm MacLachlan, Joanne McVeigh, Chiedza McClean, Stuart Carr, Antony Duttine, Hasheem Mannan, Eilish McAuliffe, Gubela Mji, Arne H. Eide, Karl-Gerhard Hem, Neeru Gupta

**Affiliations:** 1 Centre for Global Health, Trinity College Dublin, 7-9 Leinster Street South, Dublin 2, Ireland; 2 Department of Psychology, Maynooth University, John Hume Building, Maynooth University North Campus, Co. Kildare, Ireland; 3 Faculty of Medicine and Health Sciences, Centre for Rehabilitation Studies, Stellenbosch University, P.O. Box 241, Cape Town, 8000 South Africa; 4 Olomouc University Social Health Institute, Palacký University, Univerzitní 22, 771 11 Olomouc, Czech Republic; 5 School of Psychology, Massey University, Private Bag 102-904, North Shore, Auckland, 0745 New Zealand; 6 International Centre for Evidence in Disability, London School of Hygiene and Tropical Medicine, Keppel Street, London, WC1E 7HT England; 7 School of Nursing, Midwifery, and Health Systems, UCD Health Sciences Centre, University College Dublin, Belfield Dublin 4, Ireland; 8 Department of Health Research, SINTEF Technology and Society, P.O. Box 124 Blindern, NO-0314 Oslo, Norway; 9 Faculty of Medicine and Health Sciences, Department of Neuromedicine and Movement Science, Norwegian University of Science and Technology, N-7491 Trondheim, Norway; 10 Department of Sociology, University of New Brunswick, P.O. Box 4400, Fredericton, New Brunswick E3B 5A3 Canada

**Keywords:** Human resources, Delphi study, Rehabilitation, Realist synthesis, Community-based rehabilitation, Low resource settings

## Abstract

**Background:**

It is estimated that over one billion persons worldwide have some form of disability. However, there is lack of knowledge and prioritisation of how to serve the needs and provide opportunities for people with disabilities. The community-based rehabilitation (CBR) guidelines, with sufficient and sustained support, can assist in providing access to rehabilitation services, especially in less resourced settings with low resources for rehabilitation. In line with strengthening the implementation of the health-related CBR guidelines, this study aimed to determine what workforce characteristics at the community level enable quality rehabilitation services, with a focus primarily on less resourced settings.

**Methodology:**

This was a two-phase review study using (1) a relevant literature review informed by realist synthesis methodology and (2) Delphi survey of the opinions of relevant stakeholders regarding the findings of the review. It focused on individuals (health professionals, lay health workers, community rehabilitation workers) providing services for persons with disabilities in less resourced settings.

**Results:**

Thirty-three articles were included in this review. Three Delphi iterations with 19 participants were completed. Taken together, these produced 33 recommendations for developing health-related rehabilitation services. Several general principles for configuring the community rehabilitation workforce emerged: community-based initiatives can allow services to reach more vulnerable populations; the need for supportive and structured supervision at the facility level; core skills likely include case management, social protection, monitoring and record keeping, counselling skills and mechanisms for referral; community ownership; training in CBR matrix and advocacy; a tiered/teamwork system of service delivery; and training should take a rights-based approach, include practical components, and involve persons with disabilities in the delivery and planning.

**Conclusion:**

This research can contribute to implementing the WHO guidelines on the interaction between the health sector and CBR, particularly in the context of the Framework for Action for Strengthening Health Systems, in which human resources is one of six components. Realist syntheses can provide policy makers with detailed and practical information regarding complex health interventions, which may be valuable when planning and implementing programmes.

**Electronic supplementary material:**

The online version of this article (10.1186/s12960-017-0240-1) contains supplementary material, which is available to authorized users.

## Background

It is estimated that over one billion persons worldwide have some form of disability, as defined by the United Nations Convention on the Rights of Persons with Disabilities (UNCRPD) as ‘those who have long-term physical, mental, intellectual, or sensory impairments which in interaction with various barriers may hinder their full and effective participation in society on an equal basis with others’ [1]. However, the World Report on Disability highlighted a lack of knowledge and prioritisation of how to serve the needs and provide opportunities for people with disabilities [2]. The UNCRPD [1], the CBR guidelines [3], and the WHO Global Disability Action Plan all represent important contributions at policy level to realising the rights of people with disabilities. However, persons with disabilities worldwide still face a myriad of barriers to high-quality and sustained service provision and access, and as noted by the World Report one of the critical components of this is provision of the skilled human resources required to implement the health-related aspects of the CBR guidelines.

Within the domain of health, there is a global deficit of over four million trained health workers with low-income countries largely affected [4, 5]; however, this is not specifically for the provision of services for people with disabilities [6]. A study including 54 low- and middle-income countries has indicated that an additional 239 000 full time staff are required to address the burden of mental health alone, with 59% of the middle-income countries and all of the low-income countries having a shortage of mental health workers [7].

The World Report on Disability notes ‘global information about the rehabilitation workforce is inadequate. In many countries, national planning and review of human resources for health do not refer to rehabilitation’ (p. 108) [2]. Where salient reviews of CBR programmes do exist, for instance in Malawi [8], Kenya, Tanzania and Uganda [9], these indicate that shortages of human resources and knowledge is a major constraint in implementation. A recent systematic review of the effectiveness of alternative cadres in CBR [10] highlighted the need for systematic research on the training, performance, development and impact of the workforce engaged in rehabilitation activities in addition to a coordinated global response [11]. A more recent study in Madagascar highlighted barriers to implementing the WHO’s Disability Action Plan as a lack of human resources and disconnect between acute and community services [12].

Rehabilitation work being undertaken by ‘lower level’ cadres has historically evolved from professions’ disciplinary domains, rather than being based on a set of tasks that need to be conducted in an integrated fashion. Scientific approaches to job specification, including evidence-based empirical approaches to articulating the core competencies to perform specific tasks, or a family of tasks, now exist. By specifying the core competencies required to do a job successfully and effectively, service systems are able to select, train, and appraise personnel more effectively [13].

The paucity of research on the rehabilitation workforce is likely to constrain our ability to follow-through and deliver on the initiatives described above. Shortages of appropriately trained and deployed human resources for rehabilitation constitute a serious strategic bottleneck for the development of institution and community-based services, despite multiple proposals seeking to improve such services [11, 14–17]. There is much that can be learned from other areas of human resources for health including, for instance, health workforce planning [18], inequalities [19], health workforce training, motivation, supervision and retention [20, 21], mid-level cadres in maternal health [22, 23], and in primary care [24].

Examining the current literature on the CBR workforce to gain the most comprehensive picture, and supplementing with evidence from other community health workforce areas, could make an important contribution to developing this field. With this in mind, our study aimed to answer the question of ‘what are the human resource competencies required to implement the CBR guidelines in less resourced settings?’

### Rationale for study

Focusing on human resources at the community level is advocated as a way of expanding and decentralising service delivery while increasing the supply of or access to human resources for rehabilitation [25]. Investing in communities by having an immediate and large increase in human resources at this level, including training of mid-level health workers, is recommended and advocated to address skill imbalances and for the scaling up of education and training of the health workforce [6, 26–29]. In line with strengthening the implementation of the health-related CBR guidelines, our objective was to establish the workforce characteristics at the community level that enable quality rehabilitation services, with a focus primarily on less resourced settings, where the human resource shortfalls are greatest. As CBR is considered a complex health intervention, we believe studies should incorporate complexity for a more contextually informed understanding. Theory-driven reviews are capable of providing this insight, as previous studies on rehabilitation governance [30], human resource management [31], workforce interventions for support workers [32] and the community health workforce [33] have all noted the influence such methodologies can have on understanding complex health interventions.

## Methods

**Fig. 1:**
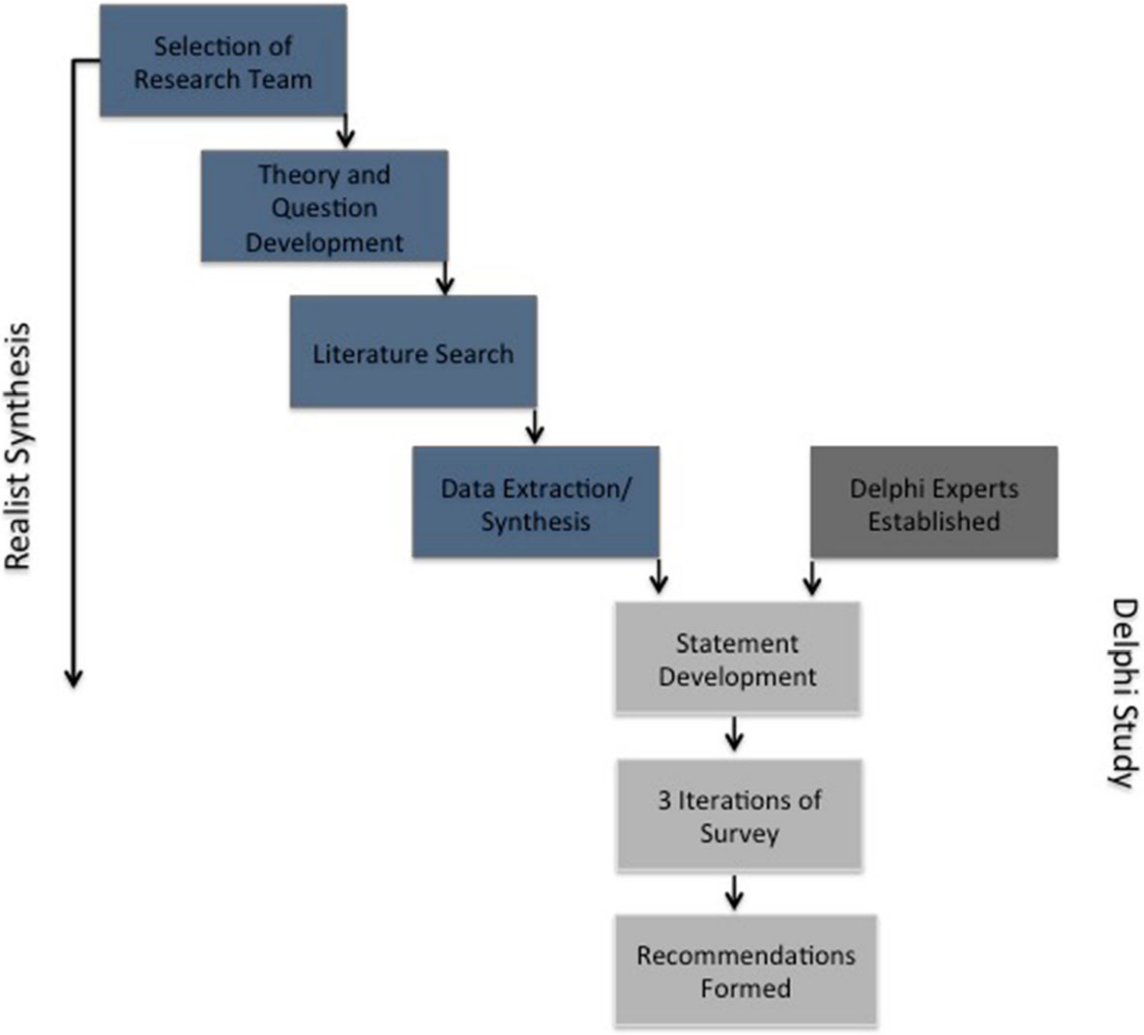
Study design overview

This study utilised two methods: a review of the relevant literature informed by realist synthesis methodology, and a Delphi survey of the opinions of relevant stakeholders regarding the findings of the review. This approach was utilised to combine the rigour and explanatory mechanisms of a realist synthesis with additional stakeholder input for greater contextualised findings. Figure 1 provides an overview of the study methods.

### Realist synthesis

Realist synthesis methodology, pioneered by Pawson [34], aims to provide an explanatory analysis for how and why social interventions work [35]. These theory-driven reviews seek to understand contextual factors and the process mechanisms through which outcomes occur. To do so, they collate a wide range of quantitative, qualitative and mixed-methods literature specifically aimed at complex interventions, and develop working theories of ‘how things work’ through investigating how contexts trigger casual mechanisms and these relationships’ associated outcomes (C + M = O) [36–38]. These relationships are expressed as context-mechanism-outcome configurations (CMOCs) [39]. Realist syntheses allow researchers to address issues beyond effectiveness and to answer questions by drawing on literature that explains why, for whom, and under what circumstances programmes work. They are more explanatory than judgmental and are suitable for reviews of complex social phenomena that involve human decisions and actions and desire to understand the context and complexity of interventions [33, 34, 38]. As such, they can provide policy makers with detailed and practical information regarding complex health interventions, which may be valuable when planning and implementing programmes and provide recommendations that address the facilitators and barriers to service delivery [40, 41].

For the purpose of this review, we refer to characteristics as a description of a person's or jobs’ attributes; skills as a set of learned abilities, often through training programmes; and competencies as a combination of skills and behaviours that are required to perform a job to a satisfactory degree.

#### Realist synthesis adaptation

This research has been adapted from realist synthesis as described by Pawson and Tilley for two main reasons. First, and frankly, we recognise that we entered into this methodology with an incomplete understanding of its details. The research objectives and proposed questions and methodology were provided to the team. As our learnings on realist studies advanced, we had to adapt both our objectives and methods to find a balance between meeting the commissioned requests and needs, and attempting to maintain methodological rigour. Second, the research outputs required programmatic recommendations that were easily digestible to implementers, demanding that our study be framed within a very pragmatic lens, likely at the cost of more robust theoretical influence. Main adaptations were: (a) the initial theory was developed mainly through team consultation with minimal literature input to ensure consistency with research output requirements; (b) sub-questions were used to organise our data and were prepared prior to searching to best contribute to the research objective; and (c) the addition of the Delphi process, which aided in contextualising review findings while also translating these into implementation-focused recommendations.

#### Scoping—question and initial theory

The initial review question sought to identify best practice for the development of the rehabilitation workforce for health-related rehabilitation for health systems strengthening. During the initial theory development and scoping process, this question was refined to better reflect the initial theory to: ‘what rehabilitation workforce characteristics enable quality rehabilitation services at the community level in less resourced settings and how, why and for whom do these work best?’

The initial programme theory to be explored was developed through an on-going iterative process that involved consultations with research team members and the investigation of some relevant literature [42]. The overall initial theory was phrased as ‘to achieve equitable and quality rehabilitation services for persons with disabilities in less resourced settings, the workforce should be available and/or strengthened at the community level’, this being developed through and supported by salient literature [6, 27, 29]. We considered there to be several benefits in this approach. Notably, it considers the lack of knowledge or clear definition of what is a rehabilitation worker [43], and the severe shortage of such workers in less resourced settings [14]. It also considers more vulnerable populations of people requiring health-related rehabilitation; less resourced settings with the fewest human resources; issues of decentralisation and equity of access to services; issues of community involvement and ownership of rehabilitation programmes; and making the workforce more disability-sensitive [25, 44]. Six ‘framework themes’ were pre-identified, which were aligned to the sub-questions, relevant to the workforce, and thought to best contribute to and organise arising data (Table 2).

#### Search process

The search consisted of extensive snowballing and a systematic style approach. Search terminology was developed in consultation with team members and a search librarian (Additional file 1). Ten bibliographic databases were searched: PubMed, WHOLIS, Embase, Scopus, CIRRIE, REHABDATA, LILACS, PsycINFO, Scie, and AIM (African Index Medicus). The archives of the journal Disability, CBR & Inclusive Development were also searched. Snowballing consisted of searching references of relevant reviews and all included texts, contacting team members and other stakeholders requesting documents, emailing other individuals and 13 organisations working in disability and rehabilitation, and conducting searches on search engines. One author (BG) conducted the searches. EPPI-Reviewer 4, a systematic review management software programme, was used to assist in document management including the identification of duplicates.

#### Selection and appraisal of documents

Selection of articles for inclusion in the realist synthesis occurred in stages and was performed by two reviewers. Articles returned from the database search were subject to title review and, if suitable, subsequent abstract and full text review by two researchers independently. At each stage, screening was conducted by two reviewers independently: title (BG and JMV), abstract (BG and MML) and full-text (BG and HM). Discrepancies were mediated by a third reviewer until consensus was reached. All articles from snowballing were subject to full text review.

Articles were deemed ‘suitable’ based on their potential ability to contribute to the theory revision. The selection, therefore, was not accompanied by discrete inclusion and exclusion criteria, but based on the articles’ content and researchers’ judgement relating to applicability. As such, the selection criteria were quite open, with the only restrictions relating to publication year (2003 and later); articles including a rehabilitation component with reference or implications to the workforce; and rehabilitation services that were specific and/or relatable to health rehabilitation.

#### Data extraction

Using the data extraction table, information was collected on the intervention, the context and potential explanatory mechanisms in addition to article descriptions, which allowed reviewers to extract context-mechanism-outcome configurations (CMOCs) from each article. Two reviewers (BG and SC) independently reviewed all included articles and for each filled in as much of the data extraction sheet as possible, including CMOC development.

#### Analysis and synthesis process

The primary reviewer synthesised the findings from both reviewers’ CMOC extraction of articles and used a data analysis matrix, which was adapted from a previous realist synthesis [45] to include more details on the workforce. This consisted of extracting characteristics on study design, intervention (setting, population), workforce (cadre, role description, training, supervision) and reported contextual factors, mechanisms, outcomes and potential CMOCs for the interventions.

The CMOCs from both reviewers were populated into the six framework themes proposed at the beg﻿inning stages of the project (Table 2), similar to an evaluative framework [46]. In instances where CMOCs from the two reviewers were contrasting and/or widely different, we re-reviewed data extraction tables and discussed the reviewers’ formulations rationale. However, no CMOC adjustments were made at this stage. We then synthesised any similar CMOCs, documenting the evidence source(s). CMOCs that occurred across various evidence sources were modified into statements conducive to the accompanying Delphi survey. The results of these phases were then iterated back into the initial programme theory for further refinement.

### Delphi survey

The Delphi survey is a consensus finding tool commonly used in health and social science research [47], which maintains anonymity and confidentiality, has multiple iterations and controlled feedback, and allows arithmetic aggregation of group scores [48, 49]. This group facilitation technique is designed to transform individual opinion into consensus by aiding decision making based on the opinions of experts [47, 50]. It has been credited with reducing respondent bias and increasing clarity of opinions [51, 52], while allowing for a geographically dispersed group through the use of an online survey tool [48]. For the purpose of this review, the Delphi was undertaken to enhance trustworthiness and further refine the findings.

CM lead the Delphi study, with support from MML and BG. A panel of experts who could provide insight into the workforce for health-related rehabilitation was recruited through purposeful sampling via email, conforming to recommendations of 10 to 25 participants [53, 54]. Participants were asked to complete an online survey administered through SurveyMonkey. Participants indicated their level of agreement with a particular statement by rating it on a Likert scale ranging from 1 to 5 (Strongly Disagree to Strongly Agree). A statement was deemed to have high levels of agreement if it achieved an average rating of 4 or above and a standard deviation of less than 1, based on a previous health science study [55]. Statements not having high levels of agreement were modified based on participant feedback and subjected to subsequent rounds. Participants were also asked to provide written opinions on a number of statements, which were used to make further adjustments for the next iteration.

## Results

### Realist synthesis

**Fig. 2:**
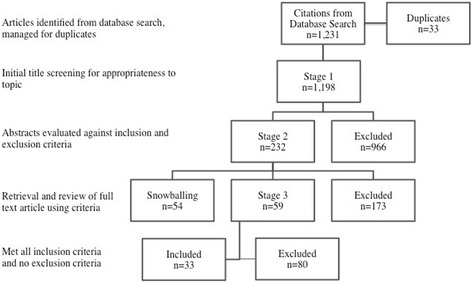
Document flow diagram

Searching was conducted between October 2013 and February 2014. A total of 1231 articles were identified from the database searches. An additional 54 articles were identified during the snowballing process. After a multi-researcher screening process, 33 articles were identified for inclusion in this study according to the document flow diagram in Fig. 2.

#### Document characteristics

More than 30 countries were represented in the articles. Sub-Saharan Africa had the highest representation with 18 articles: Somalia [56]; Eritrea [57, 58]; Burundi [59]; Sudan [59]; Uganda [59–61]; Zambia [59]; Lesotho [62]; Tanzania [59]; Kenya [63, 64]; Democratic Republic of the Congo [65]; Ghana [66]; Benin [66]; and South Africa [67–69]. Sixteen articles focused on areas in South Asia: India [56, 58, 70–73]; Pakistan [56, 58, 74, 75]; Bangladesh [76]; Afghanistan [77]; Nepal [78, 79]; and Sri Lanka [80]. Fourteen articles reported on countries in East Asia and the Pacific: Mongolia [56, 58, 81]; China [82]; Hong Kong [82]; Philippines [59]; Papua New Guinea [58]; Cambodia [59]; Thailand [59]; Vietnam [56, 58, 83]; and Indonesia [56, 59]. Two countries in North Africa, Liberia [56] and Egypt [58], were included and two countries from The Middle East, Palestine [84, 85] and Iraq [59]. One paper [86] discussed information from all countries in The Pacific Islands, one [87] reported on the workforce from a global perspective, and one article [82] included information from Australia, along with China and Hong Kong as previously stated.

There were 17 articles that reported on the workforce in relation to physical rehabilitation [56, 58–63, 66–69, 73, 76, 78, 81, 83, 87]. Twelve reported on the workforce in relation to persons with mental health problems [59, 64, 65, 70–72, 74, 75, 77, 79, 80, 82]. In the remaining five included articles [57, 84–86, 88], the rehabilitation workforce either worked with persons with both physical disabilities and mental health problems, or the distinction was unclear.

The reported workforce characteristics differed in regard to cadre description, job requirements, training, supervision and ratio of worker to client or household, a summary of which is provided in Table 1.

**Table 1:** Workforce characteristics identified in articles

	Description	Source
Cadres	Lay health workers	(Mijnarends et al., 2011); (Rahman et al., 2008); (Como and Batdulam, 2012); (Rahman, 2007); (Armstrong et al., 2011); (Raja, 2012); (Lund et al., 2013); (Ayoughi et al., 2012); (Balaji et al., 2012); (Murray et al., 2011); (Chatterjee, 2003); (Claussen, 2005); (Johnson, 2004); (Llewellyn et al., 2012)
	Community-based rehabilitation workers	(Mijnarends et al., 2011); (Grut, 2004); (Magallona and Datangel, 2012); (Sharma, 2003); (Penny et al., 2007); (Eide, 2006); (Nilsson, 2005); (Deepak, 2010); (Rule, 2013); (Deepak, 2011); (Jadin, 2005); (Children, 2010); (Mendis, 2009); (Llewellyn et al., 2012)
	Mid-level rehabilitation workers	(Rule, 2013); (Chappell, 2009); (Llewellyn et al., 2012); (Dawad and Jobson, 2011); (Finkenflügel and Rule, 2008)
	Paraprofessionals	(Bass et al., 2013); (Llewellyn et al., 2012)
	Nurses	(Mijnarends et al., 2011); (Lund et al., 2013)
	Physicians	(Mijnarends et al., 2011); (Ayoughi et al., 2012); (Penny et al., 2007); (Ng et al., 2009); (Chatterjee, 2003); (Raja, 2012); (Llewellyn et al., 2012)
	Occupational/physiotherapists	(Penny et al., 2007); (Llewellyn et al., 2012); (Ng et al., 2009); (Adams et al., 2012); (Finkenflügel and Rule, 2008)
	Community groups	(Hartley, 2003); (Deepak, 2010)
	Other	(Chatterjee, 2003); (Raja, 2012); (Adams et al., 2012); (Ng et al., 2009); (Llewellyn et al., 2012)
Requirements	4 years post-primary	(Bass et al., 2013)
	Secondary school	(Rahman et al., 2008); (Murray et al., 2011); (Rahman, 2007)
	10 years of school	(Balaji et al., 2012)
	From communities	(Rahman et al., 2008); (Balaji et al., 2012); (Penny et al., 2007); (Chatterjee, 2003); (Claussen, 2005); (Jadin, 2005); (Bass et al., 2013); (Rahman, 2007); (Lund et al., 2013)
	Literate	(Lund et al., 2013)
	Min. 1 year experience	(Bass et al., 2013)
Training	6 sessions	(Adams et al., 2012)
	2–5 days	(Rahman, 2007); (Ng et al., 2009); (Armstrong et al., 2011); (Lund et al., 2013)
	1–2 weeks	(Como and Batdulam, 2012); (Ng et al., 2009); (Claussen, 2005); (Bass et al., 2013)
	6 weeks	(Claussen, 2005); (Bass et al., 2013)
	40–60 days	(Balaji et al., 2012); (Chatterjee, 2003)
	100 days	(Children, 2010)
	3.5 months	(Ayoughi et al., 2012)
	2 years	(Rule, 2013); (Ayoughi et al., 2012); (Chappell, 2009); (Dawad and Jobson, 2011)
	Refreshers indicated	(Rahman, 2007); (Grut, 2004)
Supervision	Weekly	(Magallona and Datangel, 2012)
	Monthly	(Balaji et al., 2012)
	2 months	(Grut, 2004)
	Quarterly reviews	(Balaji et al., 2012)
Ratio	1:20 HH	(Lund et al., 2013); (Deepak, 2010)
	1:100 HH	(Rahman et al., 2008)
	1:15–30	(Balaji et al., 2012); (Chatterjee, 2003); (Hartley, 2003); (Bass et al., 2013)
	1:50–70	(Nilsson, 2005)
	1:100	(Claussen, 2005)
	1:500	(Lund et al., 2013)

### Context mechanism outcome configurations and theory refinement

**Fig. 3:**
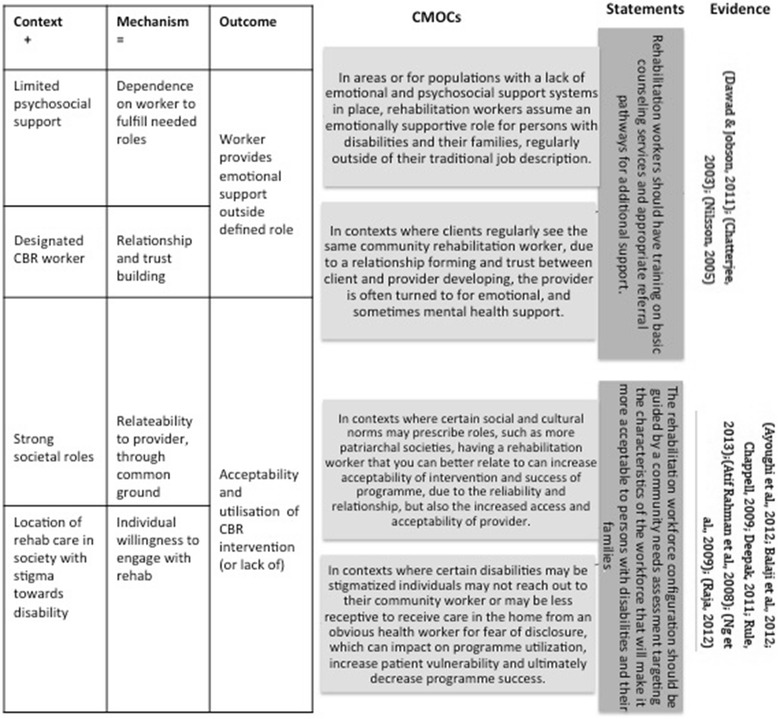
CMOC and statement development

Context-mechanism-outcome configurations were developed from the included articles using the predesigned data extraction template and analysis matrix (Additional file 2). A sample of CMOCs and their development into statements can be found in Fig. 3. A total of 29 initial statements were developed from the CMOCs, proposed as effective responses to the research questions posed in the commissioning of the research.

At this stage in the research, the CMOCs were used to refine the initial programme theory by the researchers. As a result of the insufficiently elicited initial programme theory and results spanning a very wide topic, the findings from the review continued to be organised under the six themes, with refinement occurring at this level. The synthesis of the refined statements under each theme, developed through CMOC refinement and the Delphi feedback (Table 2), resulted in the theories presented in Box 1.

**Table 2:** Rehabilitation workforce recommendations and their article evidence and Delphi consensus

Framework themes/statements	Evidence^a^	Avg.^b^	SD
1. What are the competencies needed to deliver and manage quality rehabilitation services?			
Within the delivery of rehabilitation services, there should be the designation of a specific rehabilitation coordinator/focal person who oversees the process.	[83]; [61]; [79]; [62]; [60]	4.33	0.77
Multidisciplinary supervision should be available to support the implementation of rehabilitation practices at all levels.	[71, 75]; [60]; [80]; [65]; [66]	4.17	0.86
All cadres of rehabilitation workers should receive specific training on advocacy and empowerment and be able to undertake endeavours that promote these within their communities to complement the work of disabled people's organisations (DPOs).	[56, 67, 69, 81, 87]; [57]; [61]; [78]; [74]; [79]; [64]; [62]	4	0.91
Experience and educational requirements for rehabilitation workers will be set depending on context and cadre; however, all workers, especially those at the community level, should have: strong social skills, sensitivity to others’ views and a commitment to working with persons with disabilities.	[87]; [81]	4.56	0.78
Rehabilitation services (including the additional training and supervision specific to rehabilitation), should be incorporated into all generic community health workers’ current service provision role.	[75]	*3.83*	*0.98*
Community-based rehabilitation workers should be multi-skilled and supported to take a holistic problem-based approach, with appropriate referral mechanisms to other more specialised service providers.	[78]; [56]; [62]; [72]	4.06	0.94
Skill-set mix			
In some situations, a community rehabilitation cadre should be trained with a broad range of generic rehabilitation skills (rehabilitation skills that are applicable to a large number of service users) and comprehensive knowledge on disability.	[56, 67, 69]; [63]	4.39	0.5
In some situations, a community rehabilitation cadre should be trained with specialised context specific rehabilitation skills.	[77]	4.06	0.87
In some situations, a community rehabilitation cadre should be trained with generic rehabilitation skills (rehabilitation skills that are applicable to a large number of service users) as well as one specialised area of rehabilitation.	[87]	4.06	0.42
2. Who should be trained to develop the competencies required for the delivery and management of rehabilitation services at each level of the health care system?			
Persons with disabilities (including different types of disabilities) should be encouraged and supported to train as rehabilitation workers so that the service reflects the communities they serve.	[69]; [85]	4.33	0.59
Different workforce mixes are going to be required in different contexts, and service providers should be open to a combination of: specialists, generic community rehabilitation cadres, and a cadre combining some specialist and some generic skills.	[87]; [57]	4.28	0.75
While generic community health workers should be aware of the rehabilitation needs of persons with disabilities and be able to make appropriate referrals, it is not realistic to expect them to provide these services in addition to their current service provision role.		*3.5*	*1.25*
Community-based rehabilitation workers are an effective means of identifying and targeting persons with disabilities.	[67, 77]; [72]; [78]; [60]; [87]; [76]; [74]	4.78	0.43
With appropriate training and availability of referral supports, community-based rehabilitation workers can provide services to persons with both physical and mental disabilities.	[75, 77]; [69]; [72]; [64]	4.56	0.61
3. What are the strategies which work to enable rehabilitation personnel to develop and maintain the competencies required for the delivery of rehabilitation services?			
Clear job descriptions and expectations for all rehabilitation cadres should be developed collaboratively with the workforce, managers/implementers and government bodies.	[62]; [67]; [87]	4.72	0.46
Training of the rehabilitation workforce should involve persons with disabilities (including different types of disabilities), in the planning and delivery of the training courses.	[69]; [72]; [70]	4.5	0.62
Training of rehabilitation workers should use a context sensitive, rights-based approach and encourage problem-based learning and discussions.	[62]; [56]; [87]; [82]	4.5	0.78
Supervision of the rehabilitation workforce should be supportive and involve frequent practice observation and meetings that adopt collaborative problem-solving approaches.	[71, 75, 83]; [60]; [65]	4.67	0.48
The self-efficacy of rehabilitation workers, specifically those in lower level cadres, is important for job commitment, satisfaction and subsequently retention and motivation of workers.	[75, 83]; [60]; [59]; [80]; [58]; [66]	4.28	0.57
Community rehabilitation workers require respect and recognition as professionals, which includes certification and acknowledgement of their decision-making abilities, opportunities for further training and career advancement and where feasible, should be financially compensated for their work.	[83]; [80]; [58]	4.22	0.94
The area of rehabilitation is a delicate and stressful area and requires self-awareness on the part of the health worker and requires the provision of time and spaces for consistent reflection and supportive debriefing for healthcare workers.	[85]	*4.28*	*1.02*
4. What are the strategies which work to increase the supply and improve the distribution of rehabilitation personnel required for the delivery of rehabilitation services?			
The rehabilitation workforce should be structured through an integrated tiered system, from community work to facility-based services with appropriate supervision at each level.	[75]; [80]; [61]; [72]; [79]; [85]; [86]; [64]	4.28	0.57
Community rehabilitation services can be effectively provided by shifting some rehabilitation tasks from conventionally trained rehabilitation professionals to cadres with a shorter length of training.	[68, 69, 79]; [72]; [77]; [87]; [60, 64]; [66]	4.39	0.78
Transport, compensation, and material resources should be targeted in order to provide a working environment that will be able to retain rehabilitation workers.	[62]; [60]; [81, 83]; [66]	4.5	0.62
Persons with disabilities should be involved in the selection of community-based rehabilitation workers.	[69]; [72]	*3.94*	*0.72*
5. What are the minimum requirements (i.e. ratio and competencies) of rehabilitation personnel needed for the delivery of rehabilitation services?			
Where a generic community health workforce exists, they should be trained in disability identification and awareness, rehabilitation referral, and basic service provision for persons with disabilities.	[75]; [76]; [73]; [78]; [74]; [70]; [64]	4.56	0.51
Community based workers should have a minimum generalist skill-set with specialised services being offered at the facility-based level.	[68]; [61]	4.39	0.5
All rehabilitation workers should be trained on case management, social protection, the CBR Matrix, monitoring and record-keeping.	[81]; [78, 82]; [62]; [79]; [86, 88]	4.5	0.78
All rehabilitation health workers should be trained on the CBR Matrix and the contextual challenges and practical opportunities for applying it in their area.	[56, 69]; [68]; [84]; [66]; [73]	4.44	0.7
As rehabilitation workers often emotionally support persons with disabilities and their families, they should have basic counselling skills and an understanding of appropriate referral pathways and of their limits and when to refer.	[68]; [72]; [85]	4.72	0.46
Supervisors should be equally competent in the process skills of supervision and the technical skills of rehabilitation interventions.	[87]; [71]; [59]	4.17	0.86
6. What are the characteristics of the rehabilitation workforce that facilitate equitable access to rehabilitation services?			
The rehabilitation workforce configuration should be guided by community needs assessments targeting the characteristics of the workforce that will make it more acceptable and accessible to persons with disabilities and their families.	[56, 67, 69, 71, 77]; [75]; [82]; [79]	4.5	0.62
Community-based rehabilitation services should be accountable to the communities in which they work and these communities should have mechanisms to contribute feedback regarding the services they receive.	[56, 71]; [58]; [72]; [65]; [88]	4.39	0.7

^a^List of evidence from articles is not exclusive. Several statements were not derived from the CMOCs but were suggested by our team members or developed throughout the Delphi process

^b^Average and standard deviation from the last iteration (round 3) of the Delphi Survey

Box 1 Refined Programme Theories

**Table:** 

1. The delivering of quality services by the rehabilitation workforce in less resourced settings requires multi-sectoral coordination and supportive supervision. Workers should be multi-skilled, with clinical training, advocacy and empowerment skills, and the skills to navigate and refer within the wider health system. While workers may focus on either physical or mental health rehabilitation, it is necessary that all have minimal skills in both disciplines to ensure referrals.
2. The workforce delivering rehabilitation within communities should be comprised of individuals with disabilities and community lay workers.
3. In order to maintain workforce competencies, there should be clear job descriptions, roles and responsibilities; adequate training and refresher training that takes a rights-based approach encouraging problem-based learning through a mix of theory and practice; supportive supervision with trained supervisors; an incorporation of worker self-efficacy, specifically to account for motivation and satisfaction; and appropriate support structures for workers, such as counselling services. The inclusion of persons with disabilities in the training and supervision should be a priority.
4. To improve the distribution of community rehabilitation workers in less resourced settings, an integrated tier system that places workers in communities with links to more formalised services is necessary. Task-shifting of roles to lay-workers or lower cadres is appropriate to bring services to the communities especially in resource-constricted areas. Regardless of implementation models however, workers require appropriate resources and compensation (either financial or non-financial) for job performance.
5. Minimum training requirements of a community rehabilitation worker should incorporate aspects of disability identification, referral techniques, record keeping, case management, and community advocacy and empowerment techniques. Community workers should be trained on basic counselling techniques and mental health referral mechanisms. Community rehabilitation workers should be knowledgeable on the CBR Matrix as well as social protection and the possible contextual challenges within their areas.
6. To facilitate equitable access to community rehabilitation services, the workforce should be situated within community settings, with community ownership and participation throughout the design, selection and monitoring of workforce programmes. Mechanisms for feedback for both communities and the workforce need to be integrated into programmes, with a likely CBR focal person to monitor such initiatives. A community rehabilitation worker should be preceded by, and frequently updated with, a needs assessment with involvement from persons with disabilities and communities.

### Delphi study

Nineteen participants completed a minimum of one iteration, with 18 participants completing all three rounds. The participants’ years of work ranged from 5 to 40, with an average of 21. The majority worked in several regions and both low- and high-income countries, with 13 having worked in sub-Saharan Africa, 5 in North Africa, 2 in South America, 3 in North America, 9 in Asia, 8 in South East Asia, 3 in Oceania, and 12 in Europe. Participants also reported their place of origin, with 4 being from Africa, 1 from North America, 4 from Asia, 2 from Oceania, and 7 from Europe. Listed professional experience of the participants included physiotherapy, CBR, work/development psychology, occupational therapy, functional and physical rehabilitation, rehabilitation psychology, advocacy, health and rehabilitation systems and management, research, economy and public health.

The level of agreement with the statements throughout the iterations was high, with 21 statements achieving the criteria for agreement in round 1; 23 in round 2; and 29 in round 3. Alternatively, 8 statements were not agreed upon in round 1; 9 in round 2; and 4 in round 3. Based on participant feedback, an additional 4 statements were added after round 1, and 1 in round 3. Contextual clarification and/or changes to the wording of statements were done without literature consultation. New statements or any substantial statement changes were only done if also supported by included studies.

With evidence derived from the CMOCs of the realist synthesis, and subsequently subjected to three iterations of the Delphi survey to enhance relevance, trustworthiness and provide additional contextual support, 33 statements may be considered as recommendations for developing health-related rehabilitation services through community workers, as seen in Table 2. Important to note is that these were developed from a large range of articles and stakeholder input, which represent varying contexts. Therefore, not all may be relevant to all contexts and/or programmes and readers should work with these to understand what is most useful for their endeavours. Additional file 2 provides details on the included studies’ interventions and their extracted CMOCs.

## Discussion

### Summary of findings

As noted in Table 1, there was a wide variation on the characteristics of rehabilitation workforce. This provides more justification for a realist synthesis, as these groups would be difficult to compare in a traditional systematic review. This very point required the review to look more broadly at the characteristics of workers who engage in CBR activities, not a specific cadre, in the development of the CMOCs and the subsequent recommendations. These recommendations may therefore be used to assist implementation strategies in a wider body of CBR programmes. Important to note however is that due to the varying contexts from which the articles arose, several different, often contrasting, CMOCs were found. As noted, these were not intended to be a ‘one-size-fits-all’ but were intended to help draw learning from the literature that could then be applied to achieve the best fit in different contexts.

Some general guidance for the rehabilitation workforce, arising from the study findings (Box 1) and further enhanced by additional CBR literature presented previously, may be considered across different health systems in order to inform the rehabilitation workforce’s characteristics and competencies. First, community-based initiatives can promote decentralisation and cost-effectiveness, and allow services to reach more vulnerable populations. However, motivation and retention likely need to be carefully addressed in more decentralised systems, as with other community health worker programmes. Second, the workforce providing rehabilitation in communities likely needs supportive and structured supervision by rehabilitation professionals at the facility level. Third, while the specific rehabilitation skills required may vary, there were certain standards of a community worker implementing rehabilitation common across many included studies within this research. These include aspects such as case management, social protection, monitoring and record keeping, counselling skills and mechanisms for referral. Fourth, rehabilitation workforce and service requirements that take lead from communities and work to increase community ownership may better serve individuals and work to improve the rehabilitation health system via sustainability and quality. Fifth, the often transcending roles of rehabilitation workers in the community means that workers trained on the CBR Matrix, as well as skills on advocacy and empowerment, may more holistically benefit persons with disabilities. Sixth, tiered/teamwork systems of service delivery for rehabilitation are frequently implemented and have shown important influence, with more general skills in the community and mechanisms for referrals to more specialised skills in facilities. Lastly, training of community rehabilitation workers that takes a rights-based approach, incorporates practical components, and if possible involves persons with disabilities, may be more beneficial to clients and communities. A very prominent finding throughout this work was that it is difficult to consider ‘health-related CBR components’ in isolation. Understanding and supporting the workforce requires the integration of all CBR components: health, social, education, livelihoods and empowerment.

The distinction between physical and mental health rehabilitation workers is also of interest. This may become even more relevant in the future with the predicted increase in community-based services for mental health problems [89]. It is important that governments and agencies develop plans for how to integrate the contributions of both workforce types to avoid implementing parallel services. Though in some contexts it may not be suitable to have rehabilitation workers that provide both mental health and physical rehabilitation services, it is important that these cadres are knowledgeable on each other’s activities and collaborate to identify and refer individuals as needed.

### Comparison with existing literature

Other less-resourced community-based health workforce literature has recognised the need for context-specific investigations using theory-driven methodologies [33, 90–92]. In their 2012 systematic review on CBR alternative cadres, Mannan and colleagues highlighted a dearth of studies in this area, with existing resources having contextually specific programmes limiting their ability to synthesise findings [10]. To this end, there have been several calls for more innovative CBR research methodologies [93, 94]. Similar findings across this study and Mannan’s are evident however, specifically the importance of workforce motivation; cultural structures; training on ‘soft-skills’ such as advocacy and development; and CBR workers as support workers.

### Strengths, limitations and future research directions

The use of realist approaches to study the rehabilitation workforce has provided insight and evidence on this under-researched field that would not likely be possible with other empirical approaches. The combined use of the realist synthesis and Delphi study provided a unique approach to synthesis. Our approach drew on the strength of perspectives offered by different stakeholders by allowing them to comment on possible human resource implementation processes that had an established evidence base, while at the same time allowing stakeholders to make their own suggestions.

The wide scope of the research topic required that we progressively narrow our overarching research question and our search of the literature, in order to provide a meaningful and focused analysis. However, several of the papers included in the review lacked detail on the rehabilitation workforce and this limited what could be learnt from the research studies. The Delphi method also has a number of specific limitations. For example, the panel of experts was chosen by the researchers and was dependent on their own networks and the willingness of people to participate. It also had an under-representation of persons with disabilities or service users. In particular, the realist informed review of our study has several limitations: its level of abstraction is at times very abstract due to its synthesis across differing programmes and therefore suffers from a lack of specific revised theory; time restrictions prohibited further rounds of literature searching beyond searching for clarification; we did not do a comprehensive pilot of our search; and CMOCs taken forward to the Delphi process were chosen mainly on frequency found across evidence sources, which may have excluded some valuable explanatory resources.

This review highlighted that while there are indeed programmes for rehabilitation in communities, little evidence is provided on the workforces that implement these services. Researchers and programme managers should collect and disseminate more detailed evidence on the workforce so that this body of literature can be expanded and others can learn from their experiences. It also notes a dearth of evidence from the perspective of the rehabilitation workforce. As the longer-term effectiveness of programmes depends on the retention of their workers, such investigations should be given priority in order to highlight areas of concern and provide more recommendations on how to reduce attrition and provide quality and continuity of services for persons with disabilities in communities. Studies that investigate task shifting of rehabilitation services should be conducted to provide evidence on what types of workforce configurations work best in what contexts. Further theory-driven studies on individual CBR interventions, such as realist evaluation, could help to further refine CMOCs at more specific levels of abstraction, such as individual characteristics within specific cadres, as opposed to the more programmatic abstraction level offered within.

## Conclusions

To our knowledge, there have been no realist studies conducted on the rehabilitation workforce to date. While it is clear that no ‘one size fits all’, our synthesis suggests some common features that are likely facilitative to strengthening the role of community workers in implementing community rehabilitation. Such findings can be useful to support programme design to ensure contextually specific and holistically natured programmes that focus on overall rehabilitation health worker competencies and the systems in which they operate. Contextual variations within this study were mostly attributed to the configuration of the rehabilitation system and the characteristics of the rehabilitation worker. More specific recommendations for these varying contexts can be found within, such as the need for appropriate training, supervision and motivation considerations within a tiered system, and the need for advocacy and empowerment skills when task-shifting to communities, respectively.

Our findings are consistent with other workforce studies in less resourced settings in their recognition that workforce characteristics and their management should be contextualised [95, 96]. However, this study is unique in its development of recommendations regarding how this should be done. These recommendations may be used as a support resource for community rehabilitation decision makers when designing and implementing programmes.

## Additional files

Additional file 1: Finalised search terms. (DOCX 122 kb)

Additional file 2: CBR Matrix and perceived training needs of CBR workers: a multi-country study. (DOCX 274 kb)

## Abbreviations

CBR: Community-based rehabilitation
CMOCs: Context-mechanism-outcome configurations
UNCRPD: United Nations Convention on the Rights of Persons with Disabilities
