# Supplementary material for: A study of human resource competencies required to implement community rehabilitation in less resourced settings
Source: Hum Resour Health. 2017 Sep 22;15:70. doi: 10.1186/s12960-017-0240-1 (PMC5610467; doi:10.1186/s12960-017-0240-1)
Supplement: Supplementary file 1 — Finalised search terms. (DOCX 122 kb) [file 12960_2017_240_MOESM1_ESM.docx]

# Finalized Search Terms

| 1. Workforce OR “Human Resource” OR “Health Personnel” OR “Health care provider” OR “healthcare provider” OR (‘Health’ adj2 (allied OR “aide volunteer” OR personnel OR helper)) OR “Health extension worker” OR “community health worker” OR (‘Rehabilitation’ adj (worker, facilitator)) OR “Health professional” (‘Therapist’ adj (physical OR occupational OR speech OR respiratory OR recreational OR cognitive OR social)) Or Psychiatrist OR Psychologist OR “community disability worker” OR “community rehabilitation worker” OR ("health worker" OR "health workers" OR "health working") adj (community OR alternative OR lay OR village OR lady OR peripheral OR low level OR mid level OR tertiary OR mental OR “non professional” OR professional OR family OR allied OR volunteer OR aide) OR Cadre adj (alternative OR lay OR village OR lady OR peripheral OR “low level” OR “mid level” OR “non professional” OR family OR aide agent, provider, practitioner, personnel, community, assistant) OR Provider adj (community OR alternative OR lay OR village OR lady OR peripheral OR “low level” OR “mid level” OR “non professional” OR professional OR family OR allied) OR Practitioner adj (community OR alternative OR lay OR village OR lady OR peripheral OR “low level” OR “mid level” OR “non professional” OR professional OR family OR allied) OR Assistant adj (community OR alternative OR lay OR village OR lady OR peripheral OR “low level” OR “mid level” OR “non professional” OR professional OR family OR allied)  2. “CBR” OR “Community based rehabilitation” OR “community rehabilitation” OR (rehabilitation adj (services OR support OR care OR therapy)) OR habilitation OR ((therapy OR therapies) adj (physical OR occupational OR cognitive OR complementary OR speech OR respiratory OR recreational OR social)) OR rehabilitation OR “community approaches to handicap in development” OR “CAHD” OR “community based inclusive development” OR “ILD” OR “inclusive local development” OR “participatory community development”  3. “third world” OR “less resourced” OR “less resource” OR “limited resourced” OR “limited resources” OR “LMIC” OR “LIC” OR “low income country” OR “low income countries” OR “low and middle income country” OR “low and middle income countries” OR Africa OR Caribbean OR “Central America” OR “Latin America” OR “south America” OR Asia OR “Eastern Europe” OR ((developing OR ‘less developed’ OR “least developed” Or ‘under developed’ OR poor) adj (countries or country or nation or nations or region or regions or area or areas))  1 AND 2 AND 3 |
| --- |
