# Supplementary material for: A study of human resource competencies required to implement community rehabilitation in less resourced settings
Source: Hum Resour Health. 2017 Sep 22;15:70. doi: 10.1186/s12960-017-0240-1 (PMC5610467; doi:10.1186/s12960-017-0240-1)
Supplement: Supplementary file 2 — CBR Matrix and perceived training needs of CBR workers: a multi-country study. (DOCX 274 kb) [file 12960_2017_240_MOESM2_ESM.docx]

#

| **Title:** CBR Matrix and Perceived Training Needs of CBR Workers: A Multi-country Study | | | | | | | | | | | | |
| --- | --- | --- | --- | --- | --- | --- | --- | --- | --- | --- | --- | --- |
| **Authors:** Deepak, S., Kumar, J., Ortali, F. & Pupulin E. | | | | | | | | | | | **Year:** 2011 | |
| **Summary:** Community based rehabilitation workers across 13 projects in 7 countries completed survey identifying needs and working status | | | | | | | | | | | | |
| **Setting** | **Design/Method** | | **Population** | **Intervention** | **Workforce Characteristics** | | | | | | | |
|  |  |  |  |  | **Cadres** | | | **Description** | | **Training** | **Supervision** | **Misc.** |
| Guyana India, Karnataka India, Jharkhand India, Andhra Pradesh India, Karnataka India, Karnataka Indonesia, South Sulawesi Liberia, Monrovia Gardnersville Mongolia, National Pakistan, Peshawar Somalia, Somaliland, Karnataka India, | Cross-sectional | | 103 Community-based rehabilitation (CBR) workers with minimum 2 years experience | 13 CBR projects across 7 countries, run by both NGOs and Ministry of Health, that all received some form of financial or technical support from Amici di Raoul Follereau (AIFO). | CBR workers | | CBR worker defined as “persons working at field level in urban or rural communities”. 84.8% of respondents had high school level of education or higher. The remained her middle school education or lower. 53% of respondents were full-time salaried CBR workers. All workers except for 1 project, reported working with all eight (as classified by WHO Manual on CBR), groups of persons with disabilities. | | | Each project would have different training and this was not reported on.  Study participants all reported that they were involved in all 5 domains of CBR Matrix. | Majority of projects (10) managed by NGOs; 2 by MoH; 1 by both NGO and MoH. | **Quality:**  3  MMAT: Quant 4 |
| **CMOCs** | | | | | | | | | | | | |
| **Context** | | **Mechanisms** | | | | **Outcomes** | | | **CMOCs** | | | |
| Mostly Indian setting managed by NGOs.  CBR workers varied in experience, gender, age, education, and if paid or not.  All projects involved had some activities in each of the 5 domains of CBR Matrix – with CBR workers multi-sectorial responsibilities.  All workers reported that they worked across eight different groups of people with disabilities.  Population split between under and over 30 years old, gender-balanced, high school education mainly; half salaried, half volunteers. | | 1. CBR workers knowledgeable about CBR Matrix, and how it aids in working with persons with disabilities.  2.CBR workers understand shortfalls in their training and knowledge and how this impacts on their clients  3. CBR workers see their job as one of advocating for their client  4. CBR workers advocate and understand the importance of families in CBR work, and how to counsel them, and involve them.  5. CBR workers recognize empowerment of persons with disabilities as a priority | | | | Identification of learning needs for CBR workers.  CBR Matrix useful framework to understand field-level activities.  Indicated both technical and people skills important requirement. | | | 1.Disability related technical skills, including programme management, across all five CBR matrix categories. Technical skills may not be specific to disability, since all workers reported that they worked across eight different groups of people with disabilities.  2. In terms of “global competencies” (i.e., non specific to disability type or circumstances), Advocacy and empowerment highly ranked.  3. Evidence-based training needs analysis (TNA), in this case mainly in the domains of home-based health care (promoting autonomy), working with young children for education, vocational training skills in livelihood, “how to do advocacy” for social domain, and organization of self-help groups for advocacy.  4. Empowerment for the CBR workers themselves, in deciding what their training needs may be | | | |

| **Title:** Cognitive behaviour therapy-based intervention by community health workers for mothers with depression and their infants in rural Pakistan: a cluster-randomized controlled trial | | | | | | | | | | | | | | | | | | | | | | | | | | | | | | | | | | | | | | | | | | | | | | | | | | | | | | | | | | | | | | | | | | | | | | | | | | | | | | | | | | | | | | | | | | | | | | | | | | | | | | | | | | | | | | | | | | | | | | | | | | | | | | | | | | | | | | | | | | | | | | | | | | | | | | | | | | | | | | | | | | | | | | | | | | | | | | | | | | | | | | | | | | | | | | | | | | | | | | | | | | | | | | | | | | | | | | | | | | | | | | | | | | | | | | | | | | | | | | | | | | | | | | | | | | | | | | | | | | | | | | | | | | | | | | | |  |
| --- | --- | --- | --- | --- | --- | --- | --- | --- | --- | --- | --- | --- | --- | --- | --- | --- | --- | --- | --- | --- | --- | --- | --- | --- | --- | --- | --- | --- | --- | --- | --- | --- | --- | --- | --- | --- | --- | --- | --- | --- | --- | --- | --- | --- | --- | --- | --- | --- | --- | --- | --- | --- | --- | --- | --- | --- | --- | --- | --- | --- | --- | --- | --- | --- | --- | --- | --- | --- | --- | --- | --- | --- | --- | --- | --- | --- | --- | --- | --- | --- | --- | --- | --- | --- | --- | --- | --- | --- | --- | --- | --- | --- | --- | --- | --- | --- | --- | --- | --- | --- | --- | --- | --- | --- | --- | --- | --- | --- | --- | --- | --- | --- | --- | --- | --- | --- | --- | --- | --- | --- | --- | --- | --- | --- | --- | --- | --- | --- | --- | --- | --- | --- | --- | --- | --- | --- | --- | --- | --- | --- | --- | --- | --- | --- | --- | --- | --- | --- | --- | --- | --- | --- | --- | --- | --- | --- | --- | --- | --- | --- | --- | --- | --- | --- | --- | --- | --- | --- | --- | --- | --- | --- | --- | --- | --- | --- | --- | --- | --- | --- | --- | --- | --- | --- | --- | --- | --- | --- | --- | --- | --- | --- | --- | --- | --- | --- | --- | --- | --- | --- | --- | --- | --- | --- | --- | --- | --- | --- | --- | --- | --- | --- | --- | --- | --- | --- | --- | --- | --- | --- | --- | --- | --- | --- | --- | --- | --- | --- | --- | --- | --- | --- | --- | --- | --- | --- | --- | --- | --- | --- | --- | --- | --- | --- | --- | --- | --- | --- | --- | --- | --- | --- | --- | --- | --- | --- | --- | --- | --- | --- | --- | --- | --- | --- | --- | --- | --- | --- | --- | --- | --- | --- |
| **Authors:** Rahman, A., Malik, A., Sikander, S., Roberts, C. & Creed, F. | | | | | | | | | | | | | | | | | | | | | | | | | | | | | | | | | | | | | | | | | | | | | | | | | | | | | | | | | | | | | | | | | | | | | | | | | | | | | | | | | | | | | | | | | | | | | | | | | | | | | | | | | | | | | | | | | | | | | | | | | | | | | | | | | | | | | | | | | | | | | | | | | | | | | | | | | | | | | | | | | | | | | | | | | | | | | | | | | | | | | | | | | | | | | | | | | | | | | | | | | | | | | | | | | | | | | | | | | | **Year: 2008** | | | | | | | | | | | | | | | | | | | | | | | | | | | | | | | | | | | | | | | | | | | | | | | | | | | | | |  |
| **Summary:** Lay level cadre in Pakistan using Cognitive Based Therapy during home visits for late pregnancy and 1 year postnatal women | | | | | | | | | | | | | | | | | | | | | | | | | | | | | | | | | | | | | | | | | | | | | | | | | | | | | | | | | | | | | | | | | | | | | | | | | | | | | | | | | | | | | | | | | | | | | | | | | | | | | | | | | | | | | | | | | | | | | | | | | | | | | | | | | | | | | | | | | | | | | | | | | | | | | | | | | | | | | | | | | | | | | | | | | | | | | | | | | | | | | | | | | | | | | | | | | | | | | | | | | | | | | | | | | | | | | | | | | | | | | | | | | | | | | | | | | | | | | | | | | | | | | | | | | | | | | | | | | | | | | | | | | | | | | | | |  |
| **Setting** | | | | | | | | | **Design/Method** | | | | | | | | | | | **Population** | | | | | | | | | | | | | | | | | | | | | | | | | | | | | | | | | | | | | | | | | | | | | | | | | **Intervention** | | | | | | | | | | | | | | | | | | | | | | | | | | | **Workforce Characteristics** | | | | | | | | | | | | | | | | | | | | | | | | | | | | | | | | | | | | | | | | | | | | | | | | | | | | | | | | | | | | | | | | | | | | | | | | | | | | | | | | | | | | | | | | | | | | | | | | | | | | | | | | | | | | | | | | | | | | | | | | | | | | | | | | | | | | | | | | | | | | | | | | | | | | | | | | | | | | | | | | | | | | | | | | | | | | | | | |  |
|  |  |  |  |  |  |  |  |  |  |  |  |  |  |  |  |  |  |  |  |  |  |  |  |  |  |  |  |  |  |  |  |  |  |  |  |  |  |  |  |  |  |  |  |  |  |  |  |  |  |  |  |  |  |  |  |  |  |  |  |  |  |  |  |  |  |  |  |  |  |  |  |  |  |  |  |  |  |  |  |  |  |  |  |  |  |  |  |  |  |  |  |  |  |  |  | **Cadres** | | | | | | | | | | | | | | | | | | | | | | | | | | | | | | | | | | | | | **Description** | | | | | | | | | | | | | | | | | | | | | | | | | | | | | | | | | | | | | | | | | | | | | | | | | | **Training** | | | | | | | | | | | | | | | | | | | | | | | | | | | | | | | | | | | | | | | | | | **Supervision** | | | | | | | | | | | | | | | | | | | | | | | | | | | | | | **Misc.** | | | | | | | | | | | | | | | | |  |
| Rural Gujar Khan and Kallar Syedan,  areas in Rawalpindi, Pakistan | | | | | | | | | cRCT | | | | | | | | | | | 903 married women in third trimester aged 16-45 years | | | | | | | | | | | | | | | | | | | | | | | | | | | | | | | | | | | | | | | | | | | | | | | | | “Thinking Healthy Programme” (THP) by LHWs, with 1session/week in last month of pregnancy, 3 in first month postnatal and 1/month for next 9month. Compared to enhanced routine care. | | | | | | | | | | | | | | | | | | | | | | | | | | | Lady Health Workers (LHWs) | | | | | | | | | | | | | | | | | | | | | | | | | | | | | | | | | | | | | Completed secondary school women, recommended by their communities. Responsible for maternal and child health and community education, integrated into health systems, and responsible for approximately 100 households. Receive salary of approx. $340. Provide approx.. 80% of coverage to rural Pakistan. | | | | | | | | | | | | | | | | | | | | | | | | | | | | | | | | | | | | | | | | | | | | | | | | | | Trained in maternal and child health and community education. Trained over 15 months with 3 months classroom based and 12 practical on-the-job. | | | | | | | | | | | | | | | | | | | | | | | | | | | | | | | | | | | | | | | | | | Monthly by LHW supervisors as per usual description, with ratio 1:23. Additional monthly supervision and monitoring by research team. | | | | | | | | | | | | | | | | | | | | | | | | | | | | | | Not permitted to have other income earning activities.  **Quality: 3**  **MMAT cRCT** | | | | | | | | | | | | | | | | |  |
| **CMOCs** | | | | | | | | | | | | | | | | | | | | | | | | | | | | | | | | | | | | | | | | | | | | | | | | | | | | | | | | | | | | | | | | | | | | | | | | | | | | | | | | | | | | | | | | | | | | | | | | | | | | | | | | | | | | | | | | | | | | | | | | | | | | | | | | | | | | | | | | | | | | | | | | | | | | | | | | | | | | | | | | | | | | | | | | | | | | | | | | | | | | | | | | | | | | | | | | | | | | | | | | | | | | | | | | | | | | | | | | | | | | | | | | | | | | | | | | | | | | | | | | | | | | | | | | | | | | | | | | | | | | | | | | | | | | | | | |  |
| **Context** | | | | | | | | | | | | | | | | | **Mechanisms** | | | | | | | | | | | | | | | | | | | | | | | | | | | | | | | | | | | | | | | | | | | | | | | | | | | | | | | | | | | | | | | | | | | | | | | | | | | | | | | | | | | | | | | | | | | | | | | | | | | | | | | | | | | **Outcomes** | | | | | | | | | | | | | | | | | | | | | | | | | | | | | | | | | | | | | | | | | | | | | | **CMOCs** | | | | | | | | | | | | | | | | | | | | | | | | | | | | | | | | | | | | | | | | | | | | | | | | | | | | | | | | | | | | | | | | | | | | | | | | | | | | | | | | | | | | | | | | | | | | | | | | | | | | | |  |
| Already established Union councils.  THP integrated into LHWs regular MCH routine.  Established method of referrals through supervisory pathways.  Already trained, supervised and working lay-level cadres.  LHWs trained specifically on THP by experts and team.  LHWs provided with manual for reference. | | | | | | | | | | | | | | | | | 1. Possible reduction of stigma, since integrated into regular MCH programmes  2. Integrating families into the programme and having them be active members, as well as having ‘homework’ for participants. Involving families not only may help mothers but also encourage more involvement in other aspects.  3. Intervention for women, with only Lady Health Workers, could be more comfortable and more easily for both women and health worker to related and deal with sensitive subjects.  4. Health worker integration and standardized training, support and country recognition. Likely well known and respected in community. Trusted to deliver such an intervention.  5. LHWs already trained and working, integrated into Pakistan’s system, so just seen as an additional training or package.  6. No difference in some child health outcomes, possibility of having LHWs focus be taken away from their usual jobs focusing more on Thinking Healthy Programme  7. LHWs are strongly invested in the community, as being members of and chosen by, and also having this as their full-time job.  8. LHWs treated as a professional in this way may be motivating. | | | | | | | | | | | | | | | | | | | | | | | | | | | | | | | | | | | | | | | | | | | | | | | | | | | | | | | | | | | | | | | | | | | | | | | | | | | | | | | | | | | | | | | | | | | | | | | | | | | | | | | | | | | Reduced depression at 6 months post partum, sustained at 12 months.  No difference in child weight-for-height.  “High” acceptability rate if look at percent of mothers analyzed after 6 and 12 months (same in intervention vs. comparison).  Reduced diarrhoeal disease.  Increase in immunization and use of contraception.  Increase in play-related activities.  LHWs claimed the job size did not increase as a result of delivering this intervention. | | | | | | | | | | | | | | | | | | | | | | | | | | | | | | | | | | | | | | | | | | | | | | 1.Having a rehabilitation worker that you can better relate to (in this case women for women) may increase acceptability of intervention.  2.Family involvement in intervention may increase acceptability of programme and have unintended outcomes.  3. Utilizing an already trained and practicing workforce and adding an additional component of training may help reduce stigma of care, and also acceptability of intervention due to trust community has in their ability do to their pre-established role.  4. Rehabilitation workers identified by the community are more acceptable and may have higher ownership and accountability to the community, which can increase retention.  5. Rehabilitation workers formally integrated and supported by government systems, with prerequisites before being ‘hired’ and structured training/supervision, with financial incentives and having this as full-time job, impacting retention, and professional motivation.  6. Government support (financially and integration into system) of rehabilitation workers.  7. A clear referral systems with connections to more specialized rehabilitation workers.  8. Strong and ongoing support for the health workers from supervisors, research team supervisors half-days monthly to help with stress management | | | | | | | | | | | | | | | | | | | | | | | | | | | | | | | | | | | | | | | | | | | | | | | | | | | | | | | | | | | | | | | | | | | | | | | | | | | | | | | | | | | | | | | | | | | | | | | | | | | | | |  |
| **Title:** Sustainability Criteria for CBR Programmes – Two Case Studies of Provincial Programmes in Vietnam | | | | | | | | | | | | | | | | | | | | | | | | | | | | | | | | | | | | | | | | | | | | | | | | | | | | | | | | | | | | | | | | | | | | | | | | | | | | | | | | | | | | | | | | | | | | | | | | | | | | | | | | | | | | | | | | | | | | | | | | | | | | | | | | | | | | | | | | | | | | | | | | | | | | | | | | | | | | | | | | | | | | | | | | | | | | | | | | | | | | | | | | | | | | | | | | | | | | | | | | | | | | | | | | | | | | | | | | | | | | | | | | | | | | | | | | | | | | | | | | | | | | | | | | | | | | | | | | | | | | | | | | | | | | | | | |  |
| **Authors:** Mijnarends, DM., Pham, D., Swaans, K., Van Brakel, WH. & Wright EP. | | | | | | | | | | | | | | | | | | | | | | | | | | | | | | | | | | | | | | | | | | | | | | | | | | | | | | | | | | | | | | | | | | | | | | | | | | | | | | | | | | | | | | | | | | | | | | | | | | | | | | | | | | | | | | | | | | | | | | | | | | | | | | | | | | | | | | | | | | | | | | | | | | | | | | | | | | | | | | | | | | | | | | | | | | | | | | | | | | | | | | | | | | | | | | | | | | | | | | | | | | | | | | | | | | | | | | | | | | **Year:** 2011 | | | | | | | | | | | | | | | | | | | | | | | | | | | | | | | | | | | | | | | | | | | | | | | | | | | | | |  |
| **Summary:** Human resource needs and challenges in as identified by stakeholders in two CBR programmes in Vietnam. | | | | | | | | | | | | | | | | | | | | | | | | | | | | | | | | | | | | | | | | | | | | | | | | | | | | | | | | | | | | | | | | | | | | | | | | | | | | | | | | | | | | | | | | | | | | | | | | | | | | | | | | | | | | | | | | | | | | | | | | | | | | | | | | | | | | | | | | | | | | | | | | | | | | | | | | | | | | | | | | | | | | | | | | | | | | | | | | | | | | | | | | | | | | | | | | | | | | | | | | | | | | | | | | | | | | | | | | | | | | | | | | | | | | | | | | | | | | | | | | | | | | | | | | | | | | | | | | | | | | | | | | | | | | | | | |  |
| **Setting** | | | | | | | | | **Design/Method** | | | | | | | | | | | | | | | | | | | | | | | | | | | | **Population** | | | | | | | | | | | | | | | | | | | | | | | | | | | | | | | | | | | | | | | | | | | | | | | | | | | | **Intervention** | | | | | | | | | | | | | | | | | | | | | | | | | | | | | | | | | | | | | | | | | | **Workforce Characteristics** | | | | | | | | | | | | | | | | | | | | | | | | | | | | | | | | | | | | | | | | | | | | | | | | | | | | | | | | | | | | | | | | | | | | | | | | | | | | | | | | | | | | | | | | | | | | | | | | | | | | | | | | | | | | | | | | | | | | | | | | | | | | | | | | | | | | | | | | | | | | |  |
|  |  |  |  |  |  |  |  |  |  |  |  |  |  |  |  |  |  |  |  |  |  |  |  |  |  |  |  |  |  |  |  |  |  |  |  |  |  |  |  |  |  |  |  |  |  |  |  |  |  |  |  |  |  |  |  |  |  |  |  |  |  |  |  |  |  |  |  |  |  |  |  |  |  |  |  |  |  |  |  |  |  |  |  |  |  |  |  |  |  |  |  |  |  |  |  |  |  |  |  |  |  |  |  |  |  |  |  |  |  |  |  |  |  |  |  |  |  |  |  |  |  |  |  |  |  |  |  |  |  |  | **Cadres** | | | | | | | | | | | | | | | | | | | | | | | | | | | | | | | | | **Description** | | | | | | | | | | | | | | | | | | | | | | | | | | | | | | | | | **Training** | | | | | | | | | | | | | | | | | | | | | | | | | | | | | | | **Supervision** | | | | | | | | | | | | | | | | | | | | | | | | | | | | | **Misc.** | | | | | | | | | | | | | | |  |
| Two Vietnamese provinces: Dak Lak and Vūng Tàu. | | | | | | | | | Cross-sectional.  Questionnaires, interviews and focus groups  (Interviews n=17, focus groups n=8, questionnaire n=182) | | | | | | | | | | | | | | | | | | | | | | | | | | | | PWD, families of PWD, and CBR workers, programme managers. | | | | | | | | | | | | | | | | | | | | | | | | | | | | | | | | | | | | | | | | | | | | | | | | | | | | Comprehensive following CBR Matrix CBR Programme (in Dak Lak) and medical CBR programme (in Vūng Tàu). Projects regulated at four levels: communal; district; provincial; national. | | | | | | | | | | | | | | | | | | | | | | | | | | | | | | | | | | | | | | | | | | CBR workforce including:  Ministries, steering committee, CBR staff, nurses, doctors, village health workers, PWD and DPOs. | | | | | | | | | | | | | | | | | | | | | | | | | | | | | | | | | Regulated at four levels; communal, district, provincial and national. Stakeholder coordination for management, monitoring of programmes. Steering Committees plan, supervise and coordinate activities. | | | | | | | | | | | | | | | | | | | | | | | | | | | | | | | | | Not specified. | | | | | | | | | | | | | | | | | | | | | | | | | | | | | | | Steering Committees. | | | | | | | | | | | | | | | | | | | | | | | | | | | | | Lack of details on work of CBR workers  **Quality: 3**  **MMAT – MM study** | | | | | | | | | | | | | | |  |
| **CMOCs** | | | | | | | | | | | | | | | | | | | | | | | | | | | | | | | | | | | | | | | | | | | | | | | | | | | | | | | | | | | | | | | | | | | | | | | | | | | | | | | | | | | | | | | | | | | | | | | | | | | | | | | | | | | | | | | | | | | | | | | | | | | | | | | | | | | | | | | | | | | | | | | | | | | | | | | | | | | | | | | | | | | | | | | | | | | | | | | | | | | | | | | | | | | | | | | | | | | | | | | | | | | | | | | | | | | | | | | | | | | | | | | | | | | | | | | | | | | | | | | | | | | | | | | | | | | | | | | | | | | | | | | | | | | | | | | |  |
| **Context** | | | | | | | | | | | | | | | | | | | | | | | | | | | | | | | | | | | | | | | | | | | | | | | | **Mechanisms** | | | | | | | | | | | | | | | | | | | | | | | | | | | | | | | | | | | | | | | | | | | | | | | | | | | | | | | | | | | | | | | | | | | | | **Outcomes** | | | | | | | | | | | | | | | | | | | | | | | | | | | | | | | | | | | | | | | | | | | | | | | | | | | | | | **CMOCs** | | | | | | | | | | | | | | | | | | | | | | | | | | | | | | | | | | | | | | | | | | | | | | | | | | | | | | | | | | | | | | | | | | | | | | | | | | | | | | | | | | | | | | | | | | | | | | | | | | | | |  |
| CBR programmes as early as 1987 in Vietnam and integrated in MoH  Multi-stakeholder coordination for CBR programmes (Figure 1 – Structure of SH in Vietnam CBR), regulated at 4 levels – communal, district, provincial and national.  CBR Steering Committees plan, supervise and coordinate, collaborate with other organizations, at all 4 levels – includes representatives from different sectors (i.e. government, health, education, welfare, WU).  Unequal access to and utilization of health care, especially by ethnic minorities, living in rural areas of Vietnam.  Moving progressively from older style medical rehabilitation projects towards more multi-faceted “comprehensive” ones. | | | | | | | | | | | | | | | | | | | | | | | | | | | | | | | | | | | | | | | | | | | | | | | | 1.Proposed by stakeholders:  - training in managements skills and methodologies  - improve manpower by giving incentives/supportive policies  -One person (i.e. Secretariat) in charge of CBR  - Train programme secretaries at district and community level and CHWs (planning, communication, rehabilitation)  2. Multi-stakeholder coordination (including DPOs, NGOs, and government) may lead to lack of clear job description or roles.  3. High staff turnover possibly due to lack of training and roles/responsibilities.  4. Raising awareness and changing others’ attitudes towards the programme; and listening to community.  5.Strong leadership necessary for sustainable DPOs. | | | | | | | | | | | | | | | | | | | | | | | | | | | | | | | | | | | | | | | | | | | | | | | | | | | | | | | | | | | | | | | | | | | | | High staff turnover, knowledge transfers implications, lack of HR at community levels.  Lack of clear job descriptions.  Identified stable cadre with frequent training/updating of knowledge  Comprehensive programmes identify more training in M&E, economic assistance, training/teaching skills  Collaboration with other sectors, especially the community, in planning, implementing and M&E.  Questions of sustainability, which is defined as sustainability in human resources, organizational setting, social/political environment, and in self-sustaining financial resources.  Labour mobility a continual threat. | | | | | | | | | | | | | | | | | | | | | | | | | | | | | | | | | | | | | | | | | | | | | | | | | | | | | | 1.Skills to self-organize into DPOs, assisted as appropriate by CBR workers.  2.Competencies for sustainability: human resources (stability of a good labour pool), coordination skills, community involvement, and project financing. These are primarily systems-based competencies, as distinct from people-based ones, perhaps.  3. Systems rather than individuals – a stable cadre of CBR workers, with PD systems for continuing development.  4. Everyone in the system needs support and training, e.g., programme secretaries at district and communal level.  5. Appropriate work conditions for the CBR workers, e.g., steady living wages, tenure of employment, possibilities for advancement, and recognition of their intrinsic motivation and worth via non-financial incentives  6. Improper management of finance for CBR work can negatively impact workers, for example through high turnover rates.  7.Lack of participation in decision-making can lower motivation.  8.Mechanisms for institutional memory/knowledge of CBR programmes required  9. Focal CBR person and secretariat at specific levels to increase manpower and accountability mechanisms may aid in retention of staff.  10. Frequent and increased training of staff members to increase retention. | | | | | | | | | | | | | | | | | | | | | | | | | | | | | | | | | | | | | | | | | | | | | | | | | | | | | | | | | | | | | | | | | | | | | | | | | | | | | | | | | | | | | | | | | | | | | | | | | | | | |  |
| **Title:** Training CBR Personnel in South Africa to contribute to the Empowerment of Persons with Disabilities | | | | | | | | | | | | | | | | | | | | | | | | | | | | | | | | | | | | | | | | | | | | | | | | | | | | | | | | | | | | | | | | | | | | | | | | | | | | | | | | | | | | | | | | | | | | | | | | | | | | | | | | | | | | | | | | | | | | | | | | | | | | | | | | | | | | | | | | | | | | | | | | | | | | | | | | | | | | | | | | | | | | | | | | | | | | | | | | | | | | | | | | | | | | | | | | | | | | | | | | | | | | | | | | | | | | | | | | | | | | | | | | | | | | | | | | | | | | | | | | | | | | | | | | | | | | | | | | | | | | | | | | | | | | | | | |  |
| **Authors:** Rule, S. | | | | | | | | | | | | | | | | | | | | | | | | | | | | | | | | | | | | | | | | | | | | | | | | | | | | | | | | | | | | | | | | | | | | | | | | | | | | | | | | | | | | | | | | | | | | | | | | | | | | | | | | | | | | | | | | | | | | | | | | | | | | | | | | | | | | | | | | | | | | | | | | | | | | | | | | | | | | | | | | | | | | | | | | | | | | | | | | | | | | | | | | | | | | | | | | | | | | | | | | | | | | | | | | | | | | | | | | | | **Year:** 2013 | | | | | | | | | | | | | | | | | | | | | | | | | | | | | | | | | | | | | | | | | | | | | | | | | | | | | |  |
| **Summary:** A critical exploration of the training of mid-level CRF workers in a South African non-governmental organization | | | | | | | | | | | | | | | | | | | | | | | | | | | | | | | | | | | | | | | | | | | | | | | | | | | | | | | | | | | | | | | | | | | | | | | | | | | | | | | | | | | | | | | | | | | | | | | | | | | | | | | | | | | | | | | | | | | | | | | | | | | | | | | | | | | | | | | | | | | | | | | | | | | | | | | | | | | | | | | | | | | | | | | | | | | | | | | | | | | | | | | | | | | | | | | | | | | | | | | | | | | | | | | | | | | | | | | | | | | | | | | | | | | | | | | | | | | | | | | | | | | | | | | | | | | | | | | | | | | | | | | | | | | | | | | |  |
| **Setting** | | | | | | | | | **Design/Method** | | | | | | | | | | | | | | | | | | | | | | | | | | | | | | | | | | **Population** | | | | | | | | | | | | | | | | | | | | | | | | | | | | | | | | | | | | | | | | | | | | | **Intervention** | | | | | | | | | | | | | | | | | | | | | | | | | | | | | | | | | | | | | | | | | | | | | | | **Workforce Characteristics** | | | | | | | | | | | | | | | | | | | | | | | | | | | | | | | | | | | | | | | | | | | | | | | | | | | | | | | | | | | | | | | | | | | | | | | | | | | | | | | | | | | | | | | | | | | | | | | | | | | | | | | | | | | | | | | | | | | | | | | | | | | | | | | | | | | | | | | | |  |
|  |  |  |  |  |  |  |  |  |  |  |  |  |  |  |  |  |  |  |  |  |  |  |  |  |  |  |  |  |  |  |  |  |  |  |  |  |  |  |  |  |  |  |  |  |  |  |  |  |  |  |  |  |  |  |  |  |  |  |  |  |  |  |  |  |  |  |  |  |  |  |  |  |  |  |  |  |  |  |  |  |  |  |  |  |  |  |  |  |  |  |  |  |  |  |  |  |  |  |  |  |  |  |  |  |  |  |  |  |  |  |  |  |  |  |  |  |  |  |  |  |  |  |  |  |  |  |  |  |  |  |  |  |  |  | **Cadres** | | | | | | | | | | | | | | | | | | | | | | | | | | | | | **Description** | | | | | | | | | | | | | | | | | | | | | | | | | | | | | | | | | **Training** | | | | | | | | | | | | | | | | | | | | | | | | | | | | | | | | | | | | | **Supervision** | | | | | | | | | | | | | | | | | | | | | | | | | | | | | | **Misc.** | | | | | | | |  |
| KwaZulu Natal, South Africa. | | | | | | | | | Action research -  Three-year period, with one cycle of AR. Including IDI, document analysis, participatory rural appraisal and FGs.. | | | | | | | | | | | | | | | | | | | | | | | | | | | | | | | | | | Staff, students and past graduates from NGO that conducts mid-level CBR training. Clients of CBR , including family members of PWD. | | | | | | | | | | | | | | | | | | | | | | | | | | | | | | | | | | | | | | | | | | | | | CREATE (CBREducation and Training for Empowerment) NGO trains CFRs. Research conducted on staff, current students in 2 year training programme (n=7) and past students. First phase of AR identified needed changes to training, which were then observed for remainder of research. | | | | | | | | | | | | | | | | | | | | | | | | | | | | | | | | | | | | | | | | | | | | | | | Mid-level CBR workers:  Community rehabilitation facilitators (CRFs) | | | | | | | | | | | | | | | | | | | | | | | | | | | | | Directly provide rehabilitation services to PWD and their families, refer to appropriate services, including educational, social etc. Integrated into MoH, completed 10 years schooling prior to training. | | | | | | | | | | | | | | | | | | | | | | | | | | | | | | | | | Two-year course by NGO CREATE with class work covering theory, and practical work in student’s own rural or peri-urban community. | | | | | | | | | | | | | | | | | | | | | | | | | | | | | | | | | | | | | Not reported in detail. | | | | | | | | | | | | | | | | | | | | | | | | | | | | | | Several CBR workers are PWD  **Quality: 4**  **MMAT -** qualitative | | | | | | | |  |
| **CMOCs** | | | | | | | | | | | | | | | | | | | | | | | | | | | | | | | | | | | | | | | | | | | | | | | | | | | | | | | | | | | | | | | | | | | | | | | | | | | | | | | | | | | | | | | | | | | | | | | | | | | | | | | | | | | | | | | | | | | | | | | | | | | | | | | | | | | | | | | | | | | | | | | | | | | | | | | | | | | | | | | | | | | | | | | | | | | | | | | | | | | | | | | | | | | | | | | | | | | | | | | | | | | | | | | | | | | | | | | | | | | | | | | | | | | | | | | | | | | | | | | | | | | | | | | | | | | | | | | | | | | | | | | | | | | | | | | |  |
| **Context** | | | | | | | | | | | | | | | | | | | | | | | | | | | | | | | | | | | | | | | | | | **Mechanisms** | | | | | | | | | | | | | | | | | | | | | | | | | | | | | | | | | | | | | | | | | | | | | | | | | | | | | | | | | | | | | | | | | | | | | | | | | | | | | | | | | | | | | | | | | **Outcomes** | | | | | | | | | | | | | | | | | | | | | | | | | | | | | | | | | | | | | | | | | | | | | | | **CMOCs** | | | | | | | | | | | | | | | | | | | | | | | | | | | | | | | | | | | | | | | | | | | | | | | | | | | | | | | | | | | | | | | | | | | | | | | | | | | | | | | | | | | | | | | | | | | | | |  |
| Study done with CRFs previously trained in old model, which had little emphasis on social model/empowerment training.  New model had more teaching on social model, including theoretical perspective, with a changed ethos to include more overt emphasis on empowerment and emancipatory approaches in CBR.  Content of (new) training utilized Freire’s concepts of problematization and conscientization, which had course facilitators with disabilities running training workshops. | | | | | | | | | | | | | | | | | | | | | | | | | | | | | | | | | | | | | | | | | | 1.Usually culturally relevant themes to teach on social inclusion and empowerment, in this case Ubuntu, to better communicate teachings.  2. Theoretical teachings on social model can help CBR workers identify their techniques in empowerment and recognition of oppression.  3. Teaching social model and empowerment in training can help workers better relate to PLWD and work in more collaborative manner.  4. Training on empowerment and social model of CBR as well as health leaves students more articulate and aware of structural barriers PWD face.  5. More rural workers may face difficulties in executing work, especially group gatherings and collective action.  6. CBR workers engaged in social activities may face resistance from current social structures and political forces. | | | | | | | | | | | | | | | | | | | | | | | | | | | | | | | | | | | | | | | | | | | | | | | | | | | | | | | | | | | | | | | | | | | | | | | | | | | | | | | | | | | | | | | | | CRF workers identify oppressions persons with disabilities face, however increased awareness and identification of these when trained specifically on discrimination.  Training resulted in CFR workers working with, not for, PWD, with potential reference to Ubuntu in this context.  More teaching on social model, including theoretical perspective.  Collective action and organizing PWD difficult in rural areas.  Previously trained CRFs were less able to articulate how to empower than their later-trained counterparts. | | | | | | | | | | | | | | | | | | | | | | | | | | | | | | | | | | | | | | | | | | | | | | | 1.CBR workers’ competency should include ability to recognize oppression PLWD have, from different levels.  2. Long (2 year) training courses that have components focused on empowerment and oppression, centered on the social model of disability and the CBR Matrix.  3. The cross-sectorial nature of CBR requires multi-skilled personnel and the careful planning of appropriate training.  4. CBR training that has a focus on social justice enable the workforce to recognize discrimination. Empowerment and advocacy skills provide tools for workers to make positive changes in relation to the discrimination.  5. Traditional values or culturally relevant teaching and themes to assist in development of competencies.  6. PWD as trainers for CBR and also CBR workers should be encouraged. | | | | | | | | | | | | | | | | | | | | | | | | | | | | | | | | | | | | | | | | | | | | | | | | | | | | | | | | | | | | | | | | | | | | | | | | | | | | | | | | | | | | | | | | | | | | | |  |
| **Title:** Provision of mental health services in resource-poor settings: a randomized trial comparing counseling with routine medical treatment in North Afghanistan (Mazar-e-Sharif) | | | | | | | | | | | | | | | | | | | | | | | | | | | | | | | | | | | | | | | | | | | | | | | | | | | | | | | | | | | | | | | | | | | | | | | | | | | | | | | | | | | | | | | | | | | | | | | | | | | | | | | | | | | | | | | | | | | | | | | | | | | | | | | | | | | | | | | | | | | | | | | | | | | | | | | | | | | | | | | | | | | | | | | | | | | | | | | | | | | | | | | | | | | | | | | | | | | | | | | | | | | | | | | | | | | | | | | | | | | | | | | | | | | | | | | | | | | | | | | | | | | | | | | | | | | | | | | | | | | | | | | | | | | | | | | |  |
| **Authors:** Ayoughi, S., Missmahl, I., Weierstall, R. & Elbert, T. | | | | | | | | | | | | | | | | | | | | | | | | | | | | | | | | | | | | | | | | | | | | | | | | | | | | | | | | | | | | | | | | | | | | | | | | | | | | | | | | | | | | | | | | | | | | | | | | | | | | | | | | | | | | | | | | | | | | | | | | | | | | | | | | | | | | | | | | | | | | | | | | | | | | | | | | | | | | | | | | | | | | | | | | | | | | | | | | | | | | | | | | | | | | | | | | | | | | | | | | | | | | | | | | | | | | | | | | | | **Year:** 2012 | | | | | | | | | | | | | | | | | | | | | | | | | | | | | | | | | | | | | | | | | | | | | | | | | | | | | |  |
| **Summary:** Psychosocial counseling intervention for persons with mental illness delivered by lay health workers trained by local physicians | | | | | | | | | | | | | | | | | | | | | | | | | | | | | | | | | | | | | | | | | | | | | | | | | | | | | | | | | | | | | | | | | | | | | | | | | | | | | | | | | | | | | | | | | | | | | | | | | | | | | | | | | | | | | | | | | | | | | | | | | | | | | | | | | | | | | | | | | | | | | | | | | | | | | | | | | | | | | | | | | | | | | | | | | | | | | | | | | | | | | | | | | | | | | | | | | | | | | | | | | | | | | | | | | | | | | | | | | | | | | | | | | | | | | | | | | | | | | | | | | | | | | | | | | | | | | | | | | | | | | | | | | | | | | | | |  |
| **Setting** | | | | | | | | | **Design/**  **Method** | | | | | | | | | | | | | | | | | | | | | **Population** | | | | | | | | | | | | | | | | | | | | | | | | | | | | | | | | | | | | | | | | | | | | | | | | | | | | **Intervention** | | | | | | | | | | | | | | | | | | | | | | | | | | | | | | | | | | | | | | | | | | | | | | | | | | | | | | **Workforce Characteristics** | | | | | | | | | | | | | | | | | | | | | | | | | | | | | | | | | | | | | | | | | | | | | | | | | | | | | | | | | | | | | | | | | | | | | | | | | | | | | | | | | | | | | | | | | | | | | | | | | | | | | | | | | | | | | | | | | | | | | | | | | | | | | | | | | | | | | | | |  |
|  |  |  |  |  |  |  |  |  |  |  |  |  |  |  |  |  |  |  |  |  |  |  |  |  |  |  |  |  |  |  |  |  |  |  |  |  |  |  |  |  |  |  |  |  |  |  |  |  |  |  |  |  |  |  |  |  |  |  |  |  |  |  |  |  |  |  |  |  |  |  |  |  |  |  |  |  |  |  |  |  |  |  |  |  |  |  |  |  |  |  |  |  |  |  |  |  |  |  |  |  |  |  |  |  |  |  |  |  |  |  |  |  |  |  |  |  |  |  |  |  |  |  |  |  |  |  |  |  |  |  |  |  |  |  |  | **Cadres** | | | | | | | | | | | | | | | | | | | | | | | | | | | | **Description** | | | | | | | | | | | | | | | | | | | | | | | | | | | | | | | | | | | **Training** | | | | | | | | | | | | | | | | | | | | | | | | | | | | | | | | | | | | | | | **Supervision** | | | | | | | | | | | | | | | | | | | | | | | | | | | | | **Misc.** | | | | |  |
| Mazar-e-Sharif, Balkh province, Afghanistan | | | | | | | | | Randomised before and after. | | | | | | | | | | | | | | | | | | | | | 66 mental health patients recruited from PHC, referred by independent physicians. | | | | | | | | | | | | | | | | | | | | | | | | | | | | | | | | | | | | | | | | | | | | | | | | | | | | Medication group compared to psychosocial counseling group. Counseling in accordance with treatment guidelines of the manual “Professional Package for Psychosocial Counselors with in the BPHS in Afghanistan”. Five 45-60 minute sessions over 5 weeks, and up to 8 additional if supervisor agreed. | | | | | | | | | | | | | | | | | | | | | | | | | | | | | | | | | | | | | | | | | | | | | | | | | | | | | | Local physicians, and lay mental health counselors | | | | | | | | | | | | | | | | | | | | | | | | | | | | Local physicians trained as psychosocial counselors in 2-year programme for psychosocial counseling then trained lay mental health counselors (Afghan men and women). Lay mental health counselors took employment at local health centres and counseling centre. | | | | | | | | | | | | | | | | | | | | | | | | | | | | | | | | | | | 3.5 months of intensive training (n=30) by previously trained local physicians, with examination for quality. | | | | | | | | | | | | | | | | | | | | | | | | | | | | | | | | | | | | | | | New counselors employed by health centre. | | | | | | | | | | | | | | | | | | | | | | | | | | | | | **Quality:4**  **MMAT-** Quant2 | | | | |  |
| **CMOCs** | | | | | | | | | | | | | | | | | | | | | | | | | | | | | | | | | | | | | | | | | | | | | | | | | | | | | | | | | | | | | | | | | | | | | | | | | | | | | | | | | | | | | | | | | | | | | | | | | | | | | | | | | | | | | | | | | | | | | | | | | | | | | | | | | | | | | | | | | | | | | | | | | | | | | | | | | | | | | | | | | | | | | | | | | | | | | | | | | | | | | | | | | | | | | | | | | | | | | | | | | | | | | | | | | | | | | | | | | | | | | | | | | | | | | | | | | | | | | | | | | | | | | | | | | | | | | | | | | | | | | | | | | | | | | | | |  |
| **Context** | | | | | | | | | | | | | | | | | | | | | | | | | | | | | | | | | | | | | | | | | | **Mechanisms** | | | | | | | | | | | | | | | | | | | | | | | | | | | | | | | | | | | | | | | | | | | | | | | | | | | | | | | | | | | | | | | | | | | | | | | | | | | | | **Outcomes** | | | | | | | | | | | | | | | | | | | | | | | | | | | | | | | | | | | | | | | | | | | | | | | | | | | | | | | | | | | | | | | | | | **CMOCs** | | | | | | | | | | | | | | | | | | | | | | | | | | | | | | | | | | | | | | | | | | | | | | | | | | | | | | | | | | | | | | | | | | | | | | | | | | | | | | | | | | | | | | |  |
| Testing whether short-term training for medical doctors, to deliver counseling over medical treatment could make a difference to levels of depression and anxiety.  Counseling patients were interviewed and counseled by individuals of the same sex.  Diagnostic interviews were conducted by two experienced local counselors (1 male 1 female) and two international experts (both female). | | | | | | | | | | | | | | | | | | | | | | | | | | | | | | | | | | | | | | | | | | 1. Lay counselors from the community with specific training can be effective in delivering psychosocial interventions for individuals with depression, anxiety, and psychosocial issues.  2. Lay counselors that have a strong support and supervision mechanism, clear areas for referral and follow-up may be capable of providing psychosocial support.  3. Counselors of the same gender of their patients may assist in patients being more comfortable and acceptable of an intervention (for mental health interventions), especially in certain contexts.  4. Patients requiring frequent visits to a health centre may have a higher drop out rate due to time and expenses associated with travel depending on the distance. | | | | | | | | | | | | | | | | | | | | | | | | | | | | | | | | | | | | | | | | | | | | | | | | | | | | | | | | | | | | | | | | | | | | | | | | | | | | | In counseling, 8.8% dropped out compared to 6.3% in control (routine medical) group – with reasoning being not being able to afford the time to continuously visit the distantly located health centre.  Counseling group showed significantly lower HSCL depression scores and anxiety compared to control group.  Reduction in psychosocial stressors and an enhancement of coping strategies improved in counseling group but no improvements reported in control.  The improvement was on both DVs, and there was even a mediation test, which indicated that the treatment may have worked in part (they were partial mediations between treatment and both DVs) because it alleviated psychosocial stressors like honour and shame issues. | | | | | | | | | | | | | | | | | | | | | | | | | | | | | | | | | | | | | | | | | | | | | | | | | | | | | | | | | | | | | | | | | | 1. Lay counselors from the community with specific training can be effective in delivering psychosocial interventions for individuals with depression, anxiety, and psychosocial issues.  2. Services should be brought as close to individuals as possible as location of treatment may influence uptake of services.  3. Psychosocial support competencies are implicated in an effective intervention for clients, which, involves building capacity and competency in a specially trained cadre or group.  4. The cultural context of rehabilitation work must be considered when preparing for the workforce, or assigning individual staff to a particular person. | | | | | | | | | | | | | | | | | | | | | | | | | | | | | | | | | | | | | | | | | | | | | | | | | | | | | | | | | | | | | | | | | | | | | | | | | | | | | | | | | | | | | | |  |
| **Title:** The development of a lay health worker delivered collaborative community based intervention for people with schizophrenia in India | | | | | | | | | | | | | | | | | | | | | | | | | | | | | | | | | | | | | | | | | | | | | | | | | | | | | | | | | | | | | | | | | | | | | | | | | | | | | | | | | | | | | | | | | | | | | | | | | | | | | | | | | | | | | | | | | | | | | | | | | | | | | | | | | | | | | | | | | | | | | | | | | | | | | | | | | | | | | | | | | | | | | | | | | | | | | | | | | | | | | | | | | | | | | | | | | | | | | | | | | | | | | | | | | | | | | | | | | | | | | | | | | | | | | | | | | | | | | | | | | | | | | | | | | | | | | | | | | | | | | | | | | | | | | | | |  |
| **Authors:** Balaji, M., Chatterjee, S., Kischorke, M., Rangaswamy, T., Chavan, A., Dabholkar, H., Dakshin, L., Kumar, P., John S., Thronicroft, G. & Patel V. | | | | | | | | | | | | | | | | | | | | | | | | | | | | | | | | | | | | | | | | | | | | | | | | | | | | | | | | | | | | | | | | | | | | | | | | | | | | | | | | | | | | | | | | | | | | | | | | | | | | | | | | | | | | | | | | | | | | | | | | | | | | | | | | | | | | | | | | | | | | | | | | | | | | | | | | | | | | | | | | | | | | | | | | | | | | | | | | | | | | | | | | | | | | | | | | | | | | | | | | | | | | | | | | | | | | | | | | | | **Year:** 2012 | | | | | | | | | | | | | | | | | | | | | | | | | | | | | | | | | | | | | | | | | | | | | | | | | | | | | |  |
| **Summary:** The development of a lay health worker delivered community based intervention for schizophrenia is discussed with evidence for programme adjustment. | | | | | | | | | | | | | | | | | | | | | | | | | | | | | | | | | | | | | | | | | | | | | | | | | | | | | | | | | | | | | | | | | | | | | | | | | | | | | | | | | | | | | | | | | | | | | | | | | | | | | | | | | | | | | | | | | | | | | | | | | | | | | | | | | | | | | | | | | | | | | | | | | | | | | | | | | | | | | | | | | | | | | | | | | | | | | | | | | | | | | | | | | | | | | | | | | | | | | | | | | | | | | | | | | | | | | | | | | | | | | | | | | | | | | | | | | | | | | | | | | | | | | | | | | | | | | | | | | | | | | | | | | | | | | | | |  |
| **Setting** | | | | | | | | | | | | **Design/**  **Method** | | | | | | | | | | | | | | | | | | | **Population** | | | | | | | | | | | | | | | | | | | | | | | | | | | **Intervention** | | | | | | | | | | | | | | | | | | | | | | | | | | | | | | | | | | | | | | | | | | | | | | | | | | | | | **Workforce Characteristics** | | | | | | | | | | | | | | | | | | | | | | | | | | | | | | | | | | | | | | | | | | | | | | | | | | | | | | | | | | | | | | | | | | | | | | | | | | | | | | | | | | | | | | | | | | | | | | | | | | | | | | | | | | | | | | | | | | | | | | | | | | | | | | | | | | | | | | | | | | | | | | | | | | | | | | | | | | | | | | | | |  |
|  |  |  |  |  |  |  |  |  |  |  |  |  |  |  |  |  |  |  |  |  |  |  |  |  |  |  |  |  |  |  |  |  |  |  |  |  |  |  |  |  |  |  |  |  |  |  |  |  |  |  |  |  |  |  |  |  |  |  |  |  |  |  |  |  |  |  |  |  |  |  |  |  |  |  |  |  |  |  |  |  |  |  |  |  |  |  |  |  |  |  |  |  |  |  |  |  |  |  |  |  |  |  |  |  |  |  |  |  |  |  | **Cadres** | | | | | | | | | | | | | | | | | | | | | | **Description** | | | | | | | | | | | | | | | | | | | | | | | | | | | | | | | | | **Training** | | | | | | | | | | | | | | | | | | | | | | | | | | | | | | | | | | | | **Supervision** | | | | | | | | | | | | | | | | | | | | | | | | | | | | | | | | | | | | | | | | | **Misc.** | | | | | | | | | | | | | | | | | | | | | | | | | | | | |  |
| Goa, India; Satara in western Maharashtra, India; Kanchipuram in northeastern Tamil Nadu, India. | | | | | | | | | | | | Formative case evaluation.  IDIs with 32 people with schizophrenia and 38 primary caregivers | | | | | | | | | | | | | | | | | | | People with schizophrenia and their caregivers. 30 individuals received intervention. | | | | | | | | | | | | | | | | | | | | | | | | | | | Four phases: identifying gaps; identifying intervention components; evaluating acceptability; piloting delivery of intervention. Intervention – (0-3mths engagement) weekly home sessions, (4-7mths stabilization) visits every 2-4 weeks; (8-12mths maintenance phase)  CLHW expected to conduct an average of 22 home based sessions over 12 months. | | | | | | | | | | | | | | | | | | | | | | | | | | | | | | | | | | | | | | | | | | | | | | | | | | | | | Community level health workers (CLHWs) | | | | | | | | | | | | | | | | | | | | | | Recruited locally, having minimum 10 years of schooling, with no prior training in mental health, commitment to helping people with mental illness. Working with 15-25 people with schizophrenia and their families. | | | | | | | | | | | | | | | | | | | | | | | | | | | | | | | | | 40-50 days of training with a team health specialists, with a variety of methods including: films, documentaries, quizzes, dramas, debates and games. Assessments were done at the end of each training module. Training varied from sites depending on the needs assessment from local area. | | | | | | | | | | | | | | | | | | | | | | | | | | | | | | | | | | | | Supervision protocols were developed which included on-site supervision; quarterly reviews; fortnightly review with the intervention team; monthly group meetings with CLHWs and the intervention coordinator. | | | | | | | | | | | | | | | | | | | | | | | | | | | | | | | | | | | | | | | | | Intervention coordinators via advertisements on websites and newspapers.  CLHWs by advertisements by placing advertisements with partnering NGOs, contacting employment agencies, local newspapers, and approaching local governments. Interviewed candidate.  **QUALITY-NA –Descriptive case study** | | | | | | | | | | | | | | | | | | | | | | | | | | | | |  |
| **CMOCs** | | | | | | | | | | | | | | | | | | | | | | | | | | | | | | | | | | | | | | | | | | | | | | | | | | | | | | | | | | | | | | | | | | | | | | | | | | | | | | | | | | | | | | | | | | | | | | | | | | | | | | | | | | | | | | | | | | | | | | | | | | | | | | | | | | | | | | | | | | | | | | | | | | | | | | | | | | | | | | | | | | | | | | | | | | | | | | | | | | | | | | | | | | | | | | | | | | | | | | | | | | | | | | | | | | | | | | | | | | | | | | | | | | | | | | | | | | | | | | | | | | | | | | | | | | | | | | | | | | | | | | | | | | | | | | | |  |
| **Context** | | | | | | | | | | | | | | | | | | | | | | | | | | | | | | | | | | | | | | | | | | | | | | | | | | | | | | | | | | | | | | | | | | | | | | | **Mechanisms** | | | | | | | | | | | | | | | | | | | | | | | | | | | | | | | | | | | | | | | | | | | | | | | | | | | | | | | | | | | | | | | | | | **Outcomes** | | | | | | | | | | | | | | | | | | | | | | | | | | | | | | | | | | | | | | | | | | | | | | | | | | | | | | | | | | | | | | | **CMOCs** | | | | | | | | | | | | | | | | | | | | | | | | | | | | | | | | | | | | | | | | | | | | | | | | | | | | | | | | | | | | | | | | | | | | | | | |  |
| Pre-intervention in all sites, treatment provided in health care facilities by mainly and exclusively psychiatrists – with primary focus being in symptom reduction through medication.  Intervention: 3-tiered teams (of CLHW, working with 15-25 people with schizophrenia and their caregivers); intervention coordinators (e.g., social workers); and leadership (by treating psychiatrists). Evidence-based, improving awareness, promoting social inclusion and vocational rehabilitation, in collaboration with families, communities and psychiatrists.  Treating psychiatrist to provide necessary pharmacological treatment, development of intervention, clinical leadership and supervision.  *A*ssist people with schizophrenia and their caregivers to acquire skills to plan for recovery  Delivery and selection of specific components of intervention guided by the unique needs of each individual and their families through needs assessment and flexible to change over time | | | | | | | | | | | | | | | | | | | | | | | | | | | | | | | | | | | | | | | | | | | | | | | | | | | | | | | | | | | | | | | | | | | | | | | 1.Initial needs assessment of potential user stakeholders to identify their requirements of a health worker.  2. Users may prioritize different aspects of health services (hence the need for a needs assessment) such as a worker of the same sex, or a worker with previous experience, whether work or personal.  3. Social skills training, referral training and using the CBR Matrix (specifically health and livelihoods) may influence individuals to recognize the importance of the work and intervention, as well as the acceptability of the CLHWs.  4. Fear of disclosure may inhibit the success of CBIs, especially for sensitive rehabilitation issues, and should be considered when designing an intervention and as a central component to training lay workers. | | | | | | | | | | | | | | | | | | | | | | | | | | | | | | | | | | | | | | | | | | | | | | | | | | | | | | | | | | | | | | | | | | Acceptability of the CBI, though patients also recognised medicines and essential.  Patients had concern over the stigma that a CBI intervention could produce.  Participants recognised the importance of family involvement in the CBI intervention.  Characteristics of CLHWs were identified including: understanding; calm; friendly; polite; patient.  CLHW’s education was seen as important, but more pressing was their knowledge and previous experience with the subject.  Some patients identified the need of having a same sex counselor, which was context specific.  Concerns around religion, or obligation to continue treatment.  CLHW had difficulty with developing treatment plans, social skills training and addressing stigma experiences reported by participants. | | | | | | | | | | | | | | | | | | | | | | | | | | | | | | | | | | | | | | | | | | | | | | | | | | | | | | | | | | | | | | | 1. Supervision of CLHWs by intervention coordinators, who are mental health specialists, to support, train and monitor quality.  2.Treating psychiatrist to provide necessary pharmacological treatment, development of intervention, clinical leadership and supervision.  3. Initial needs assessment of potential user stakeholders to identify their requirements of a health worker.  4. An initial needs assessment, both of the context and the individual, can lead to produce recommendations for counseling that can enhance the intervention and make it more acceptable to the patients.  5. Local recruitment of community-based health workers may influence acceptability, and increase rapport with clients.  6. Supervision of community-based health workers should be done in a collaborative manner with specialists in the area as well as managers and peers. | | | | | | | | | | | | | | | | | | | | | | | | | | | | | | | | | | | | | | | | | | | | | | | | | | | | | | | | | | | | | | | | | | | | | | | |  |
| **Title:** The Role of Community Health Workers in the Mongolian CBR Programme | | | | | | | | | | | | | | | | | | | | | | | | | | | | | | | | | | | | | | | | | | | | | | | | | | | | | | | | | | | | | | | | | | | | | | | | | | | | | | | | | | | | | | | | | | | | | | | | | | | | | | | | | | | | | | | | | | | | | | | | | | | | | | | | | | | | | | | | | | | | | | | | | | | | | | | | | | | | | | | | | | | | | | | | | | | | | | | | | | | | | | | | | | | | | | | | | | | | | | | | | | | | | | | | | | | | | | | | | | | | | | | | | | | | | | | | | | | | | | | | | | | | | | | | | | | | | | | | | | | | | | | | | | | | | | | |  |
| **Authors:** Como, E. & Batdulam, T. | | | | | | | | | | | | | | | | | | | | | | | | | | | | | | | | | | | | | | | | | | | | | | | | | | | | | | | | | | | | | | | | | | | | | | | | | | | | | | | | | | | | | | | | | | | | | | | | | | | | | | | | | | | | | | | | | | | | | | | | | | | | | | | | | | | | | | | | | | | | | | | | | | | | | | | | | | | | | | | | | | | | | | | | | | | | | | | | | | | | | | | | | | | | | | | | | | | | | | | | | | | | | | | | | | | | | | | | | | **Year:** 2012 | | | | | | | | | | | | | | | | | | | | | | | | | | | | | | | | | | | | | | | | | | | | | | | | | | | | | |  |
| **Summary:** Role of CHWs as CBR workers, including description of duties and challenges faced when implementing CBR | | | | | | | | | | | | | | | | | | | | | | | | | | | | | | | | | | | | | | | | | | | | | | | | | | | | | | | | | | | | | | | | | | | | | | | | | | | | | | | | | | | | | | | | | | | | | | | | | | | | | | | | | | | | | | | | | | | | | | | | | | | | | | | | | | | | | | | | | | | | | | | | | | | | | | | | | | | | | | | | | | | | | | | | | | | | | | | | | | | | | | | | | | | | | | | | | | | | | | | | | | | | | | | | | | | | | | | | | | | | | | | | | | | | | | | | | | | | | | | | | | | | | | | | | | | | | | | | | | | | | | | | | | | | | | | |  |
| **Setting** | | | | | | | | | **Design/Methods** | | | | | | | | | | | | | | | | | | | | | | | | | | | | | | | | | **Population** | | | | | | | | | | | | | | | | | | | | | | | | | | | | | | | | | | | | **Intervention** | | | | | | | | | | | | | | | | | | | | | | | | | | | | | | **Workforce Characteristics** | | | | | | | | | | | | | | | | | | | | | | | | | | | | | | | | | | | | | | | | | | | | | | | | | | | | | | | | | | | | | | | | | | | | | | | | | | | | | | | | | | | | | | | | | | | | | | | | | | | | | | | | | | | | | | | | | | | | | | | | | | | | | | | | | | | | | | | | | | | | | | | | | | | | | | | | | | | | | | | | | | | |  |
|  |  |  |  |  |  |  |  |  |  |  |  |  |  |  |  |  |  |  |  |  |  |  |  |  |  |  |  |  |  |  |  |  |  |  |  |  |  |  |  |  |  |  |  |  |  |  |  |  |  |  |  |  |  |  |  |  |  |  |  |  |  |  |  |  |  |  |  |  |  |  |  |  |  |  |  |  |  |  |  |  |  |  |  |  |  |  |  |  |  |  |  |  |  |  |  |  |  |  |  |  |  |  |  |  |  |  |  | **Cadres** | | | | | | | | | | | | | | | | | | | | | | | | | | | | | | | | **Description** | | | | | | | | | | | | | | | | | | | | | | | | | | | | | | | | | | | | | | **Training** | | | | | | | | | | | | | | | | | | | | | | | | | | | | | | | | | | | | | | | | | | | | | | | **Supervision** | | | | | | | | | | | | | | | | | | | | | | | | | | | **Misc.** | | | | | | | | | | | | | | | | | | | |  |
| Hentii, Dornod, Suhbataar in rural Eastern Mongolia. | | | | | | | | | Qualitative and descriptive field study.  Semi-structured interviews | | | | | | | | | | | | | | | | | | | | | | | | | | | | | | | | | 16 Feldshers (CHWs), | | | | | | | | | | | | | | | | | | | | | | | | | | | | | | | | | | | | Previously working CHWs (feldshers) were trained as CBR workers when programme initiated. Feldsher responsible for all PHC at community levels | | | | | | | | | | | | | | | | | | | | | | | | | | | | | | Feldshers | | | | | | | | | | | | | | | | | | | | | | | | | | | | | | | | Responsible for all PHC in community. They act as the connection between the community and local Governors and assist in connections and support of activities | | | | | | | | | | | | | | | | | | | | | | | | | | | | | | | | | | | | | | 10 day training none of the feldshers had experience in supporting persons with disabilities beyond standard PHC. | | | | | | | | | | | | | | | | | | | | | | | | | | | | | | | | | | | | | | | | | | | | | | | Not discussed | | | | | | | | | | | | | | | | | | | | | | | | | | | **Quality: 2.5**  MMAT – Qualitative  Data analysis information lacking | | | | | | | | | | | | | | | | | | | |  |
| **CMOCs** | | | | | | | | | | | | | | | | | | | | | | | | | | | | | | | | | | | | | | | | | | | | | | | | | | | | | | | | | | | | | | | | | | | | | | | | | | | | | | | | | | | | | | | | | | | | | | | | | | | | | | | | | | | | | | | | | | | | | | | | | | | | | | | | | | | | | | | | | | | | | | | | | | | | | | | | | | | | | | | | | | | | | | | | | | | | | | | | | | | | | | | | | | | | | | | | | | | | | | | | | | | | | | | | | | | | | | | | | | | | | | | | | | | | | | | | | | | | | | | | | | | | | | | | | | | | | | | | | | | | | | | | | | | | | | | |  |
| **Context** | | | | | | | | | | | | | | | | | | | | | | | | | | | | | | | | | | | | | | | | | | | | | | | | | | | **Mechanisms** | | | | | | | | | | | | | | | | | | | | | | | | | | | | | | | | | | | | | | | | | | | | | | | | | | | | | | | | | | **Outcomes** | | | | | | | | | | | | | | | | | | | | | | | | | | | | | | | | | | | | | | | | | | | | | | | | | | | | | | | | | | | | | | | | | | | | | | | | | | | | | | | | | | | | | | | | | | | | | | | | | | | **CMOCs** | | | | | | | | | | | | | | | | | | | | | | | | | | | | | | | | | | | | | | | | | | | | | | | | | | | | | | | | | | | | | | | |  |
| Mongolia has had CBR programmes since the 1990s, supported by WHO and NGO AIFO. CBR introduced to the Eastern part of Mongolia in 2007 however.  Feldsher responsible for all PHC at community levels; sometimes used for data collection; sometimes as a replacement for health staff at health facility or filing other feldshers vacancies. They act as the connection between the community and local Governors and assist in connections and support of activities; playing an important social role in communities.  15/16 feldshers were female; 15 had graduated from nursing school at least 10 years prior; 15 had at least 10 years experience as a feldshers. | | | | | | | | | | | | | | | | | | | | | | | | | | | | | | | | | | | | | | | | | | | | | | | | | | | 1.Strong history of CBR and examples to following for the development of programme; support from the MoH for CBR.  2. CHWs that are integrated into health systems and are (highly) training and re acceptable to learning CBR work.  3. When tasking shifting CBR, without specific training on sensitivity/social aspects of disability including advocacy and disability prevention, workers may not be strong in these areas.  4. Task shifting of currently working health workers to provide disability rehabilitation is highly acceptable, however specific training on recognition, testing and rehabilitation must be incorporated. | | | | | | | | | | | | | | | | | | | | | | | | | | | | | | | | | | | | | | | | | | | | | | | | | | | | | | | | | | Feldshers face problems of transportation, lack of equipment/materials/uniforms. They are required to make routine house visits ideally every 1-2 months; however due to transport, terrain and geographic constraints, these visits are usually short and occur only 2-3 times per year.  Feldshers rarely discussed their role as advocates for persons with disabilities and CBR, or the role of the community involvement and development in relation to CBR.  Post-training Feldshers recognised the importance of prevention and rehabilitation; need for advocacy for PWD for equal opportunities; integrated intervention approach in accordance with the CBR Matrix.  Feldshers accepted CBR as a valuable component to their general role of PHC. Challenges emerged in the areas of:  -reaching all families  -testing the abilities of fellow community members for disabilities  - record keeping and reporting (according to the CBR Coordinators)  - providing rehabilitation for non-physical disabilities  - training and motivating PWD and their families to continue rehabilitation.  Areas such as social inclusion, empowerment and promotion of livelihoods was poorly understood.  Feldshers lacked commination skills towards families and communities, and especially in relation to communication with and support to persons with sensory or intellectual disabilities. | | | | | | | | | | | | | | | | | | | | | | | | | | | | | | | | | | | | | | | | | | | | | | | | | | | | | | | | | | | | | | | | | | | | | | | | | | | | | | | | | | | | | | | | | | | | | | | | | | | 1.Knowledge and skills in health, education, livelihood, social and empowerment. The later ones of these showed the most need to be trained.  2.Introducing CBR skills and CBR Matrix components into health workers training can impact positively on their views towards persons with disabilities.  3. Transportation for health workers, especially in rural areas, can be a factor that hinders access to workers and implementation of rehabilitation programmes.  4. Health workers can acquire relatively solid knowledge on physical disabilities and work with patients comfortably; however, they may be less comfortable with sensory and mental disabilities and not as confident with their rehabilitation knowledge. | | | | | | | | | | | | | | | | | | | | | | | | | | | | | | | | | | | | | | | | | | | | | | | | | | | | | | | | | | | | | | | |  |
| **Title:** The impact of community based rehabilitation as implemented by community rehabilitation facilitators on people with disabilities, their families and communities within South Africa | | | | | | | | | | | | | | | | | | | | | | | | | | | | | | | | | | | | | | | | | | | | | | | | | | | | | | | | | | | | | | | | | | | | | | | | | | | | | | | | | | | | | | | | | | | | | | | | | | | | | | | | | | | | | | | | | | | | | | | | | | | | | | | | | | | | | | | | | | | | | | | | | | | | | | | | | | | | | | | | | | | | | | | | | | | | | | | | | | | | | | | | | | | | | | | | | | | | | | | | | | | | | | | | | | | | | | | | | | | | | | | | | | | | | | | | | | | | | | | | | | | | | | | | | | | | | | | | | | | | | | | | | | | | | | | |  |
| **Authors:** Chappell, P. & Johannsmeier, C. | | | | | | | | | | | | | | | | | | | | | | | | | | | | | | | | | | | | | | | | | | | | | | | | | | | | | | | | | | | | | | | | | | | | | | | | | | | | | | | | | | | | | | | | | | | | | | | | | | | | | | | | | | | | | | | | | | | | | | | | | | | | | | | | | | | | | | | | | | | | | | | | | | | | | | | | | | | | | | | | | | | | | | | | | | | | | | | | | | | | | | | | | | | | | | | | | | | | | | | | | | | | | | | | | | | | | | | | | | **Year:** 2009 | | | | | | | | | | | | | | | | | | | | | | | | | | | | | | | | | | | | | | | | | | | | | | | | | | | | | |  |
| **Summary:** Impact of rehabilitation by mid-level cadre of rehabilitation workers on persons with disabilities and their families | | | | | | | | | | | | | | | | | | | | | | | | | | | | | | | | | | | | | | | | | | | | | | | | | | | | | | | | | | | | | | | | | | | | | | | | | | | | | | | | | | | | | | | | | | | | | | | | | | | | | | | | | | | | | | | | | | | | | | | | | | | | | | | | | | | | | | | | | | | | | | | | | | | | | | | | | | | | | | | | | | | | | | | | | | | | | | | | | | | | | | | | | | | | | | | | | | | | | | | | | | | | | | | | | | | | | | | | | | | | | | | | | | | | | | | | | | | | | | | | | | | | | | | | | | | | | | | | | | | | | | | | | | | | | | | |  |
| **Setting** | | | | | | | | | **Design/Method** | | | | | | | | | | | | | | | | | | | | | | | | | | | | | | | | | **Population** | | | | | | | | | | | | | | | | | | | | | | | | | | | | | | | | | | | | **Intervention** | | | | | | | | | | | | | | | | | | | | | | | | | | | | | | **Workforce Characteristics** | | | | | | | | | | | | | | | | | | | | | | | | | | | | | | | | | | | | | | | | | | | | | | | | | | | | | | | | | | | | | | | | | | | | | | | | | | | | | | | | | | | | | | | | | | | | | | | | | | | | | | | | | | | | | | | | | | | | | | | | | | | | | | | | | | | | | | | | | | | | | | | | | | | | | | | | | | | | | | | | | | | |  |
|  |  |  |  |  |  |  |  |  |  |  |  |  |  |  |  |  |  |  |  |  |  |  |  |  |  |  |  |  |  |  |  |  |  |  |  |  |  |  |  |  |  |  |  |  |  |  |  |  |  |  |  |  |  |  |  |  |  |  |  |  |  |  |  |  |  |  |  |  |  |  |  |  |  |  |  |  |  |  |  |  |  |  |  |  |  |  |  |  |  |  |  |  |  |  |  |  |  |  |  |  |  |  |  |  |  |  |  | **Cadres** | | | | | | | | | | | | | | | | | | | | | | | | | | | | | | | | | | | | | | **Description** | | | | | | | | | | | | | | | | | | | | | | | | | | | | | | | | **Training** | | | | | | | | | | | | | | | | | | | | | | | | | | | | | | | | | | | | | | | | **Supervision** | | | | | | | | | | | | | | | | | | | | | | | | | | | | | **Misc.** | | | | | | | | | | | | | | | | | | | | | | | | |  |
| 6 provinces in urban and rural South Africa | | | | | | | | | Qualitative. Participatory including focus groups (n=9), individual interviews (n=18) and transects walks (n=7) | | | | | | | | | | | | | | | | | | | | | | | | | | | | | | | | | 7-8 PWD or families of PWD in each province | | | | | | | | | | | | | | | | | | | | | | | | | | | | | | | | | | | | Physical and social rehabilitation by CRFs in disadvantaged communities in South Africa.  Approximately 200 CRFs working in 100 disadvantaged communities. | | | | | | | | | | | | | | | | | | | | | | | | | | | | | | Community rehabilitation facilitators (CRFs. | | | | | | | | | | | | | | | | | | | | | | | | | | | | | | | | | | | | | | Mid-level rehabilitation worker, working in communities to provide rehabilitation. Involved in physical and social rehabilitation, as well as advocacy, raising awareness of disability and lobbying for rights. | | | | | | | | | | | | | | | | | | | | | | | | | | | | | | | | Two year training | | | | | | | | | | | | | | | | | | | | | | | | | | | | | | | | | | | | | | | | Not discussed | | | | | | | | | | | | | | | | | | | | | | | | | | | | | **Quality: 3**  MMAT – Qualitative  Research question asks for ‘impact’ – which is ill defined for methods | | | | | | | | | | | | | | | | | | | | | | | | |  |
| **CMOCs** | | | | | | | | | | | | | | | | | | | | | | | | | | | | | | | | | | | | | | | | | | | | | | | | | | | | | | | | | | | | | | | | | | | | | | | | | | | | | | | | | | | | | | | | | | | | | | | | | | | | | | | | | | | | | | | | | | | | | | | | | | | | | | | | | | | | | | | | | | | | | | | | | | | | | | | | | | | | | | | | | | | | | | | | | | | | | | | | | | | | | | | | | | | | | | | | | | | | | | | | | | | | | | | | | | | | | | | | | | | | | | | | | | | | | | | | | | | | | | | | | | | | | | | | | | | | | | | | | | | | | | | | | | | | | | | |  |
| **Context** | | | | | | | | | | | | | | | | | | | | | | | | | | | | | | **Mechanisms** | | | | | | | | | | | | | | | | | | | | | | | | | | | | | | | | | | | | | | | | | | | | | | | | | | | | | | | | | | | | | | | | | | | | | | | | | **Outcomes** | | | | | | | | | | | | | | | | | | | | | | | | | | | | | | | | | | | | | | | | | | | | | | | | | | | | | | | | | | | | | | | | | | | | | | | | | | | **CMOCs** | | | | | | | | | | | | | | | | | | | | | | | | | | | | | | | | | | | | | | | | | | | | | | | | | | | | | | | | | | | | | | | | | | | | | | | | | | | | | | | | | | | | | | | | | | | | | |  |
| National Disability Strategy White paper, forming the basis of the national rehabilitation strategy.  CRF programme in South Africa is well established programme with mid-level CBR workers known as CRFs which function to directly provide rehabilitation to person and family and refer.  Individual level, in the main, via home visits, exercises and assistive devices. ). In South Africa approximately one quarter of CRFs are disabled persons or family members of disabled persons | | | | | | | | | | | | | | | | | | | | | | | | | | | | | | 1.A lack of clear job descriptions, and chain of service deliveries to PWD and their families may impact families’ opinion of their rehabilitation provider.  2. CRFs may address gaps in psychosocial support and counseling needs of PWDs – even when not within their mandate.  3. Interpersonal relationships and support by workers was highly valued with clients and this role can turn into providing additional support beyond the scope of physical rehabilitation.  4. Poor/ineffective supervision may result in lack of clear job description and professional mandate.  5. CRFs are seen as coming from the health facility and may be viewed as ‘health professionals’ according to PWD and not community issues  6. If CBR workers are too distant from the community itself, i.e., not from inside it, they may have a more difficult job with connecting at the local level | | | | | | | | | | | | | | | | | | | | | | | | | | | | | | | | | | | | | | | | | | | | | | | | | | | | | | | | | | | | | | | | | | | | | | | | | Participants identified most significant impact at the individual level, including; practical intervention like home visits, exercises and training on activities of daily living.  Advise and counseling by CRF and their approach to working with PWD was seen as significant and positive.  Counselling had impact on PWD self-awareness and assisted in building relationships between CRF and PWD.  Strong relationships provided motivation and encouragement.  Families identified that programme had gaps in service delivery – mostly identification of needs of PWD, social circumstances including community interventions. Though families were unsure if this work fell within the CRF remit of their work.  Identified issues with CRFs being distant from the community.  Gaps in service delivery were influenced by different factors in different provinces (for example, lack of transportation or poor supervision). | | | | | | | | | | | | | | | | | | | | | | | | | | | | | | | | | | | | | | | | | | | | | | | | | | | | | | | | | | | | | | | | | | | | | | | | | | | 1.Family members of persons with disabilities need to be involved in the process of CBR.  2. University training, by inculcating a culture of ‘professional,’ may actually put distance between CBR and communities, and to some extent thereby become counterproductive.  3.Being a “known member of their communities” for enabling maximal performance in the role.  4. Being perceived as both “available” and “approachable” and able to visit the PWD at home, seem crucial.  5. CBR workers need skills in social influence/advocacy to raise awareness and change negative attitudes towards PWD.  6. CBR workers should be multi-skilled in that they have training on helping with assistive devices, physical rehabilitation and exercises, counseling skills and stress reduction for PWD and their families.  7. CBR workers should have skills in working with groups, for example leading group therapy.  8. Clear job descriptions and ensuring rehabilitation workers and community members are knowledgeable of rehabilitation workers’ roles with appropriate expectations.  9. Lack of understanding of roles of rehabilitation worker may negatively influence supervision  10. Needs assessment needs to be conducted at local levels to identify potential gaps that can influence service delivery or rehabilitation workers’ ability to preform. | | | | | | | | | | | | | | | | | | | | | | | | | | | | | | | | | | | | | | | | | | | | | | | | | | | | | | | | | | | | | | | | | | | | | | | | | | | | | | | | | | | | | | | | | | | | | |  |
| **Title:** Community-based rehabilitation programme as a model for task-shifting | | | | | | | | | | | | | | | | | | | | | | | | | | | | | | | | | | | | | | | | | | | | | | | | | | | | | | | | | | | | | | | | | | | | | | | | | | | | | | | | | | | | | | | | | | | | | | | | | | | | | | | | | | | | | | | | | | | | | | | | | | | | | | | | | | | | | | | | | | | | | | | | | | | | | | | | | | | | | | | | | | | | | | | | | | | | | | | | | | | | | | | | | | | | | | | | | | | | | | | | | | | | | | | | | | | | | | | | | | | | | | | | | | | | | | | | | | | | | | | | | | | | | | | | | | | | | | | | | | | | | | | | | | | | | | | |  |
| **Authors:** Dawad, S. & Jobson, G. | | | | | | | | | | | | | | | | | | | | | | | | | | | | | | | | | | | | | | | | | | | | | | | | | | | | | | | | | | | | | | | | | | | | | | | | | | | | | | | | | | | | | | | | | | | | | | | | | | | | | | | | | | | | | | | | | | | | | | | | | | | | | | | | | | | | | | | | | | | | | | | | | | | | | | | | | | | | | | | | | | | | | | | | | | | | | | | | | | | | | | | | | | | | | | | | | | | | | | | | | | | | | | | | | | | | | | | | | | **Year:** 2011 | | | | | | | | | | | | | | | | | | | | | | | | | | | | | | | | | | | | | | | | | | | | | | | | | | | | | |  |
| **Summary:** Describes mid-level cadre (CRF) and rehabilitation team providing community rehabilitation for persons living with HIV and their families | | | | | | | | | | | | | | | | | | | | | | | | | | | | | | | | | | | | | | | | | | | | | | | | | | | | | | | | | | | | | | | | | | | | | | | | | | | | | | | | | | | | | | | | | | | | | | | | | | | | | | | | | | | | | | | | | | | | | | | | | | | | | | | | | | | | | | | | | | | | | | | | | | | | | | | | | | | | | | | | | | | | | | | | | | | | | | | | | | | | | | | | | | | | | | | | | | | | | | | | | | | | | | | | | | | | | | | | | | | | | | | | | | | | | | | | | | | | | | | | | | | | | | | | | | | | | | | | | | | | | | | | | | | | | | | |  |
| **Setting** | | | | | | | | | **Design/Methods** | | | | | | | | | | | | | | | | | **Population** | | | | | | | | | | | | | | | | | | | | | | | | | | | | | | | | | | | | | | **Intervention** | | | | | | | | | | | | | | | | | | | | | | | | | | | | | | | | | | | | | | | | | | | | | | | | | | | | | | | | | **Workforce Characteristics** | | | | | | | | | | | | | | | | | | | | | | | | | | | | | | | | | | | | | | | | | | | | | | | | | | | | | | | | | | | | | | | | | | | | | | | | | | | | | | | | | | | | | | | | | | | | | | | | | | | | | | | | | | | | | | | | | | | | | | | | | | | | | | | | | | | | | | | | | | | | | | | | | | | | | | |  |
|  | | | | | | |  | | | | | | | | | | | | | | | | |  | | | | | | | | | | | | | | | | | | | | | | | | | | | | | | | | | | | | |  | | | | | | | | | | | | | | | | | | | | | | | | | | | | | | | | | | | | | | | | | | | | | | | | | | | | | | | | | | | **Cadres** | | | | | | | | | | | | | | | | | | | | | | | | | | | | | | | | | **Description** | | | | | | | | | | | | | | | | | | | | | | | | | | | | **Training** | | | | | | | | | | | | | | | | | | | | | | | | | | | | | | | | | | | | | | | | | | | | | | | | | | | **Supervision** | | | | | | | | | | | | | | | | | | | | | | | | **Misc.** | | | | | | | | | | | | | | | |  |
| Rural Kaw-Zulu Natal, Eastern South Africa | | | | | | | Qualitative, semi-structured interviews, focus group. | | | | | | | | | | | | | | | | | Beneficiaries of CBR programme, home and community-based carers (HCBCs), NGO managers and CRFs. | | | | | | | | | | | | | | | | | | | | | | | | | | | | | | | | | | | | | Community-based rehabilitation provided by CRF workers, thorough an established NGO (HEARD). CRFs primary rehabilitation providers, working with HCBCs at the community level. CRFs work in team of rehabilitation workers including: speech therapist, occupational therapist, physiotherapists and psychologist. Community health workers and HCBCs refer PWD to CRFs. | | | | | | | | | | | | | | | | | | | | | | | | | | | | | | | | | | | | | | | | | | | | | | | | | | | | | | | | | | | Community rehabilitation facilitators (CRFs) | | | | | | | | | | | | | | | | | | | | | | | | | | | | | | | | | Primary providers of rehabilitation services in community, working in team with more specialized cadres. PWD referred by community health workers or home and community-based carers in community to CRFs. CRFs are multi-skilled workers | | | | | | | | | | | | | | | | | | | | | | | | | | | | 2 year training course provided by NGO with strong practical component. (class of 13 would work with 300 PWD over training). Course focused on rehabilitation and community development with broad range. | | | | | | | | | | | | | | | | | | | | | | | | | | | | | | | | | | | | | | | | | | | | | | | | | | | Not described, but services provided in a tiered system with more specialized cadres at facility level. | | | | | | | | | | | | | | | | | | | | | | | | 13 students worked with approximately 300 PWD over the 2-year training  **Quality: 3**  MMAT Qualitative | | | | | | | | | | | | | | | |  |
| **CMOCs** | | | | | | | | | | | | | | | | | | | | | | | | | | | | | | | | | | | | | | | | | | | | | | | | | | | | | | | | | | | | | | | | | | | | | | | | | | | | | | | | | | | | | | | | | | | | | | | | | | | | | | | | | | | | | | | | | | | | | | | | | | | | | | | | | | | | | | | | | | | | | | | | | | | | | | | | | | | | | | | | | | | | | | | | | | | | | | | | | | | | | | | | | | | | | | | | | | | | | | | | | | | | | | | | | | | | | | | | | | | | | | | | | | | | | | | | | | | | | | | | | | | | | | | | | | | | | | | | | | | | | | | | | | | | | | | |  |
| **Context** | | | | | | | | | | | | | | | | | | | | | | | | | | | | | | | | | | | | | | | | | | | | | | | | | | **Mechanisms** | | | | | | | | | | | | | | | | | | | | | | | | | | | | | | | | | | | | | | | | | | | | | | | | | | | | | | | | | | | | | | | | | | | | | | | | | | | | | | | **Outcomes** | | | | | | | | | | | | | | | | | | | | | | | | | | | | | | | | | | | | | | | | | | | | | | | | | | | | | | | | | **CMOCs** | | | | | | | | | | | | | | | | | | | | | | | | | | | | | | | | | | | | | | | | | | | | | | | | | | | | | | | | | | | | | | | | | | | | | | | | | | | | | | | | | | | | | |  |
| NGO implementing CBR programme has worked in area implementing various projects including health and infrastructure, with the first rehabilitation service (a support group) being offered in the early 1990s.  CBR programme established in 2000, with training young people as CRFs by a training organisation. Rehabilitation centre established, employing: rehabilitation coordinator, occupational therapist, speech and language therapist, assistant and eleven CRFs.  Teams conduct in-depth assessment and design programmes for CRFs to implement.  Household level service implemented by CRFs with more specialized service at NGO clinic. | | | | | | | | | | | | | | | | | | | | | | | | | | | | | | | | | | | | | | | | | | | | | | | | | | 1.Capacity of highly trained health workers to transfer skills to minimally trained primary health care workers.  2. Working in partnership with other lay health workers, but with clear roles – i.e. CRFs do rehabilitation.  3. NGOs strong track record and experience can lead community rehabilitation interventions operating in a tiered delivery model, from more general skills to more specialized in facilities.  4. Relationship and partnership between different health sector providers (HCBCs and CRFs, CRFs and NGO etc.), effective in providing rehabilitation service.  5. Referral system from primary health care community workers (CHWs and HCBCs) to rehabilitation team to identify PWD.  6. Assessment and specific rehabilitation programmes designed by more specialized services to be implemented by CRFs in community. | | | | | | | | | | | | | | | | | | | | | | | | | | | | | | | | | | | | | | | | | | | | | | | | | | | | | | | | | | | | | | | | | | | | | | | | | | | | | | | Developing support groups so that more students can be trained in CBR.  Facilitate self-reliance by imparting skills in PWD and their families.  Moving beyond palliative care to more educational support and basic medical care.  Beneficiaries of programme acknowledged benefits of programme participation including: physical rehab, education of families, counseling, and access to resources including accessing state increased community integration.  Scope of training lead to CRFs providing counseling to parents of PWD and PWD and also community education.  Decrease level of stigma noted by HCBCs towards PWD. | | | | | | | | | | | | | | | | | | | | | | | | | | | | | | | | | | | | | | | | | | | | | | | | | | | | | | | | | 1.Rehabilitation workers, especially those working in communities, require skill in basic counseling as they often provide emotional support to PWD and their families.  2.Training requires strong practical component, possibility with training-in-community, to best equip rehabilitation workers  3.Partnerships between one organization and another, e.g., NGO and government, can prevent PWD from being denied access  4.Rehabilitation workers should be knowledgeable and available to help PWD to access appropriate services including disability grants, medication and assistive devices.  5.Services offered in a team of rehabilitation workers, with general skills offered in the community and appropriate referral pathways for more specialized skills.  6.Rehabilitation services should be offered as a team approach  7.Rehabilitation in community can reduce stigma and negative attitudes towards PWD | | | | | | | | | | | | | | | | | | | | | | | | | | | | | | | | | | | | | | | | | | | | | | | | | | | | | | | | | | | | | | | | | | | | | | | | | | | | | | | | | | | | | |  |
| **Title:** “More of the same and try something new” Evaluation of the Community Based Rehabilitation Programme in Eritrea | | | | | | | | | | | | | | | | | | | | | | | | | | | | | | | | | | | | | | | | | | | | | | | | | | | | | | | | | | | | | | | | | | | | | | | | | | | | | | | | | | | | | | | | | | | | | | | | | | | | | | | | | | | | | | | | | | | | | | | | | | | | | | | | | | | | | | | | | | | | | | | | | | | | | | | | | | | | | | | | | | | | | | | | | | | | | | | | | | | | | | | | | | | | | | | | | | | | | | | | | | | | | | | | | | | | | | | | | | | | | | | | | | | | | | | | | | | | | | | | | | | | | | | | | | | | | | | | | | | | | | | | | | | | | | | |  |
| **Authors:** Grut, L., Hjort, P. & Eide, AH. | | | | | | | | | | | | | | | | | | | | | | | | | | | | | | | | | | | | | | | | | | | | | | | | | | | | | | | | | | | | | | | | | | | | | | | | | | | | | | | | | | | | | | | | | | | | | | | | | | | | | | | | | | | | | | | | | | | | | | | | | | | | | | | | | | | | | | | | | | | | | | | | | | | | | | | | | | | | | | | | | | | | | | | | | | | | | | | | | | | | | | | | | | | | | | | | | | | | | | | | | | | | | | | | | | | | | | | | **Year:** 2004 | | | | | | | | | | | | | | | | | | | | | | | | | | | | | | | | | | | | | | | | | | | | | | | | | | | | | | | |  |
| **Summary:** Evaluation of decentralized CBR programme in Eritrea organized by Ministry of Labour and Human Welfare using lay-health workers for implementation | | | | | | | | | | | | | | | | | | | | | | | | | | | | | | | | | | | | | | | | | | | | | | | | | | | | | | | | | | | | | | | | | | | | | | | | | | | | | | | | | | | | | | | | | | | | | | | | | | | | | | | | | | | | | | | | | | | | | | | | | | | | | | | | | | | | | | | | | | | | | | | | | | | | | | | | | | | | | | | | | | | | | | | | | | | | | | | | | | | | | | | | | | | | | | | | | | | | | | | | | | | | | | | | | | | | | | | | | | | | | | | | | | | | | | | | | | | | | | | | | | | | | | | | | | | | | | | | | | | | | | | | | | | | | | | |  |
| **Setting** | | | | **Design/**  **Method** | | | | | | | | | | | | | | | **Population** | | | | | | | | | | | | | | | | | | | | | | | | | | | | | | | | | | | | | | | | | | | | **Intervention** | | | | | | | | | | | | | | | | | | | | | | | | | | | | | | | | | | | | | | | | | | | | | | | | | | **Workforce Characteristics** | | | | | | | | | | | | | | | | | | | | | | | | | | | | | | | | | | | | | | | | | | | | | | | | | | | | | | | | | | | | | | | | | | | | | | | | | | | | | | | | | | | | | | | | | | | | | | | | | | | | | | | | | | | | | | | | | | | | | | | | | | | | | | | | | | | | | | | | | | | | | | | | | | | | | | | | | | | | | | |  |
|  | | | |  | | | | | | | | | | | | | | |  | | | | | | | | | | | | | | | | | | | | | | | | | | | | | | | | | | | | | | | | | |  | | | | | | | | | | | | | | | | | | | | | | | | | | | | | | | | | | | | | | | | | | | | | | | | | | | | **Cadres** | | | | | | | | | | | | | | | | | | | | | | | | | | **Description** | | | | | | | | | | | | | | | | | | | | | | | | | | | | | | | | | | | | | | | | | | | | | | | | | | | | | | | | | | | | | | | | | | **Training** | | | | | | | | | | | | | | | | | | | | | | | | | | | | | | | | | | | | | **Supervision** | | | | | | | | | | | | | | | | | | | | **Misc.** | | | | | | | | | |  |
| 16 villages in 4 sub-regions of Eritrea. | | | | Qualitative. Observation, interviews and focus groups.  77 interviews in total. | | | | | | | | | | | | | | | PWD (n=28), local supervisors (n=30), village administration (n=9) MLHW sub-regional heads (n=3), MLHW regional leaders/directors, (n=4), hospital staff (n=2), orthopaedic workshop (n=1) | | | | | | | | | | | | | | | | | | | | | | | | | | | | | | | | | | | | | | | | | | Decentralized programme run by Ministry of Labour and Human Welfare (MLHW). MoH provide hospital services with MLHW providing (2) orthopeadic clinics. Implementation of CBR at regional level, with LS living in village and responsible for daily follow-up of PWD. Village rehabilitation committees established. | | | | | | | | | | | | | | | | | | | | | | | | | | | | | | | | | | | | | | | | | | | | | | | | | | | | Local supervisors (LS) | | | | | | | | | | | | | | | | | | | | | | | | | | Village rehabilitation committees, local supervisors from the community. LS provide training, assistance and referrals for PWD in their communities.  Voluntary and non-paid.  Committees central to raising awareness and supporting LS. Communities suggest LS, and then community elects LS. Work according to CBR manual, make referrals, and focus on self-care, daily-living activities, and mobility. Work with physical and mental disabilities. | | | | | | | | | | | | | | | | | | | | | | | | | | | | | | | | | | | | | | | | | | | | | | | | | | | | | | | | | | | | | | | | | | 6-week training course for local supervisors, with yearly refresher courses. Including knowledge on community mobilization and knowledge on different kinds of disability. | | | | | | | | | | | | | | | | | | | | | | | | | | | | | | | | | | | | | Regular (every 2 months) sub-regional meeting with LS and staff from MLHW. | | | | | | | | | | | | | | | | | | | | Elder people and/or PWD encouraged to become LS.  **Quality: 3**  MMAT - Qualitative | | | | | | | | | |  |
| **CMOCs** | | | | | | | | | | | | | | | | | | | | | | | | | | | | | | | | | | | | | | | | | | | | | | | | | | | | | | | | | | | | | | | | | | | | | | | | | | | | | | | | | | | | | | | | | | | | | | | | | | | | | | | | | | | | | | | | | | | | | | | | | | | | | | | | | | | | | | | | | | | | | | | | | | | | | | | | | | | | | | | | | | | | | | | | | | | | | | | | | | | | | | | | | | | | | | | | | | | | | | | | | | | | | | | | | | | | | | | | | | | | | | | | | | | | | | | | | | | | | | | | | | | | | | | | | | | | | | | | | | | | | | | | | | | | | | | |  |
| **Context** | | | | | | | | | | | | | | | | | | | | | | | | | | | | | | | | | | | | | | | | | | | | | | | | | | | | | | | | | | | | | | | | | | | | | | | | | | | | | | | | | | | **Mechanisms** | | | | | | | | | | | | | | | | | | | | | | | | | | | | | | | | | | | | | | | | | | | | | | | | | | | | | | | | | | | | | | | | | | | | | | | | | | | | | | | | **Outcomes** | | | | | | | | | | | | | | | | | | | | | | | | | | | | | | | | | | | | | | | | | | | | | | | | | | | | | | | | | | | **CMOCs** | | | | | | | | | | | | | | | | | | | | | | | | | | | | | | | | | | | | | | | | | | | | | | | | | |  |
| CBR programme began as pilot in 1995, by 2004 covered 40% of the country with Ministry of Labour and Human Welfare as implementing agent and NAD (Norwegian Association of Disabled) as external contributor.  Approximately 25% of LS are female.  MLHW work in conjunction with other Ministries, including Ministry of Health and Ministry of Education.  Programme has LS survey all households in their village to identify PWD.  LS provide support for mental, physical and communication disabilities.  Village committee meetings held 2-3 times/year and important gatherings for LS to advocate for PWD.  Local Supervisors visit PWDs and families , assess and register problems and needs, working from a field manual.  Make referrals to hospitals and/or orthopaedic workshops.  Writing letters of support for referrals. Identifying income-generating opportunities.  Primary focus on the individual and his or her family at home in the household.  LS mostly around identification, assessment, referral, social support and advocacy | | | | | | | | | | | | | | | | | | | | | | | | | | | | | | | | | | | | | | | | | | | | | | | | | | | | | | | | | | | | | | | | | | | | | | | | | | | | | | | | | | | 1. “Inactive rehabilitation” (advocacy, referral etc.) regarded as highly important for rehabilitation programmes, however the “active rehabilitation skills” cannot be neglected.  2. Community identification and selection of rehabilitation workers may increase their acceptability, which can influence reception of messages especially around stigma and awareness raising.  3. Expectations of CBR workers and programmes should be made clear to community, and equally important is the ability for the managing department to adhere to their commitments as to not negatively reflect on the programme and the workers themselves.  4. The establishment of rehabilitation committees to help support LS and the programme implementation, as well as the social aspects of rehabilitation, important for community-based programmes.  5. Minimally trained lay health workers’ skills/competencies may have variation resulting in inconsistent services. | | | | | | | | | | | | | | | | | | | | | | | | | | | | | | | | | | | | | | | | | | | | | | | | | | | | | | | | | | | | | | | | | | | | | | | | | | | | | | | | Potential issues of identifying all PWD in village.  Lack of resources, especially for LS, including transport allowance.  Lack of competence in rehabilitation skills hindrance for more effective services.  Strong advocacy for rights of PWD through all levels of rehabilitation.  Increased access and opportunities for PWD including school.  Decreased stigma towards PWD and increased mobilization.  Increased expectations of CBR and CBR worker in community – problems with resources and also skill set of worker  Challenge of meeting commitment to LS, like small budget for transport  Lack of training on and active efforts to improve functional ability.  Variation in personal /individual competencies among the LS. | | | | | | | | | | | | | | | | | | | | | | | | | | | | | | | | | | | | | | | | | | | | | | | | | | | | | | | | | | | 1. Integration of CBR into MLHW can assist in stability, equality and sustainability of programmes; however, lack of resources at this level may influence success.  2.In contexts with very limited more specialized cadres of rehabilitation workers, CBR workers need to have strong skills in physical rehabilitation to improve functional ability – not enough to just assess, refer and provide “inactive rehabilitation”  3. A lack of specialized services to refer to can impact on the perceptions of CBR by villagers and PWD  4.The Local Supervisors themselves reported that their most important task, and thereby competency, was advocacy, especially during community gatherings.  5. Short training of lay workers may not be sufficient to provide appropriate rehabilitation interventions in the community | | | | | | | | | | | | | | | | | | | | | | | | | | | | | | | | | | | | | | | | | | | | | | | | | |  |
| **Title:** The Community Based Rehabilitation Programme of the University of the Philippines Manila, College of Allied Medical Professionals | | | | | | | | | | | | | | | | | | | | | | | | | | | | | | | | | | | | | | | | | | | | | | | | | | | | | | | | | | | | | | | | | | | | | | | | | | | | | | | | | | | | | | | | | | | | | | | | | | | | | | | | | | | | | | | | | | | | | | | | | | | | | | | | | | | | | | | | | | | | | | | | | | | | | | | | | | | | | | | | | | | | | | | | | | | | | | | | | | | | | | | | | | | | | | | | | | | | | | | | | | | | | | | | | | | | | | | | | | | | | | | | | | | | | | | | | | | | | | | | | | | | | | | | | | | | | | | | | | | | | | | | | | | | | | | |  |
| **Authors:** Magallona, MLM. & Datangel, JP. | | | | | | | | | | | | | | | | | | | | | | | | | | | | | | | | | | | | | | | | | | | | | | | | | | | | | | | | | | | | | | | | | | | | | | | | | | | | | | | | | | | | | | | | | | | | | | | | | | | | | | | | | | | | | | | | | | | | | | | | | | | | | | | | | | | | | | | | | | | | | | | | | | | | | | | | | | | | | | | | | | | | | | | | | | | | | | | | | | | | | | | | | | | | | | | | | | | | | | | | | | | | | | | | | | | | | **Year:** 2011 | | | | | | | | | | | | | | | | | | | | | | | | | | | | | | | | | | | | | | | | | | | | | | | | | | | | | | | | | | |  |
| **Summary:** Impact of community based rehabilitation trans-disciplinary training programme and service delivery. | | | | | | | | | | | | | | | | | | | | | | | | | | | | | | | | | | | | | | | | | | | | | | | | | | | | | | | | | | | | | | | | | | | | | | | | | | | | | | | | | | | | | | | | | | | | | | | | | | | | | | | | | | | | | | | | | | | | | | | | | | | | | | | | | | | | | | | | | | | | | | | | | | | | | | | | | | | | | | | | | | | | | | | | | | | | | | | | | | | | | | | | | | | | | | | | | | | | | | | | | | | | | | | | | | | | | | | | | | | | | | | | | | | | | | | | | | | | | | | | | | | | | | | | | | | | | | | | | | | | | | | | | | | | | | | |  |
| **Setting** | | | | | **Design/Method** | | | | | | | | | | | | | | | | | | | | | | | | | | | | | | | | | | **Population** | | | | | | | | | | | | | | | | | | | | | | | | | | | | | | | | | | | | **Intervention** | | | | | | | | | | | | | | | | | | | | | | | | | | | | | | | | | | | | | | | **Workforce Characteristics** | | | | | | | | | | | | | | | | | | | | | | | | | | | | | | | | | | | | | | | | | | | | | | | | | | | | | | | | | | | | | | | | | | | | | | | | | | | | | | | | | | | | | | | | | | | | | | | | | | | | | | | | | | | | | | | | | | | | | | | | | | | | | | | | | | | | | | | | | | | | | | | | | | | | | | | | | | | | | |  |
|  |  |  |  |  |  |  |  |  |  |  |  |  |  |  |  |  |  |  |  |  |  |  |  |  |  |  |  |  |  |  |  |  |  |  |  |  |  |  |  |  |  |  |  |  |  |  |  |  |  |  |  |  |  |  |  |  |  |  |  |  |  |  |  |  |  |  |  |  |  |  |  |  |  |  |  |  |  |  |  |  |  |  |  |  |  |  |  |  |  |  |  |  |  |  |  |  |  |  |  |  |  |  |  |  |  |  |  |  |  |  |  |  |  | **Cadres** | | | | | | | | | | | | | | | | | | | | | | | | | | | | | | | | | | | | **Description** | | | | | | | | | | | | | | | | | | | | | | | | | | | | | | | | | | **Training** | | | | | | | | | | | | | | | | | | | | | | | | | | | | | | | | | | | | | | | | | | **Supervision** | | | | | | | | | | | | | | | | | | | | | | | | | | | | | | | | **Misc.** | | | | | | | | | | | | | |  |
| Rural, municipality of Rodriguez, Rizal | | | | | Mixed methods. Questionnaires, interviews, focus groups and review of secondary data. | | | | | | | | | | | | | | | | | | | | | | | | | | | | | | | | | | Students and alumni of training programme, PWD, CBR workers, local leaders and stakeholders from agencies involved with training programme. | | | | | | | | | | | | | | | | | | | | | | | | | | | | | | | | | | | | CBR trans-disciplinary training programme run by College of Allied Medical Professions of the University of the Philippines Manila (UMP-CAMP) as part of UP-Comprehensive Community Health Programme (UP-CCHP), with then implementation of CBR programme. | | | | | | | | | | | | | | | | | | | | | | | | | | | | | | | | | | | | | | | Community based rehabilitation workers and interns. | | | | | | | | | | | | | | | | | | | | | | | | | | | | | | | | | | | | Interns trained CBR workers, who work in a team approach. Training designed and lead by academic institution, (UMP-CAMP) and involved multiple cadres of rehabilitation professionals. Undergraduate students as part of allied health professional group. | | | | | | | | | | | | | | | | | | | | | | | | | | | | | | | | | | Trans-disciplinary approach, practical skills in the community. | | | | | | | | | | | | | | | | | | | | | | | | | | | | | | | | | | | | | | | | | | Once per week supervision by clinical supervisors.  Supervision in multiple forms – from clinical, community, CBR workers and officials. | | | | | | | | | | | | | | | | | | | | | | | | | | | | | | | | **Quality: 2**  MMAT: MM | | | | | | | | | | | | | |  |
| **CMOCs** | | | | | | | | | | | | | | | | | | | | | | | | | | | | | | | | | | | | | | | | | | | | | | | | | | | | | | | | | | | | | | | | | | | | | | | | | | | | | | | | | | | | | | | | | | | | | | | | | | | | | | | | | | | | | | | | | | | | | | | | | | | | | | | | | | | | | | | | | | | | | | | | | | | | | | | | | | | | | | | | | | | | | | | | | | | | | | | | | | | | | | | | | | | | | | | | | | | | | | | | | | | | | | | | | | | | | | | | | | | | | | | | | | | | | | | | | | | | | | | | | | | | | | | | | | | | | | | | | | | | | | | | | | | | | | | |  |
| **Context** | | | | | | | | | | | | | | | | | | | | | | | | | | | | **Mechanisms** | | | | | | | | | | | | | | | | | | | | | | | | | | | | | | | | | | | | | | | | | | | | | | | | | | | | | | | | | | | | | | | | | | | | | | | | | | **Outcomes** | | | | | | | | | | | | | | | | | | | | | | | | | | | | | | | | | | | | | | | | | | | | | | | | | | | | | | | | | | | | | | | | | | | | | | | | | | | | | | | | | | | | | | | | | | | | | **CMOCs** | | | | | | | | | | | | | | | | | | | | | | | | | | | | | | | | | | | | | | | | | | | | | | | | | | | | | | | | | | | | | | | | | | | | | | | | | | | | |  |
| Training programme run in 1989 by UP CAMP with CBR programme running. UP CAMP has been working in CBR since 1974.  Training of UPM-CAMP students in CBR using a Trans-disciplinary Approach, including the design, development and running of the programme.  Training had the ‘interns’ living and working in the community for two months.  Once per week supervision by clinical supervisors for training.  Supervision in multiple forms – from clinical, community, CBR workers and officials. | | | | | | | | | | | | | | | | | | | | | | | | | | | | 1. Working as a team to train, supervise and implement CBR from various levels, including academic.  2. Lack of time CBR workers have for their own families or other commitment is a demotivating factor.  3. Training with a strong practical component (fieldwork) highly accepted and also may contribute to more positive attitudes and perceptions of workers to PWD.  4. Supervision from all levels (community, institution, health facility) – with 1/week with clinical supervisors.  5. Integrating CBR learning into an academic institution while providing opportunities for practical learning.  6. Better understanding and able to related by having either living with community | | | | | | | | | | | | | | | | | | | | | | | | | | | | | | | | | | | | | | | | | | | | | | | | | | | | | | | | | | | | | | | | | | | | | | | | | | Perspectives and attitudes of PTs, OTs and SPs towards CBR improved significantly, especially on community involvement.  “Interns” expressed need for more fieldwork as opposed to theory for training.  Some being trained were satisfied with level of supervision, though some requested more frequent.  CBR workers valued leaders who: knowledgeable, good interpersonal relationship, allows for participation, transparent, accountable, sharing and good listener.  Demotivating factors:  Some CBR workers expressed not having time for family or other activities/jobs.  Demotivating factors:  When officials failed to attend required clinics, or to meet their financial obligations.  Identified leadership traits: supportive; proper training sessions; sincerity; coordination; relationships.  Majority of CBR workers confident in their skills.  CBR workers wanted more publicity of their work to that the community is aware of their skills. | | | | | | | | | | | | | | | | | | | | | | | | | | | | | | | | | | | | | | | | | | | | | | | | | | | | | | | | | | | | | | | | | | | | | | | | | | | | | | | | | | | | | | | | | | | | | 1.Integrating CBR learning into an academic institution while providing opportunities for practical learning.  2. Undergraduate students studying community health programmes as part of allied health professional study programme.  3.Trans-disciplinary training to provide comprehensive understanding and support, while increasing positive attitudes towards PWD.  4. Extended field placements during training may increase positive attitudes towards and understanding of PWD.  5. Team approach to training, supervision and rehabilitation with clear links to specialized clinical skills.  6. Specialized processionals as supervisors and frequent supervision.  7. Academic bodies can have key role in designing and running training programmes for rehabilitation if they are able to use their allied health networks.  8. Rehabilitation workers able to recommend skills they require from a training programme and supervisor, which can make services more acceptable if followed. | | | | | | | | | | | | | | | | | | | | | | | | | | | | | | | | | | | | | | | | | | | | | | | | | | | | | | | | | | | | | | | | | | | | | | | | | | | | |  |
| **Title:** Building capacity in mental health interventions in low resource countries: an apprenticeship model for training local providers | | | | | | | | | | | | | | | | | | | | | | | | | | | | | | | | | | | | | | | | | | | | | | | | | | | | | | | | | | | | | | | | | | | | | | | | | | | | | | | | | | | | | | | | | | | | | | | | | | | | | | | | | | | | | | | | | | | | | | | | | | | | | | | | | | | | | | | | | | | | | | | | | | | | | | | | | | | | | | | | | | | | | | | | | | | | | | | | | | | | | | | | | | | | | | | | | | | | | | | | | | | | | | | | | | | | | | | | | | | | | | | | | | | | | | | | | | | | | | | | | | | | | | | | | | | | | | | | | | | | | | | | | | | | | | | |  |
| **Authors:** Murray, LK., Dorsey, S., Bolton, P., Jordans, MJD., Rahman, A., Bass, J. & Verdeli, H. | | | | | | | | | | | | | | | | | | | | | | | | | | | | | | | | | | | | | | | | | | | | | | | | | | | | | | | | | | | | | | | | | | | | | | | | | | | | | | | | | | | | | | | | | | | | | | | | | | | | | | | | | | | | | | | | | | | | | | | | | | | | | | | | | | | | | | | | | | | | | | | | | | | | | | | | | | | | | | | | | | | | | | | | | | | | | | | | | | | | | | | | | | | | | | | | | | | | | | | | | | | | | | | | | | | | | **Year:** 2011 | | | | | | | | | | | | | | | | | | | | | | | | | | | | | | | | | | | | | | | | | | | | | | | | | | | | | | | | | | |  |
| **Summary:** Combination of training and supervision methods discussed across 12 projects in different countries, to provide guidelines for lay counselor training and supervision in mental health counseling interventions. | | | | | | | | | | | | | | | | | | | | | | | | | | | | | | | | | | | | | | | | | | | | | | | | | | | | | | | | | | | | | | | | | | | | | | | | | | | | | | | | | | | | | | | | | | | | | | | | | | | | | | | | | | | | | | | | | | | | | | | | | | | | | | | | | | | | | | | | | | | | | | | | | | | | | | | | | | | | | | | | | | | | | | | | | | | | | | | | | | | | | | | | | | | | | | | | | | | | | | | | | | | | | | | | | | | | | | | | | | | | | | | | | | | | | | | | | | | | | | | | | | | | | | | | | | | | | | | | | | | | | | | | | | | | | | | |  |
| **Setting** | | | | | | | | | | | | | | | | | | **Design/**  **Methods** | | | | | | | | | | | | | | | | | | | | | | | | | | | **Population** | | | | | | | | | | | | | | | | | | | | | | | | | | | | | | | | | | | | **Intervention** | | | | | | | | | | | | | | | | | | | | | | | | **Workforce Characteristics** | | | | | | | | | | | | | | | | | | | | | | | | | | | | | | | | | | | | | | | | | | | | | | | | | | | | | | | | | | | | | | | | | | | | | | | | | | | | | | | | | | | | | | | | | | | | | | | | | | | | | | | | | | | | | | | | | | | | | | | | | | | | | | | | | | | | | | | | | | | | | | | | | | | | | | | | | | | | | | | | | | | | | | |  |
|  |  |  |  |  |  |  |  |  |  |  |  |  |  |  |  |  |  |  |  |  |  |  |  |  |  |  |  |  |  |  |  |  |  |  |  |  |  |  |  |  |  |  |  |  |  |  |  |  |  |  |  |  |  |  |  |  |  |  |  |  |  |  |  |  |  |  |  |  |  |  |  |  |  |  |  |  |  |  |  |  |  |  |  |  |  |  |  |  |  |  |  |  |  |  |  |  |  |  |  |  |  |  |  |  | **Cadres** | | | | | | | | | | | | | | | | | | | | | | | | | | | | | **Description** | | | | | | | | | | | | | | | | | | | | | | | | | | | | | | | | | | | | | | | | | **Training** | | | | | | | | | | | | | | | | | | | | | | | | | | | | | | | | | | | | | | **Supervision** | | | | | | | | | | | | | | | | | | | | | | | | | | | | | | | | | | | | | | | | | | | | | | | | | **Misc.** | | | | | | | | | |  |
| Sri Lanka, Burundi, Indonesia, Sudan, Cambodia, Uganda, Zambia, Tanzania, Pakistan, Iraq, Nepal and Thailand. | | | | | | | | | | | | | | | | | | Case Study across 12 countries. | | | | | | | | | | | | | | | | | | | | | | | | | | | Lay health workers trained for persons with mental disabilities. | | | | | | | | | | | | | | | | | | | | | | | | | | | | | | | | | | | | Lay health worker counseling interventions and their training and supervision models. | | | | | | | | | | | | | | | | | | | | | | | | Lay health workers (LHW) | | | | | | | | | | | | | | | | | | | | | | | | | | | | | Requirement and skills vary between countries, but majority are: minimum high school education, strong interpersonal skills, desire to assist persons with disabilities and selected by communities in which they work. Majority (but not all) are voluntary, and partners dependent on context. | | | | | | | | | | | | | | | | | | | | | | | | | | | | | | | | | | | | | | | | | Interactive and practical, with approximately 1:2 time spend on lecture style vs. practical learning or skill practice | | | | | | | | | | | | | | | | | | | | | | | | | | | | | | | | | | | | | | Supervisors also in referral chain and function as a tier system between LHW and more specialized services. Supervision used as form of post-training, with it being ongoing and supportive. Practice groups developed. | | | | | | | | | | | | | | | | | | | | | | | | | | | | | | | | | | | | | | | | | | | | | | | | | **Quality: NA** Descriptive Case Study | | | | | | | | | |  |
| **CMOCs** | | | | | | | | | | | | | | | | | | | | | | | | | | | | | | | | | | | | | | | | | | | | | | | | | | | | | | | | | | | | | | | | | | | | | | | | | | | | | | | | | | | | | | | | | | | | | | | | | | | | | | | | | | | | | | | | | | | | | | | | | | | | | | | | | | | | | | | | | | | | | | | | | | | | | | | | | | | | | | | | | | | | | | | | | | | | | | | | | | | | | | | | | | | | | | | | | | | | | | | | | | | | | | | | | | | | | | | | | | | | | | | | | | | | | | | | | | | | | | | | | | | | | | | | | | | | | | | | | | | | | | | | | | | | | | | |  |
| **Context** | | | | | | | | | | | | | | | | | | | | | | | | | | | | | | | | | | | | | | | | | | | | | | | | | | | | | | | | **Mechanisms** | | | | | | | | | | | | | | | | | | | | | | | | | | | | | | | | | | | | | | | | | | | | | | | | | | | | | | | | | | | | | | | | | | | | | | | **Outcomes** | | | | | | | | | | | | | | | | | | | | | | | | | | | | | | | | | | | | | | | | | | | | | | | | | | | | | | | | | | | | | | | | | | | | | | | | | | | | | | | | **CMOCs** | | | | | | | | | | | | | | | | | | | | | | | | | | | | | | | | | | | | | | | | | | | | | | | | | | | | | | | | | | | | | | | | |  |
| Three tier systems of counselors, supervisors and trainers, which also act as the referral chain.  Trainers – experts in mental health intervention, usually from outside the project area  Supervisors – ideally local and chosen for more advanced role  Counselors – local individuals who provide the intervention.  Interactive and practical teaching combined with classwork, including piloting and then feedback.  Counselors and supervisors should be selected from community and affiliated with local partnership organizations.  Beneficial if supervisors have some teaching provision of services.  Supervisors specifically trained for intervention and then ability to supervise.  Supervisors and trainers work together to assess the ability of the counselors. Also work with counselors to develop plan for implementation. | | | | | | | | | | | | | | | | | | | | | | | | | | | | | | | | | | | | | | | | | | | | | | | | | | | | | | | | 1.Strong supportive supervision, with decision-making interaction between counselors and supervisors, to provide context appropriate services.  2.Three-tier human resource model for community rehabilitation increases the amount of health staff available in contexts with little specialized capacity: counselor (community trained provider of intervention), supervisor (more experience than counselor with extra supervision training but also trained on intervention), and trainer (experts in the field who train supervisors/counselors).  3.Continual training and support mechanisms for both counselors and *supervisors*, including group sessions, case overview and practical supervision/training.  4. Learning ‘how’ to supervise is seen as key for sustainability of service and autonomous workforce development and capacity building.  5. Use of experiential and practical training methods and learning strategies. | | | | | | | | | | | | | | | | | | | | | | | | | | | | | | | | | | | | | | | | | | | | | | | | | | | | | | | | | | | | | | | | | | | | | | | Cross-cultural nature of intervention and human resources should be considered throughout all steps.  Supervisors monitor counselors’ fidelity by self-report and observation of sessions. Encouraging counselors to ‘objective’ report on counseling.  If no supervisors with teaching experience available, often counselors that are exceling in training will be chosen.  Supervisors continue to have training and support mechanisms including supervisor groups, close supervision of limited cases by local supervisor and trainer consultation.  Supervisors and counselors work together on decisions of ‘flexibility and fidelity’ for the delivery of the intervention – balancing the core components of the intervention with the flexibility and the adaptation fit to the population.  Challenges:  Issues of supervisor and counselor attrition, limited ‘experienced’ capacity for handling clinical emergencies, time required, need for supervisors and trainers to speak same language. | | | | | | | | | | | | | | | | | | | | | | | | | | | | | | | | | | | | | | | | | | | | | | | | | | | | | | | | | | | | | | | | | | | | | | | | | | | | | | | | 1.Supervision is key to the success of lay health worker programmes, and needs to be given of attention and resources.  2.Traditional supervision structures need to be enhanced, and include more group discussion and support, field-based, and experimental methods that fix within the local context.  3. Protect against subsequent attrition by creating support, monetary compensation where needed, adequate time allowances, and opportunities for career advancement, be prepared for counselors to move up to supervisors at all stages.  4.Supervision capacity is key to supply and distribution, via maintenance and support (versus loss and turnover), explicitly mentioned local supervisors rather than reliance on locally-based expatriates.  5. Lay health worker programmes delivered in a tier system from counselor, supervisor to more specialized service can provide rehabilitation to more vulnerable populations or be effective when human resources are limited. | | | | | | | | | | | | | | | | | | | | | | | | | | | | | | | | | | | | | | | | | | | | | | | | | | | | | | | | | | | | | | | | |  |
| **Title:** An Inter-country Study of Expectations Roles, Attitudes and Behaviours of Community-based Rehabilitation Volunteers | | | | | | | | | | | | | | | | | | | | | | | | | | | | | | | | | | | | | | | | | | | | | | | | | | | | | | | | | | | | | | | | | | | | | | | | | | | | | | | | | | | | | | | | | | | | | | | | | | | | | | | | | | | | | | | | | | | | | | | | | | | | | | | | | | | | | | | | | | | | | | | | | | | | | | | | | | | | | | | | | | | | | | | | | | | | | | | | | | | | | | | | | | | | | | | | | | | | | | | | | | | | | | | | | | | | | | | | | | | | | | | | | | | | | | | | | | | | | | | | | | | | | | | | | | | | | | | | | | | | | | | | | | | | | | | |  |
| **Authors:** Sharma, M. & Deepak, S. | | | | | | | | | | | | | | | | | | | | | | | | | | | | | | | | | | | | | | | | | | | | | | | | | | | | | | | | | | | | | | | | | | | | | | | | | | | | | | | | | | | | | | | | | | | | | | | | | | | | | | | | | | | | | | | | | | | | | | | | | | | | | | | | | | | | | | | | | | | | | | | | | | | | | | | | | | | | | | | | | | | | | | | | | | | | | | | | | | | | | | | | | | | | | | | | | | | | | | | | | | | | | | | | | | | | | **Year:** 2003 | | | | | | | | | | | | | | | | | | | | | | | | | | | | | | | | | | | | | | | | | | | | | | | | | | | | | | | | | | |  |
| **Summary:** Survey of lay community rehabilitation workers across 7 countries to investigate job satisfaction | | | | | | | | | | | | | | | | | | | | | | | | | | | | | | | | | | | | | | | | | | | | | | | | | | | | | | | | | | | | | | | | | | | | | | | | | | | | | | | | | | | | | | | | | | | | | | | | | | | | | | | | | | | | | | | | | | | | | | | | | | | | | | | | | | | | | | | | | | | | | | | | | | | | | | | | | | | | | | | | | | | | | | | | | | | | | | | | | | | | | | | | | | | | | | | | | | | | | | | | | | | | | | | | | | | | | | | | | | | | | | | | | | | | | | | | | | | | | | | | | | | | | | | | | | | | | | | | | | | | | | | | | | | | | | | |  |
| **Setting** | | | | | | | | | | | | | | **Design/Methods** | | | | | | | | | | | | | | | | | | | | | | | | | | | | | | | | | | | | **Population** | | | | | | | | | | | | | | | | | | | | | | | | | | | | | | | **Intervention** | | | | | | | | | | | | | | | | | | | | | **Workforce Characteristics** | | | | | | | | | | | | | | | | | | | | | | | | | | | | | | | | | | | | | | | | | | | | | | | | | | | | | | | | | | | | | | | | | | | | | | | | | | | | | | | | | | | | | | | | | | | | | | | | | | | | | | | | | | | | | | | | | | | | | | | | | | | | | | | | | | | | | | | | | | | | | | | | | | | | | | | | | | | | | | | | | | | | | | | | | |  |
|  |  |  |  |  |  |  |  |  |  |  |  |  |  |  |  |  |  |  |  |  |  |  |  |  |  |  |  |  |  |  |  |  |  |  |  |  |  |  |  |  |  |  |  |  |  |  |  |  |  |  |  |  |  |  |  |  |  |  |  |  |  |  |  |  |  |  |  |  |  |  |  |  |  |  |  |  |  |  |  |  |  |  |  |  |  |  |  |  |  |  |  |  |  |  |  |  |  |  |  |  |  | **Cadres** | | | | | | | | | | | | | | | | | | | | | | | | | | | | | | | | | | | | | | | | | | | | | **Description** | | | | | | | | | | | | | | | | | | | | | | | | | | | | | | | | | | | | | | | | | | | | | | | | | | | | | | | | | | | | | | | | | | | | | | | | | | | | **Training** | | | | | | | | | | | | | | | | | | | | | **Supervision** | | | | | | | | | | | | | | | | | | | | | | | | **Misc.** | | | |  |
| Eritrea, Egypt, India, Mongolia, Papua New Guinea, Pakistan, Vietnam. | | | | | | | | | | | | | | Quantitative, cross-sectional design, correlational only. 60 item questionnaire | | | | | | | | | | | | | | | | | | | | | | | | | | | | | | | | | | | | 176 Community based rehabilitation workers | | | | | | | | | | | | | | | | | | | | | | | | | | | | | | | Providing community based services with connection to NGO Associazione Italian Amici di Raoul Follereau (AIFO) | | | | | | | | | | | | | | | | | | | | | Community based rehabilitation volunteers | | | | | | | | | | | | | | | | | | | | | | | | | | | | | | | | | | | | | | | | | | | | | Volunteers defined as: local residents; involved in some CBR work; not employed regularly by organisation; payment either as token or not as regular duty.  Demographics:  - 74% education less than highschool  - 25.7% former CBR workers  - 83.7% non-disabled  - 57% no family member living with disability  - 56% no monetary compensation; 25.9% stipend; 18.5% other  - reimbursement: none 18.5%; travel and/or meals 63.0% | | | | | | | | | | | | | | | | | | | | | | | | | | | | | | | | | | | | | | | | | | | | | | | | | | | | | | | | | | | | | | | | | | | | | | | | | | | | Varying depending on context. | | | | | | | | | | | | | | | | | | | | | Not reported. | | | | | | | | | | | | | | | | | | | | | | | | **Quality:3**  MMAT:Quant4 | | | |  |
| **CMOCs** | | | | | | | | | | | | | | | | | | | | | | | | | | | | | | | | | | | | | | | | | | | | | | | | | | | | | | | | | | | | | | | | | | | | | | | | | | | | | | | | | | | | | | | | | | | | | | | | | | | | | | | | | | | | | | | | | | | | | | | | | | | | | | | | | | | | | | | | | | | | | | | | | | | | | | | | | | | | | | | | | | | | | | | | | | | | | | | | | | | | | | | | | | | | | | | | | | | | | | | | | | | | | | | | | | | | | | | | | | | | | | | | | | | | | | | | | | | | | | | | | | | | | | | | | | | | | | | | | | | | | | | | | | | | | | | |  |
| **Context** | | | | | | | | | | | | | | | | | | | | | | | | | | | | | | | | | | | | | | | | | | | | | | | | | **Mechanisms** | | | | | | | | | | | | | | | | | | | | | | | | | | | | | | | | | | | | | | | | | | | | | | | | | | | | | | | | | | | | | | | | | | | | | | | | | | | | | | **Outcomes** | | | | | | | | | | | | | | | | | | | | | | | | | | | | | | | | | | | | | | | | | | | | | | | | **CMOCs** | | | | | | | | | | | | | | | | | | | | | | | | | | | | | | | | | | | | | | | | | | | | | | | | | | | | | | | | | | | | | | | | | | | | | | | | | | | | | | | | | | | | | | | | | | | | | | | | |  |
| Seven countries, differing contexts, though all supported by same INGO (AIFO) and had been running for minimum 5 years. 3 projects covered large areas and managed by government; 4 more restricted coverage and managed by NGOs.  -incentives: community recognition 22.7%; awards 9.1%; multiple 68.2%.  Experiences  - 65.3% personal decision to become CBR; 30.6% community  - 94.7% preform multiple CBR activities  - only 28.2% perform no other non-CBR related activities.  Former volunteers  -25.6% identified no time as reason for leaving work  -14.0% received permanent job  -18.6% identified multiple reasons | | | | | | | | | | | | | | | | | | | | | | | | | | | | | | | | | | | | | | | | | | | | | | | | | 1.CBR volunteers have varying demographic profiles, with the majority being involved in multiple CBR activities (multi-skilled workers)  2. Recognition of barriers CBR workers face in specific contexts (eg. Time, resource, knowledge) important to increase satisfaction and subsequently retention/motivation of health workers.  3.Personal decision/self efficacy big driver for individuals working in CBR, therefore strategies that aim to increase this are important to maintain workforce – ‘self-efficacy can be modified educationally by having credible role models, having observational and participatory learning activities, breaking down tasks into smaller steps and practicing these’.  4. Lack of time that CBR volunteers have work is factor. | | | | | | | | | | | | | | | | | | | | | | | | | | | | | | | | | | | | | | | | | | | | | | | | | | | | | | | | | | | | | | | | | | | | | | | | | | | | | | Self-efficacy or behaviour specific confidence in one’s ability to preform CBR-related tasks found to be significant with satisfaction.  Barriers had an inverse relationship with satisfaction.  Barriers included time and resources and also knowledge and skills of CBR worker.  Outcome expectations also had an inverse relationship with satisfaction.  Some form of compensation, e.g, in the form of travel and meals, was a factor in staff retention and motivation. | | | | | | | | | | | | | | | | | | | | | | | | | | | | | | | | | | | | | | | | | | | | | | | | 1.Decreasing barriers to CBR work can increase satisfaction of CBR workers  2.CBR workers who have more ambition, may become less satisfied if outcome expectations are not met.  3.Financial incentives, including regular salaries, for CBR workers may help to increase retention of workers and thus sustainability of programmes.  4.Self-efficacy may be pivotal for staff retention and performance, via other attitudinal variables like job satisfaction.  5.CBR volunteers should be both chosen by their communities and also choose to be involved in such work.  6. Incentives for volunteer work will vary between individuals and context; however, it is important to recognize the non-financial incentives, such as community recognition and certificates/awards, as extrinsic motivational factors.  7. CBR volunteers usually preform multiple CBR activities, which in each new rehabilitation context/programme should be investigated to see how such work impacts, either positively or negatively, on self-efficacy or barriers to preforming job tasks. | | | | | | | | | | | | | | | | | | | | | | | | | | | | | | | | | | | | | | | | | | | | | | | | | | | | | | | | | | | | | | | | | | | | | | | | | | | | | | | | | | | | | | | | | | | | | | | | |  |
| **Title:** Community-based rehabilitation and orthopaedic surgery for children with motor impairment in an African context | | | | | | | | | | | | | | | | | | | | | | | | | | | | | | | | | | | | | | | | | | | | | | | | | | | | | | | | | | | | | | | | | | | | | | | | | | | | | | | | | | | | | | | | | | | | | | | | | | | | | | | | | | | | | | | | | | | | | | | | | | | | | | | | | | | | | | | | | | | | | | | | | | | | | | | | | | | | | | | | | | | | | | | | | | | | | | | | | | | | | | | | | | | | | | | | | | | | | | | | | | | | | | | | | | | | | | | | | | | | | | | | | | | | | | | | | | | | | | | | | | | | | | | | | | | | | | | | | | | | | | | | | | | | | | | |  |
| **Authors:** Penny, N., Zulianello, R., Dreise, M. & Steenbeek, M. | | | | | | | | | | | | | | | | | | | | | | | | | | | | | | | | | | | | | | | | | | | | | | | | | | | | | | | | | | | | | | | | | | | | | | | | | | | | | | | | | | | | | | | | | | | | | | | | | | | | | | | | | | | | | | | | | | | | | | | | | | | | | | | | | | | | | | | | | | | | | | | | | | | | | | | | | | | | | | | | | | | | | | | | | | | | | | | | | | | | | | | | | | | | | | | | | | | | | | | | | | | | | | | | | | | | | **Year:** 2007 | | | | | | | | | | | | | | | | | | | | | | | | | | | | | | | | | | | | | | | | | | | | | | | | | | | | | | | | | | |  |
| **Summary:** Multifaceted CBR programme in Uganda work surgical care, medical rehabilitation and community rehabilitation with lay health workers. | | | | | | | | | | | | | | | | | | | | | | | | | | | | | | | | | | | | | | | | | | | | | | | | | | | | | | | | | | | | | | | | | | | | | | | | | | | | | | | | | | | | | | | | | | | | | | | | | | | | | | | | | | | | | | | | | | | | | | | | | | | | | | | | | | | | | | | | | | | | | | | | | | | | | | | | | | | | | | | | | | | | | | | | | | | | | | | | | | | | | | | | | | | | | | | | | | | | | | | | | | | | | | | | | | | | | | | | | | | | | | | | | | | | | | | | | | | | | | | | | | | | | | | | | | | | | | | | | | | | | | | | | | | | | | | |  |
| **Setting** | | **Design/**  **Method** | | | | | | | | | | | | | | | | | | | | **Population** | | | | | | | | | | | | | | | | | | | | | | | | | | | | | | | | **Intervention** | | | | | | | | | | | | | | | | | | | | | | | | | | | | | | | | | | | | | | | | | | | | | **Workforce Characteristics** | | | | | | | | | | | | | | | | | | | | | | | | | | | | | | | | | | | | | | | | | | | | | | | | | | | | | | | | | | | | | | | | | | | | | | | | | | | | | | | | | | | | | | | | | | | | | | | | | | | | | | | | | | | | | | | | | | | | | | | | | | | | | | | | | | | | | | | | | | | | | | | | | | | | | | | | | | | | | | | | | | | | | | | | | | | | |  |
|  |  |  |  |  |  |  |  |  |  |  |  |  |  |  |  |  |  |  |  |  |  |  |  |  |  |  |  |  |  |  |  |  |  |  |  |  |  |  |  |  |  |  |  |  |  |  |  |  |  |  |  |  |  |  |  |  |  |  |  |  |  |  |  |  |  |  |  |  |  |  |  |  |  |  |  |  |  |  |  |  |  |  |  |  |  |  |  |  |  |  |  |  |  |  |  |  |  |  | **Cadres** | | | | | | | | | | | | | | | | | | | | | | | | | | | | | | | | | | | | | | | | | | | | | **Description** | | | | | | | | | | | | | | | | | | | | | | | | | | | | | | | | | | | | | | | | | | | | | | | | | | | | | | | | | | | | **Training** | | | | | | | | | | | | | | | | | | | | | | | | | | | | | | | | | | | | | | | | | | | | | **Supervision** | | | | | | | | | | | | | | | | | | | | | | |  |
| Multiple districts, Uganda | | Quantitative.  Survey, records of programme  enrollment over a 6 year project. | | | | | | | | | | | | | | | | | | | | Children with treatable chronic motor impairment | | | | | | | | | | | | | | | | | | | | | | | | | | | | | | | | Uganda Children’s Orthopaedic Rehabilitation Project, established by CBM, began in 1996 after recognizing the high levels children with of physical disabilities To raise awareness in communities on the causes and potential treatments of disability. Programme offered reconstructive orthopaedic surgery and physical rehabilitation services as part of CBR project. | | | | | | | | | | | | | | | | | | | | | | | | | | | | | | | | | | | | | | | | | | | | | Community based rehabilitation (CBR) workers, physiotherapists, and orthopaedic surgeons. | | | | | | | | | | | | | | | | | | | | | | | | | | | | | | | | | | | | | | | | | | | | | CBR Workers from local community identified children with disabilities and provided home care after seen by professional.  Physiotherapists gave secondary assessment and provided advice to the CBR workers on appropriate rehabilitation in the community, as well as follow-up work in the communities.  Orthopaedic surgeons provided surgical services with more specialized (often expatriate) surgeons teaching national surgeons. | | | | | | | | | | | | | | | | | | | | | | | | | | | | | | | | | | | | | | | | | | | | | | | | | | | | | | | | | | | | Not discussed for CBR workers. Training of surgeons. Programme helped train national orthopaedic staff including surgeons. Capacity building at all levels of project, including the formation of training centres. Collaborations with Ugandan healthcare training schools. | | | | | | | | | | | | | | | | | | | | | | | | | | | | | | | | | | | | | | | | | | | | | Not highly discussed, for CBR workers physiotherapists would follow-up in villages and provide advice. They also supervised local rehabilitation workers in rehabilitation hostels.  **Quality: NA** | | | | | | | | | | | | | | | | | | | | | | |  |
| **CMOCs** | | | | | | | | | | | | | | | | | | | | | | | | | | | | | | | | | | | | | | | | | | | | | | | | | | | | | | | | | | | | | | | | | | | | | | | | | | | | | | | | | | | | | | | | | | | | | | | | | | | | | | | | | | | | | | | | | | | | | | | | | | | | | | | | | | | | | | | | | | | | | | | | | | | | | | | | | | | | | | | | | | | | | | | | | | | | | | | | | | | | | | | | | | | | | | | | | | | | | | | | | | | | | | | | | | | | | | | | | | | | | | | | | | | | | | | | | | | | | | | | | | | | | | | | | | | | | | | | | | | | | | | | | | | | | | | |  |
| **Context** | | | | | | | | | | | | | | | | | | | | | | | | | | | | | | | | | | | | | | | | | | | | | | | | | | | | | | | | | | | | | | | | | | | | | | | | | | | | | | | **Mechanisms** | | | | | | | | | | | | | | | | | | | | | | | | | | | | | | | | | | | | | | | | | | | | | | | | | | | | | | | | | | | | | | | | | | | | | | | | | | | | | | | | | **Outcomes** | | | | | | | | | | | | | | | | | | | | | | | | | | | | | | | | | | | | | | | | | | | | | | | | | | | | | | | | | | | | | | | | | | **CMOCs** | | | | | | | | | | | | | | | | | | | | | | | | | | | | | | | | | | | | | | | | | | | | | |  |
| Rehabilitation Project, established by international NGO, to treat children with disabilities, many of which were influenced by the countries political instability and resulting reducing in vaccination campaigns.  Programme was integrated and comprehensive.  Project was a network of 12 CBR projects, with several NGOS, government agencies, service providers and community groups involved. The orthopaedic surgery component acted as the linking factor between all projects, and assisted with coordination of projects.CBR workers all from local communities and speak the local language.Referral assessment of motor impairments provided by national physiotherapist, who provided a specific diagnosis and provided a treatment course (for CBR to undertake within villages) and referral. Provided caregiver education, and follow-up after children returned from rehabilitation centres.If referred by physiotherapist, children requiring surgery were transported to appropriate hospital.As soon as possible post surgery, children were transferred to rehabilitation centres which full-time professional local staff with physiotherapy supervision ran. | | | | | | | | | | | | | | | | | | | | | | | | | | | | | | | | | | | | | | | | | | | | | | | | | | | | | | | | | | | | | | | | | | | | | | | | | | | | | | | 1.Collaboration of organizations and different departments, e.g. Government, NGOs, community, teaching institutions.  2.Multi-tiered system with resources and plans at all levels. CBR workers from local community to identify and refer individuals to more specialized services while doing community awareness; Oversaw and training by consultants (expatriate).  3. Necessary systems pre-existing in place for type of work – transport, post surgical care, organisations and some health workforce capacity at more specialized level.  4. Capacity building of national staff throughout all levels of project. Orthopaedic clinical officers – a cadre of health worker in Uganda specialized in primary orthopaedic care and assigned to health centres  5. Central orthopaedic surgical unit and rehab center became the focal point around which coordination could be organized.  6. Continuum of care from village identification mobilization , treatment rehabilitation, appliance fitting, follow-up in the village. | | | | | | | | | | | | | | | | | | | | | | | | | | | | | | | | | | | | | | | | | | | | | | | | | | | | | | | | | | | | | | | | | | | | | | | | | | | | | | | | | By 2001, 5004 children were evaluated by participating CBR programme with 874 receiving surgery.  Surgical care provided as close to possible to local community, with 7 district hospitals on rotation. Throughout process however, this was consolidated to 3 sites, with one specialized surgical centre being established in the major city, linking with the University and providing training of national surgeons.Capacity building of national staff occurred throughout all levels of project. Main focus was on orthopaedic clinical officers – a cadre of health worker in Uganda specialized in primary orthopaedic care and assigned to health centres.. Central surgery unit became a training centre of national and regional orthopaedic surgeons specialized on reconstructive surgery. Programme identified ‘recipe for success’ being: CBR, physiotherapy, orthopaedic surgery, rehabilitation hostels, appliance workshops, and transportation system. | | | | | | | | | | | | | | | | | | | | | | | | | | | | | | | | | | | | | | | | | | | | | | | | | | | | | | | | | | | | | | | | | | 1.Multi-tiered system of rehabilitation, with resources, clear job descriptions and plans at all levels.  2.System of general skills in community, to more specialized skills with strong referral system and links.  3.Focal point or institution to coordinate activities  4.Continuum of care from community to specialized services, including assisting in accessing these services (transportation) and follow-up.  5.CBR workers are key in community-based rehabilitation, awareness raising and liaison in the villages at the outset to identify kids with disabilities, and assisting to access health care systems.  6.Capacity building of national programmes, institutions and human resources should be conducted at all levels of implementation.. | | | | | | | | | | | | | | | | | | | | | | | | | | | | | | | | | | | | | | | | | | | | | |  |
| **Title: Integrating community-based rehabilitation and leprosy rehabilitation services into an inclusive development approach** | | | | | | | | | | | | | | | | | | | | | | | | | | | | | | | | | | | | | | | | | | | | | | | | | | | | | | | | | | | | | | | | | | | | | | | | | | | | | | | | | | | | | | | | | | | | | | | | | | | | | | | | | | | | | | | | | | | | | | | | | | | | | | | | | | | | | | | | | | | | | | | | | | | | | | | | | | | | | | | | | | | | | | | | | | | | | | | | | | | | | | | | | | | | | | | | | | | | | | | | | | | | | | | | | | | | | | | | | | | | | | | | | | | | | | | | | | | | | | | | | | | | | | | | | | | | | | | | | | | | | | | | | | | | | | | |  |
| **Authors: Finkenflügel, H. & Rule, S.** | | | | | | | | | | | | | | | | | | | | | | | | | | | | | | | | | | | | | | | | | | | | | | | | | | | | | | | | | | | | | | | | | | | | | | | | | | | | | | | | | | | | | | | | | | | | | | | | | | | | | | | | | | | | | | | | | | | | | | | | | | | | | | | | | | | | | | | | | | | | | | | | | | | | | | | | | | | | | | | | | | | | | | | | | | | | | | | | | | | | | | | | | | | | | | | | | | | | | | | | | | | | | | | | | | | | | **Year: 2008** | | | | | | | | | | | | | | | | | | | | | | | | | | | | | | | | | | | | | | | | | | | | | | | | | | | | | | | | | | |  |
| **Summary:** Discussion on a mid-level cadre for community based rehabilitation, specifically for persons with leprosy, including description and recommendations | | | | | | | | | | | | | | | | | | | | | | | | | | | | | | | | | | | | | | | | | | | | | | | | | | | | | | | | | | | | | | | | | | | | | | | | | | | | | | | | | | | | | | | | | | | | | | | | | | | | | | | | | | | | | | | | | | | | | | | | | | | | | | | | | | | | | | | | | | | | | | | | | | | | | | | | | | | | | | | | | | | | | | | | | | | | | | | | | | | | | | | | | | | | | | | | | | | | | | | | | | | | | | | | | | | | | | | | | | | | | | | | | | | | | | | | | | | | | | | | | | | | | | | | | | | | | | | | | | | | | | | | | | | | | | | |  |
| **Setting** | | **Design/**  **Methods** | | | | | | | | | | | | | | | | | | | **Population** | | | | | | | | | | | | | | | | | | | | | | | | | | | | | | | | | | | | | | | | | | | | | | | **Intervention** | | | | | | | | | | | | | | | | | | | | | | | | | | | | | | | **Workforce Characteristics** | | | | | | | | | | | | | | | | | | | | | | | | | | | | | | | | | | | | | | | | | | | | | | | | | | | | | | | | | | | | | | | | | | | | | | | | | | | | | | | | | | | | | | | | | | | | | | | | | | | | | | | | | | | | | | | | | | | | | | | | | | | | | | | | | | | | | | | | | | | | | | | | | | | | | | | | | | | | | | | | | | | | | | | | | | | | |  |
|  |  |  | | | | | | | | | | | | | | | | | | | |  | | | | | | | | | | | | | | | | | | | | | | | | | | | | | | | | | | | | | | | | | | | | | |  | | | | | | | | | | | | | | | | | | | | | | | | | | | | | | | **Cadres** | | | | | | | | | | | | | | | | | | | | | | | | | | | | | | | | | | | | | | | | | | | | | **Description** | | | | | | | | | | | | | | | | | | | | | | | | | | | | | | | | | | | | **Training** | | | | | | | | | | | | | | | | | | | | | | | | | | | | | | | | | | | | | | | | | | | | | | | | | | **Supervision** | | | | | | | | | | | | | | | | | | | | | | | | | | | | | | | | | | | | | | | | **Misc.** | | |
| N/A | Review and analysis | | | | | | | | | | | | | | | | | | | | Persons with leprosy and disabilities resulting form leprosy | | | | | | | | | | | | | | | | | | | | | | | | | | | | | | | | | | | | | | | | | | | | | | Leprosy rehabilitation services; community based rehabilitation for persons with disabilities from leprosy | | | | | | | | | | | | | | | | | | | | | | | | | | | | | | | Mid-level cadre for Community Based Rehabilitation | | | | | | | | | | | | | | | | | | | | | | | | | | | | | | | | | | | | | | | | | | | | | Assess, instruct family members, make referrals, coordinate with other organizations and stakeholders, and organize services for persons with disabilities. “Change agents” | | | | | | | | | | | | | | | | | | | | | | | | | | | | | | | | | | | | Suggested CBR workers have been trained by rehabilitation professionals such as: occupational therapists, physiotherapists, vocational trainers etc. | | | | | | | | | | | | | | | | | | | | | | | | | | | | | | | | | | | | | | | | | | | | | | | | | | Suggested CBR workers have been supervised by rehabilitation professionals such as: occupational therapists, physiotherapists, vocational trainers etc. | | | | | | | | | | | | | | | | | | | | | | | | | | | | | | | | | | | | | | | | **Quality:NA** | | |  |
| **CMOCs** | | | | | | | | | | | | | | | | | | | | | | | | | | | | | | | | | | | | | | | | | | | | | | | | | | | | | | | | | | | | | | | | | | | | | | | | | | | | | | | | | | | | | | | | | | | | | | | | | | | | | | | | | | | | | | | | | | | | | | | | | | | | | | | | | | | | | | | | | | | | | | | | | | | | | | | | | | | | | | | | | | | | | | | | | | | | | | | | | | | | | | | | | | | | | | | | | | | | | | | | | | | | | | | | | | | | | | | | | | | | | | | | | | | | | | | | | | | | | | | | | | | | | | | | | | | | | | | | | | | | | | | | | | | | | | | |  |
| **Context** | | | | | | | | | | | | | | | | | | | | | | | | | | | | | | | | | | | | | | **Mechanisms** | | | | | | | | | | | | | | | | | | | | | | | | | | | | | | | | | | | | | | | | | | | | | | | | | | | | | | | | | **Outcomes** | | | | | | | | | | | | | | | | | | | | | | | | | | | | | | | | | | | | | | | | | | | | | | | | | | | | | | | | | | | | | | | | | | | | | | | | **CMOCs** | | | | | | | | | | | | | | | | | | | | | | | | | | | | | | | | | | | | | | | | | | | | | | | | | | | | | | | | | | | | | | | | | | | | | | | | | | | | | | | | | | | | | | | | | | | | | | | | | | | | | | | | |  |
| Community based rehabilitation by mid-level cadres.  CBR and livelihoods programmes embedded in persons with disabilities own communities.  Current community based rehabilitation programmes part of community development and not vertical programmes.  With development of mid-level cadres, professionals who were previously trainers have taken more managerial role in programmes.  Support structures for community based rehabilitation workers crucial for programme effectiveness. | | | | | | | | | | | | | | | | | | | | | | | | | | | | | | | | | | | | | | 1.CBR workers competencies will differ depending on programme type (vertical or horizontal) – should be encouraging horizontal programming approaches  2.Integration of services  3. Lack of evidence on effectiveness of CBR programmes can influence support and prioritization of such services  4. Rehabilitation professionals (physiotherapists etc.) acting as supervisors, trainers and programme managers can lead mid-level cadres to promote rehabilitation in the community activities. | | | | | | | | | | | | | | | | | | | | | | | | | | | | | | | | | | | | | | | | | | | | | | | | | | | | | | | | | Community based rehabilitation workers involved in activates in their communities such as advocacy and lobbying  CBR workers act as ‘change agents’  Mid-level cadres with additional referral skills positioned within human rights based approach.  CBR workers in their new “role” working in inclusive development might require different skills from their trainers and supervisors – for instance consider including social workers and others knowledgeable on social development.  Professionals’ role might move to more referral support and programme management.  Professionals have less direct contact with people from the community in an inclusive development approach.  CBR programmes face difficulties proving their worth due to lack of evidence.  CBR workers working with leprosy should have leprosy specific expertise | | | | | | | | | | | | | | | | | | | | | | | | | | | | | | | | | | | | | | | | | | | | | | | | | | | | | | | | | | | | | | | | | | | | | | | | 1.Persons with disabilities should be involved in the development of CBR programmes  2.CBR Matrix activities – integrated and inclusive development programmes  3.Social approach including raising awareness, advocacy programmes and empowering PWD and their families  4.Increase evidence base for community based rehabilitation programmes  5.Evidenced-based practice for community based rehabilitation workers.  6.CBR workers should have specific skills and knowledge on a disease of type of disability, while being knowledgeable on CBR as a whole and social development approach to rehabilitation  7.CBR workers should be the link between communities and more specialized services, and be accountable to these communities.  8.Trainers and therapists should be trained as managers of mid-level rehabilitation cadres  9.“Effective care-chains” should be created for rehabilitation services with personnel having a set of competencies driven by client’s needs, which interlink with other workers to provide comprehensive services.  10.More specialized rehabilitation cadres can act as trainers and/or supervisors  11.Supervisors/trainers should be competent in both clinical skills and supervision/training skills  12. Team work, with rehabilitation cadres operating together, is essential for effectiveness of community based rehabilitation programmes.  12. Changing job descriptions of community based rehabilitation workers with the development of CBR activities | | | | | | | | | | | | | | | | | | | | | | | | | | | | | | | | | | | | | | | | | | | | | | | | | | | | | | | | | | | | | | | | | | | | | | | | | | | | | | | | | | | | | | | | | | | | | | | | | | | | | | | | |  |
| **Title:** Impact of Community-Based Rehabilitation Programmes: The Case of Palestine | | | | | | | | | | | | | | | | | | | | | | | | | | | | | | | | | | | | | | | | | | | | | | | | | | | | | | | | | | | | | | | | | | | | | | | | | | | | | | | | | | | | | | | | | | | | | | | | | | | | | | | | | | | | | | | | | | | | | | | | | | | | | | | | | | | | | | | | | | | | | | | | | | | | | | | | | | | | | | | | | | | | | | | | | | | | | | | | | | | | | | | | | | | | | | | | | | | | | | | | | | | | | | | | | | | | | | | | | | | | | | | | | | | | | | | | | | | | | | | | | | | | | | | | | | | | | | | | | | | | | | | | | | | | | | | |  |
| **Authors:** Eide, AH. | | | | | | | | | | | | | | | | | | | | | | | | | | | | | | | | | | | | | | | | | | | | | | | | | | | | | | | | | | | | | | | | | | | | | | | | | | | | | | | | | | | | | | | | | | | | | | | | | | | | | | | | | | | | | | | | | | | | | | | | | | | | | | | | | | | | | | | | | | | | | | | | | | | | | | | | | | | | | | | | | | | | | | | | | | | | | | | | | | | | | | | | | | | | | | | | | | | | | | | | | | | | | | | | | | | **Year:** 2006 | | | | | | | | | | | | | | | | | | | | | | | | | | | | | | | | | | | | | | | | | | | | | | | | | | | | | | | | | | | | |  |
| **Summary:** Description and 10 year impact assessment of CBR programme using village-based community rehabilitation workers | | | | | | | | | | | | | | | | | | | | | | | | | | | | | | | | | | | | | | | | | | | | | | | | | | | | | | | | | | | | | | | | | | | | | | | | | | | | | | | | | | | | | | | | | | | | | | | | | | | | | | | | | | | | | | | | | | | | | | | | | | | | | | | | | | | | | | | | | | | | | | | | | | | | | | | | | | | | | | | | | | | | | | | | | | | | | | | | | | | | | | | | | | | | | | | | | | | | | | | | | | | | | | | | | | | | | | | | | | | | | | | | | | | | | | | | | | | | | | | | | | | | | | | | | | | | | | | | | | | | | | | | | | | | | | | |  |
| **Setting** | | | | | | | | | | | | | **Design/**  **Methods** | | | | | | | | | | | | | | | | | | | | | | | | | **Population** | | | | | | | | | | | | | | | | | | | | | | | | | | | | | | | | | | | | **Intervention** | | | | | | | | | | | | | | | | | | | | | | | | | | | | | | | | | | | | | | **Workforce Characteristics** | | | | | | | | | | | | | | | | | | | | | | | | | | | | | | | | | | | | | | | | | | | | | | | | | | | | | | | | | | | | | | | | | | | | | | | | | | | | | | | | | | | | | | | | | | | | | | | | | | | | | | | | | | | | | | | | | | | | | | | | | | | | | | | | | | | | | | | | | | | | | | | | | | | | | | | | | | | | | | | |  |
|  |  |  |  |  |  |  |  |  |  |  |  |  |  |  |  |  |  |  |  |  |  |  |  |  |  |  |  |  |  |  |  |  |  |  |  |  |  |  |  |  |  |  |  |  |  |  |  |  |  |  |  |  |  |  |  |  |  |  |  |  |  |  |  |  |  |  |  |  |  |  |  |  |  |  |  |  |  |  |  |  |  |  |  |  |  |  |  |  |  |  |  |  |  |  |  |  |  |  |  |  |  |  |  |  |  |  |  |  |  |  |  | **Cadres** | | | | | | | | | | | | | | | | | | | | | | | | | | | | | | | | | | | | **Description** | | | | | | | | | | | | | | | | | | | | | | | | | | | | | **Training** | | | | | | | | | | | | | | | | | | | | | | | | | | | | | | | | | | | | | | | | | | | | | | | | | | | | | | **Supervision** | | | | | | | | | | | | | | | | | | | | | | | **Misc.** | | | | | | | | | | | | | | | | | |  |
| Palestine.  Regional Committees in Nablus, Jenin, South Region, Central Region and Gaza. | | | | | | | | | | | | | Case study, using baseline study follow-up (questionnaire) and a record audit (structured interviews). | | | | | | | | | | | | | | | | | | | | | | | | | 1075 individuals having received CBR services (questionnaire) and 57 service users for structured interview. | | | | | | | | | | | | | | | | | | | | | | | | | | | | | | | | | | | | Multi-level (nationally, regionally and community) multi-faceted CBR programme, coordinated by National Committee for Rehabilitation but implemented by NGOs across Palestine. | | | | | | | | | | | | | | | | | | | | | | | | | | | | | | | | | | | | | | Community rehabilitation workers (CRWs) | | | | | | | | | | | | | | | | | | | | | | | | | | | | | | | | | | | | Work independently and usually in villages in while they live. Refer individuals to known, appropriate, existing structures of support. | | | | | | | | | | | | | | | | | | | | | | | | | | | | | Not discussed, however work with NGOs in district and in regional committees likely having varying training across sties. “Extensive initial training, followed by regular training periods”. Practical trainings focused on the principles of CBR. | | | | | | | | | | | | | | | | | | | | | | | | | | | | | | | | | | | | | | | | | | | | | | | | | | | | | | CRWs supervised at village level. | | | | | | | | | | | | | | | | | | | | | | | Varying levels of support expected depending on area, including access to more specialized services.  **Quality – 2**  MMAT - MM | | | | | | | | | | | | | | | | | |  |
| **CMOCs** | | | | | | | | | | | | | | | | | | | | | | | | | | | | | | | | | | | | | | | | | | | | | | | | | | | | | | | | | | | | | | | | | | | | | | | | | | | | | | | | | | | | | | | | | | | | | | | | | | | | | | | | | | | | | | | | | | | | | | | | | | | | | | | | | | | | | | | | | | | | | | | | | | | | | | | | | | | | | | | | | | | | | | | | | | | | | | | | | | | | | | | | | | | | | | | | | | | | | | | | | | | | | | | | | | | | | | | | | | | | | | | | | | | | | | | | | | | | | | | | | | | | | | | | | | | | | | | | | | | | | | | | | | | | | | | |  |
| **Context** | | | | | | | | | | | | | | | | | | | | | | | | | | | | | | | | | | | | | | | | | | | | | | | | | | | | | | | | | | | **Mechanisms** | | | | | | | | | | | | | | | | | | | | | | | | | | | | | | | | | | | | | | | | | | | | | | | | | | | | | | | | | **Outcomes** | | | | | | | | | | | | | | | | | | | | | | | | | | | | | | | | | | | | | | | | | | | | | | | | | | | | | | | | | | | | | | | | | | **CMOCs** | | | | | | | | | | | | | | | | | | | | | | | | | | | | | | | | | | | | | | | | | | | | | | | | | | | | | | | | | | | | | | | | | | | | | | | | | | | | | | | | | | | | | | | | | |  |
| CBR Programme established in 1989, coordinated by Central National Committee for Rehabilitation and 23 implementing NGO partners group in five Regional Committees.  Decentralized with autonomous programmes at regional levels.  Practical component designed around notions of CBR in the 1990s.  Community surveys at introduction of programme, with CRWs then using two-pronged approach in the communities, one at community level for change promotion and mobilization and one at individual level to bring about positive situational changes.  Programme covers approximately 50% of population in West Bank and 75% in Gaza.  Political instability, socio-cultural context and also great changes in context over the 10 years since beginning of programme. | | | | | | | | | | | | | | | | | | | | | | | | | | | | | | | | | | | | | | | | | | | | | | | | | | | | | | | | | | | 1.Good filing system for record keeping; however in this study may have faced issues when their recommended documenting system (by WHO) was modified after project already started.  2.CBR workers are able to work independently in their communities with some supervision, at the local level  3.Service users of CBR identify CBR workers as positively contributing to improvements in their daily lives.  4. CBR workers can work at both community and individual level to promote positive change for PWD | | | | | | | | | | | | | | | | | | | | | | | | | | | | | | | | | | | | | | | | | | | | | | | | | | | | | | | | | Increase awareness on disability issues for communities.  Positive attitude change towards PWD in communities.  Positive view of impact of CBR by service users.  Activity of daily living (ADL) assessment showed considerable progress, as rated by service users.  Conclusion that many service users were given appropriate assessment, treatment, training, guidance and devices that positively contributed to their daily living due to the CBR programme and CRWs acting in the community. | | | | | | | | | | | | | | | | | | | | | | | | | | | | | | | | | | | | | | | | | | | | | | | | | | | | | | | | | | | | | | | | | | 1.Training should focus on practical components of implementing CBR in communities, after an extensive survey of the context and the disability situation, rehabilitation needs.  2.Monitoring of initial context survey periodically  3.CBR workers should have strong referral skills, with knowledge on resources available for PWD  4.CBR workers should be from the same villages in which they work in order to have increased acceptability by communities and also understanding of barriers faced by PWD.  5.Independence of CBR workers may be due to being from the villages in which they live.  6.Ability to work independently may be especially important in areas with great contextual difference and support across regions/districts and political instability.  7.CBR workers should be trained to work at both the individual level and community level, with differing skills depending on the needs in that context. | | | | | | | | | | | | | | | | | | | | | | | | | | | | | | | | | | | | | | | | | | | | | | | | | | | | | | | | | | | | | | | | | | | | | | | | | | | | | | | | | | | | | | | | | |  |
| **Title:** China-Australia-Hong Kong tripartite community mental health training program | | | | | | | | | | | | | | | | | | | | | | | | | | | | | | | | | | | | | | | | | | | | | | | | | | | | | | | | | | | | | | | | | | | | | | | | | | | | | | | | | | | | | | | | | | | | | | | | | | | | | | | | | | | | | | | | | | | | | | | | | | | | | | | | | | | | | | | | | | | | | | | | | | | | | | | | | | | | | | | | | | | | | | | | | | | | | | | | | | | | | | | | | | | | | | | | | | | | | | | | | | | | | | | | | | | | | | | | | | | | | | | | | | | | | | | | | | | | | | | | | | | | | | | | | | | | | | | | | | | | | | | | | | | | | | | |  |
| **Authors:** Ng, CH., Ma, H., Yu, X., Chui, H., Fraser, J., Chan, S., Chui, E. & Jia, FJ. | | | | | | | | | | | | | | | | | | | | | | | | | | | | | | | | | | | | | | | | | | | | | | | | | | | | | | | | | | | | | | | | | | | | | | | | | | | | | | | | | | | | | | | | | | | | | | | | | | | | | | | | | | | | | | | | | | | | | | | | | | | | | | | | | | | | | | | | | | | | | | | | | | | | | | | | | | | | | | | | | | | | | | | | | | | | | | | | | | | | | | | | | | | | | | | | | | | | | | | | | | | | | | | | | | | Year: 2009 | | | | | | | | | | | | | | | | | | | | | | | | | | | | | | | | | | | | | | | | | | | | | | | | | | | | | | | | | | | | |  |
| Summary: Description of a collaborative training programme for community rehabilitation professionals across three settings | | | | | | | | | | | | | | | | | | | | | | | | | | | | | | | | | | | | | | | | | | | | | | | | | | | | | | | | | | | | | | | | | | | | | | | | | | | | | | | | | | | | | | | | | | | | | | | | | | | | | | | | | | | | | | | | | | | | | | | | | | | | | | | | | | | | | | | | | | | | | | | | | | | | | | | | | | | | | | | | | | | | | | | | | | | | | | | | | | | | | | | | | | | | | | | | | | | | | | | | | | | | | | | | | | | | | | | | | | | | | | | | | | | | | | | | | | | | | | | | | | | | | | | | | | | | | | | | | | | | | | | | | | | | | | | |  |
| **Setting** | | | | | | **Design/**  **Method** | | | | | | | | | | | | | | | | | | | **Population** | | | | | | | | | | | | | | | | | | | | | | | | | | | | | | | **Intervention** | | | | | | | | | | | | | | | | | | | | | | | | | | | | | | | | | | | | | | | **Workforce Characteristics** | | | | | | | | | | | | | | | | | | | | | | | | | | | | | | | | | | | | | | | | | | | | | | | | | | | | | | | | | | | | | | | | | | | | | | | | | | | | | | | | | | | | | | | | | | | | | | | | | | | | | | | | | | | | | | | | | | | | | | | | | | | | | | | | | | | | | | | | | | | | | | | | | | | | | | | | | | | | | | | | | | | | | | | | | | | | | | | | |  |
|  |  |  |  |  |  |  |  |  |  |  |  |  |  |  |  |  |  |  |  |  |  |  |  |  |  |  |  |  |  |  |  |  |  |  |  |  |  |  |  |  |  |  |  |  |  |  |  |  |  |  |  |  |  |  |  |  |  |  |  |  |  |  |  |  |  |  |  |  |  |  |  |  |  |  |  |  |  |  |  |  |  |  |  |  |  |  |  |  |  |  |  |  |  |  | **Cadres** | | | | | | | | | | | | | | | | | | | | | | | | | | | | | | | | | | | | | | | | | | | | | | | | | | | | | | | | | | **Description** | | | | | | | | | | | | | | | | | | | **Training** | | | | | | | | | | | | | | | | | | | | | | | | | | | | | | | | | | | | | | | | | | | | | | | | | | | | | | | | | **Supervision** | | | | | | | | | | | | | | | | | | | | | **Misc.** | | | | | | | | | | | | | | | | | | | | | |  |
| China, Hong Kong and Australia | | | | | | Programme report | | | | | | | | | | | | | | | | | | | Mental health staff, mainly in China | | | | | | | | | | | | | | | | | | | | | | | | | | | | | | | Cross-cultural and country collaboration to build culturally competency health service systems and delivery, across 2 countries in 3 training sites. Largely University and professional body-driven, with community based care being clinic centered. | | | | | | | | | | | | | | | | | | | | | | | | | | | | | | | | | | | | | | | Mental health professionals including: community psychiatrists, social workers, occupational therapists, clinical psychologists, nurses. | | | | | | | | | | | | | | | | | | | | | | | | | | | | | | | | | | | | | | | | | | | | | | | | | | | | | | | | | | Work in Community Mental Health Teams, multidisciplinary, with case managers. | | | | | | | | | | | | | | | | | | | Varied between location. Focus on Training of Trainer (3days). Workshops, practical (on site) training, lecturing, management training for senior staff (1 week). Tripartite programme involved three modules (introduction, practical training, and management training). | | | | | | | | | | | | | | | | | | | | | | | | | | | | | | | | | | | | | | | | | | | | | | | | | | | | | | | | | Integrated throughout programmes, with Training of Trainer meant to ensure clinical supervision. | | | | | | | | | | | | | | | | | | | | | Teams multidisciplinary, work out of major psychiatric hospital and have catchment population of approximately 3 million.  **Quality- NA** Descriptive report | | | | | | | | | | | | | | | | | | | | | |  |
| **CMOCs** | | | | | | | | | | | | | | | | | | | | | | | | | | | | | | | | | | | | | | | | | | | | | | | | | | | | | | | | | | | | | | | | | | | | | | | | | | | | | | | | | | | | | | | | | | | | | | | | | | | | | | | | | | | | | | | | | | | | | | | | | | | | | | | | | | | | | | | | | | | | | | | | | | | | | | | | | | | | | | | | | | | | | | | | | | | | | | | | | | | | | | | | | | | | | | | | | | | | | | | | | | | | | | | | | | | | | | | | | | | | | | | | | | | | | | | | | | | | | | | | | | | | | | | | | | | | | | | | | | | | | | | | | | | | | | | |  |
| **Context** | | | | | | | | | | | | | | | | | | | | | | | | | | | | | | | | | | | | | | | | | | | | | | | | | | | | | | | | **Mechanisms** | | | | | | | | | | | | | | | | | | | | | | | | | | | | | | | | | | | | | | | | | | | | | | | | | | | | | | | | | | | | **Outcomes** | | | | | | | | | | | | | | | | | | | | | | | | | | | | | | | | | | | | | | | | | | | | | | | | | | | | | | | | | | | | | | | | | | | | | | | **CMOCs** | | | | | | | | | | | | | | | | | | | | | | | | | | | | | | | | | | | | | | | | | | | | | | | | | | | | | | | | | | | | | | | | | | | | | | | | | | | | | | | | | | | | |  |
| Asia-Australia Mental Health (AAMH) consortium established in 2003 between medical and academic institutions in Australia and Asialink.  686 Programme supported by AAMH, Ministry of Health China, and PKUIMH for mental health reform. Programme to build workforce capacity for community mental health.  Training programme collaboratively planned with AAMH, PKUIMH, Chinese University of Hong Kong for multi-skilled case workers for mainland China. Idea to train case managers to deliver further training to peers on the mainland, under a Train the Trainer model.  3 sites had different training objectives and courses. Mainland China – Module One, including introductory 1-week training course.  Hong Kong – TOT 3 day course; Module 2 for mental health clinicians with 3 days theory and 7 days practical.  Melbourne: Management training for senior mental health staff for 1 week. | | | | | | | | | | | | | | | | | | | | | | | | | | | | | | | | | | | | | | | | | | | | | | | | | | | | | | | | 1.Mental health professionals including psychiatrists, nurses and administrators participated in training of trainer course for community mental health, who then go and train other mental health professionals.  2.External capacity for initial training in setting where internal capacity is limited, especially in diploma or university programmes.  3.Significant is the adoption of training approaches that build in flexibility for different levels of expertise and skills of the mental health trainees  4.Training programmes should emphasis partnership across sectors within government and NGO. | | | | | | | | | | | | | | | | | | | | | | | | | | | | | | | | | | | | | | | | | | | | | | | | | | | | | | | | | | | | China: 500 mental health professionals from 80 districts in mainland China participated in the TOT workshops. Professionals included: psychiatrists, nurses and administrators selected by CUHK). 400 training sessions for health professionals and other stakeholders, totaling over 50,000 people.  Hong Kong: 6 groups of 10 (n=60) mainland Chinese mental health clinicians underwent a clinical training placement with case management approach tailored to Chinese context. Management approach to facilitate the community rehabilitation workforce and stakeholders for collaborative work. Module had onsite participatory observation, fieldwork, seminars and practical workshops.  Melbourne – POST (Postgraduate Overseas Specialists Training) facilitates individualized clinical training placements for mental health clinicians from Asia-Pacific for 2 weeks up to 1 year. 45 participants from Hong Kong and 6 from mainland China. | | | | | | | | | | | | | | | | | | | | | | | | | | | | | | | | | | | | | | | | | | | | | | | | | | | | | | | | | | | | | | | | | | | | | | | 1.Programmes and training must be relevant to the context in which they will work in, so designed with training experts from that region in consultation with international experts to insure that cultural needs as they are central for the success of the adaptation of any community health care models.  2.Information/training, which has a strong practical component and works to exchange knowledge effective in training those who are inexperienced or in cross-setting exchanges. Management approach incorporated into training to facilitate collaborative work for human resources.  3. Multi-skilled case workers should have:  An understanding of community based mental health principles, e.g., case management; able to develop individual service plans for clients by exploring culturally appropriate ways to build partnerships with patients families and communities; skilled at working in multidisciplinary teams; have plans for implementation.  4.Training of Trainer important to ensure consistency and standardization, as well as increase capacity.  5. Training collaborations using academic partnerships and focusing on the needs of the staff depending on context of work to share knowledge across disciplines and resources. | | | | | | | | | | | | | | | | | | | | | | | | | | | | | | | | | | | | | | | | | | | | | | | | | | | | | | | | | | | | | | | | | | | | | | | | | | | | | | | | | | | | |  |
| **Title:** Evaluation of a community-based rehabilitation model for chronic schizophrenia in rural India | | | | | | | | | | | | | | | | | | | | | | | | | | | | | | | | | | | | | | | | | | | | | | | | | | | | | | | | | | | | | | | | | | | | | | | | | | | | | | | | | | | | | | | | | | | | | | | | | | | | | | | | | | | | | | | | | | | | | | | | | | | | | | | | | | | | | | | | | | | | | | | | | | | | | | | | | | | | | | | | | | | | | | | | | | | | | | | | | | | | | | | | | | | | | | | | | | | | | | | | | | | | | | | | | | | | | | | | | | | | | | | | | | | | | | | | | | | | | | | | | | | | | | | | | | | | | | | | | | | | | | | | | | | | | | | |  |
| **Authors:** Chattergee, S., Patel, V., Chatterjee A. & Weiss, HA. | | | | | | | | | | | | | | | | | | | | | | | | | | | | | | | | | | | | | | | | | | | | | | | | | | | | | | | | | | | | | | | | | | | | | | | | | | | | | | | | | | | | | | | | | | | | | | | | | | | | | | | | | | | | | | | | | | | | | | | | | | | | | | | | | | | | | | | | | | | | | | | | | | | | | | | | | | | | | | | | | | | | | | | | | | | | | | | | | | | | | | | | | | | | | | | | | | | | | | | | | | | | | | | | | | | **Year:** 2003 | | | | | | | | | | | | | | | | | | | | | | | | | | | | | | | | | | | | | | | | | | | | | | | | | | | | | | | | | | | | |  |
| **Summary:** Tier system using lay mental health workers to support individuals and communities in addition to clinical treatment. | | | | | | | | | | | | | | | | | | | | | | | | | | | | | | | | | | | | | | | | | | | | | | | | | | | | | | | | | | | | | | | | | | | | | | | | | | | | | | | | | | | | | | | | | | | | | | | | | | | | | | | | | | | | | | | | | | | | | | | | | | | | | | | | | | | | | | | | | | | | | | | | | | | | | | | | | | | | | | | | | | | | | | | | | | | | | | | | | | | | | | | | | | | | | | | | | | | | | | | | | | | | | | | | | | | | | | | | | | | | | | | | | | | | | | | | | | | | | | | | | | | | | | | | | | | | | | | | | | | | | | | | | | | | | | | |  |
| **Setting** | | | | | | **Design/**  **Methods** | | | | | | | | | | | | | | | | | | | **Population** | | | | | | | | | | | | | | | | | | | | | | | | | | | | | | | | | | | | | | | | **Intervention** | | | | | | | | | | | | | | | | | | | | | | | | | | | | | | | | | | | | | | | | | | | | | | | | | | | | | | | | | | | | | | | | | | | | | | | | | | | | **Workforce Characteristics** | | | | | | | | | | | | | | | | | | | | | | | | | | | | | | | | | | | | | | | | | | | | | | | | | | | | | | | | | | | | | | | | | | | | | | | | | | | | | | | | | | | | | | | | | | | | | | | | | | | | | | | | | | | | | | | | | | | | | | | | | | | | | | | | | | |  |
|  |  |  |  |  |  |  |  |  |  |  |  |  |  |  |  |  |  |  |  |  |  |  |  |  |  |  |  |  |  |  |  |  |  |  |  |  |  |  |  |  |  |  |  |  |  |  |  |  |  |  |  |  |  |  |  |  |  |  |  |  |  |  |  |  |  |  |  |  |  |  |  |  |  |  |  |  |  |  |  |  |  |  |  |  |  |  |  |  |  |  |  |  |  |  |  |  |  |  |  |  |  |  |  |  |  |  |  |  |  |  |  |  |  |  |  |  |  |  |  |  |  |  |  |  |  |  |  |  |  |  |  |  |  |  |  |  |  |  |  |  | **Cadres** | | | | | | | | | | | | | | | | | | | | | | | | | | | | **Description** | | | | | | | | | | | | | | | | | | | | | | | | | | | | | | | | | | **Training** | | | | | | | | | | | | | | | | | | | | | | | | | | | | **Supervision** | | | | | | | | | | | | | | | | | | | | | | | | | | | | | **Misc.** | | | | | | | | | | | |  |
| District of Barwani, in the state of Madhya Pradesh, India. | | | | | | Prospective, longitudinal study | | | | | | | | | | | | | | | | | | | 207 individuals with chronic schizophrenia. 127 in CBR group and 80 in out-patient care (OPC) group | | | | | | | | | | | | | | | | | | | | | | | | | | | | | | | | | | | | | | | | Three tier model of care: outpatient care including drug treatment; mental health workers in community; third tier consist of family members or community who form village health groups acting as a forum for members and to raise awareness, plan for rehabilitation and reduce social exclusion.  Common to both models are drug treatment, psycho-education, and family counseling | | | | | | | | | | | | | | | | | | | | | | | | | | | | | | | | | | | | | | | | | | | | | | | | | | | | | | | | | | | | | | | | | | | | | | | | | | | | Lay mental health workers, psychiatrist, psychologist, family groups, village groups. | | | | | | | | | | | | | | | | | | | | | | | | | | | | Mental health workers from the community and family members and community members to form local village health groups. | | | | | | | | | | | | | | | | | | | | | | | | | | | | | | | | | | Mental health workers trained for 60 days. | | | | | | | | | | | | | | | | | | | | | | | | | | | | Not discussed, however tier system with specialized services could provide supervisory aspect and mechanisms for feedback. | | | | | | | | | | | | | | | | | | | | | | | | | | | | | Responsible for 25-30 patients  **Quality – 2.5**  MMAT – Quant4, only hospital recruitment | | | | | | | | | | | |  |
| **CMOCs** | | | | | | | | | | | | | | | | | | | | | | | | | | | | | | | | | | | | | | | | | | | | | | | | | | | | | | | | | | | | | | | | | | | | | | | | | | | | | | | | | | | | | | | | | | | | | | | | | | | | | | | | | | | | | | | | | | | | | | | | | | | | | | | | | | | | | | | | | | | | | | | | | | | | | | | | | | | | | | | | | | | | | | | | | | | | | | | | | | | | | | | | | | | | | | | | | | | | | | | | | | | | | | | | | | | | | | | | | | | | | | | | | | | | | | | | | | | | | | | | | | | | | | | | | | | | | | | | | | | | | | | | | | | | | | | |  |
| **Context** | | | | | | | | | | | | | | | | | | | | | | | | | | | | | | | | | | | | | | | | | | | | | | | | | | | | | | | | | | | | | | | | | | | | | | | | | | | | | | | | **Mechanisms** | | | | | | | | | | | | | | | | | | | | | | | | | | | | | | | | | | | | | | | | | | | | | | | | | | | | | | | | | | | | | | | | | | | | | | | | | **Outcomes** | | | | | | | | | | | | | | | | | | | | | | | | | | | | | | | | | | | | | | | | | **CMOCs** | | | | | | | | | | | | | | | | | | | | | | | | | | | | | | | | | | | | | | | | | | | | | | | | | | | | | | | | | | | | | | | | | | | | | | | | | | | | | |  |
| Majority of the population are indigenous tribes people. Barwani is one of the poorest districts in India, with the indigenous population being the most sociologically disadvantaged. No medical mental health centre existed in district or neighbouring districts during time of study.  Participants firstly diagnosed by Psychiatrist. Enrolled in CBR group if living in district, and assigned to OPC group if outside the study area.  OPC consisted of services provided exclusively at a clinic, usually with one visit per month lasting 20-30 min. Seen by psychiatrist and/or psychologist, with ongoing drug treatments and family education on compliance and the illness, including rehabilitation strategies and psych education.  207 participants initially enrolled with 127 in CBR arm and 80 in OPC.  CBR arm were significantly more disadvantaged in terms of literacy, caste system and poverty compared to OPC group, and had longer duration of illness and DAS behavioural scores.  Content of CBR intervention was shaped (adapted) by consultation with patients and families and other key persons in the community.  CBR also encouraged people to maintain links with traditional healers and general practitioners. | | | | | | | | | | | | | | | | | | | | | | | | | | | | | | | | | | | | | | | | | | | | | | | | | | | | | | | | | | | | | | | | | | | | | | | | | | | | | | | | 1.Initial diagnoses and treatment plans should be designed by specialist often referred to by CHW.  2.Tier system allowing for treatment in the community with appropriate referral mechanisms to more specialized services may impact on patient’s compliance with programmes.  3.CBR workers that compliment other traditional healers can help increase effectiveness/acceptance of programmes.  4.CBR workers need strong referral mechanisms and services in place  5. CBR workers can target hard to reach populations and assist in continuation of care and compliance  6. Clinical treatment with community treatment combined is more effective than clinical treatment alone in some outcome aspects | | | | | | | | | | | | | | | | | | | | | | | | | | | | | | | | | | | | | | | | | | | | | | | | | | | | | | | | | | | | | | | | | | | | | | | | | Compliance significantly higher in CBR group with 63% fully compliant compared to 46% in CBR vs. OPC, respectively. CBR more efficient in retaining patients and families compared to OPC.  Intention-to-treat significantly higher in CBR group when using less conservative data analysis methods of LOCF.  Disability outcomes for CBR group higher than OPC.  Difference in disability outcomes for CBR group was greater for males than females | | | | | | | | | | | | | | | | | | | | | | | | | | | | | | | | | | | | | | | | | 1.CBR workers should be linked with the treating specialists so that they are aware of treatment plans and can emphasize the compliance with medicine/rehabilitation.  2.Community based services are more efficient and better at overcoming common barriers to health care such as – economic, cultural and geographical.  3.Mental health workers evidently become involved in support for families so should be taught coping strategies.  4.Programmes that engage and empower the families of PWD and their communities, and having them be partners in the design, implementation and monitoring of services are more culturally feasible and sustainable.  5.Three-tier system supports the outpatient counseling of patients, better empowering them and their families while also addressing issues cultural and society.  6. CBR workers should work within culturally appropriate health systems, for example with traditional healers.  7. For vulnerable populations (location, high stigma, poverty), clinical treatment should be combined with community outreach and have community based workers compliment clinical work. | | | | | | | | | | | | | | | | | | | | | | | | | | | | | | | | | | | | | | | | | | | | | | | | | | | | | | | | | | | | | | | | | | | | | | | | | | | | | |  |
| **Title:** Feeding difficulties in children with cerebral palsy: low-cost caregiver training in Dhaka, Bangladesh | | | | | | | | | | | | | | | | | | | | | | | | | | | | | | | | | | | | | | | | | | | | | | | | | | | | | | | | | | | | | | | | | | | | | | | | | | | | | | | | | | | | | | | | | | | | | | | | | | | | | | | | | | | | | | | | | | | | | | | | | | | | | | | | | | | | | | | | | | | | | | | | | | | | | | | | | | | | | | | | | | | | | | | | | | | | | | | | | | | | | | | | | | | | | | | | | | | | | | | | | | | | | | | | | | | | | | | | | | | | | | | | | | | | | | | | | | | | | | | | | | | | | | | | | | | | | | | | | | | | | | | | | | | | | | | |  |
| **Authors:** Adams, MS., Khan, NZ., Begum, SA., Wirz, SL., Hesketh, T. & Pring, TR. | | | | | | | | | | | | | | | | | | | | | | | | | | | | | | | | | | | | | | | | | | | | | | | | | | | | | | | | | | | | | | | | | | | | | | | | | | | | | | | | | | | | | | | | | | | | | | | | | | | | | | | | | | | | | | | | | | | | | | | | | | | | | | | | | | | | | | | | | | | | | | | | | | | | | | | | | | | | | | | | | | | | | | | | | | | | | | | | | | | | | | | | | | | | | | | | | | | | | | | | | | | | | | | | | | | **Year:** 2011 | | | | | | | | | | | | | | | | | | | | | | | | | | | | | | | | | | | | | | | | | | | | | | | | | | | | | | | | | | | | |  |
| **Summary:** Caregivers of children with cerebral palsy trained by generic therapists on appropriate feeding techniques | | | | | | | | | | | | | | | | | | | | | | | | | | | | | | | | | | | | | | | | | | | | | | | | | | | | | | | | | | | | | | | | | | | | | | | | | | | | | | | | | | | | | | | | | | | | | | | | | | | | | | | | | | | | | | | | | | | | | | | | | | | | | | | | | | | | | | | | | | | | | | | | | | | | | | | | | | | | | | | | | | | | | | | | | | | | | | | | | | | | | | | | | | | | | | | | | | | | | | | | | | | | | | | | | | | | | | | | | | | | | | | | | | | | | | | | | | | | | | | | | | | | | | | | | | | | | | | | | | | | | | | | | | | | | | | |  |
| **Setting** | | | | | | **Design/Methods** | | | | | | | | | | | | | | | | | | | | | | | | | | | | | | | | **Population** | | | | | | | | | | | | | | | | | | | | | | | | | | | | | | | | | | | | **Intervention** | | | | | | | | | | | | | | | | | | | | | | | **Workforce Characteristics** | | | | | | | | | | | | | | | | | | | | | | | | | | | | | | | | | | | | | | | | | | | | | | | | | | | | | | | | | | | | | | | | | | | | | | | | | | | | | | | | | | | | | | | | | | | | | | | | | | | | | | | | | | | | | | | | | | | | | | | | | | | | | | | | | | | | | | | | | | | | | | | | | | | | | | | | | | | | | | | | | | | | | | | | | | | | | | |  |
|  |  |  |  |  |  |  |  |  |  |  |  |  |  |  |  |  |  |  |  |  |  |  |  |  |  |  |  |  |  |  |  |  |  |  |  |  |  |  |  |  |  |  |  |  |  |  |  |  |  |  |  |  |  |  |  |  |  |  |  |  |  |  |  |  |  |  |  |  |  |  |  |  |  |  |  |  |  |  |  |  |  |  |  |  |  |  |  |  |  |  |  |  |  |  |  |  | **Cadres** | | | | | | | | | | | | | | | | | | | | | | | | | | | | | | | | | | | | | | | | | **Description** | | | | | | | | | | | | | | | | | | | | | | | | | | | | | | | | | | | | | | | | | | | | | | | | | **Training** | | | | | | | | | | | | | | | | | | | | | | | | | | | | | | | | | | | | | | | | | | | | | | | | | | | | **Supervision** | | | | | | | | | | | | | | | | | | | | | | | | | | | | | | | **Misc.** | |  |
| Three slums in Dhaka, Bangladesh | | | | | | Opportunistic sampling, before and after with questionnaire and observation methods. | | | | | | | | | | | | | | | | | | | | | | | | | | | | | | | | 37 caregivers of children (aged 1-11) with moderate-severe cerebral palsy. | | | | | | | | | | | | | | | | | | | | | | | | | | | | | | | | | | | | Control group made by measuring outcomes of just advice prior to full training for 20 caregiver-child pairs. | | | | | | | | | | | | | | | | | | | | | | | NGO fieldworkers, generic therapists, and caregivers. | | | | | | | | | | | | | | | | | | | | | | | | | | | | | | | | | | | | | | | | | NGO staff identified participants after workshop to inform study occurred, and screening occurred at health centres for inclusion. Enrolled caregiver-child pairs underwent training sessions on feeding children with cerebral palsy, which consisted appropriate foods, consistency, and utensils, as well as feeding methods. | | | | | | | | | | | | | | | | | | | | | | | | | | | | | | | | | | | | | | | | | | | | | | | | | Six fortnightly training sessions run at hospital in groups of 4-5, run by therapists trained in delivering programme. Traditional teaching, discussion, visual aids (including video drama), and participatory and experimental activities used for training. | | | | | | | | | | | | | | | | | | | | | | | | | | | | | | | | | | | | | | | | | | | | | | | | | | | | Training sessions included supervised feedings. Generic therapists that provided training were supervised by first author of paper. | | | | | | | | | | | | | | | | | | | | | | | | | | | | | | | **Quality- 2.5**  MMAT – Quant4 | |  |
| **CMOCs** | | | | | | | | | | | | | | | | | | | | | | | | | | | | | | | | | | | | | | | | | | | | | | | | | | | | | | | | | | | | | | | | | | | | | | | | | | | | | | | | | | | | | | | | | | | | | | | | | | | | | | | | | | | | | | | | | | | | | | | | | | | | | | | | | | | | | | | | | | | | | | | | | | | | | | | | | | | | | | | | | | | | | | | | | | | | | | | | | | | | | | | | | | | | | | | | | | | | | | | | | | | | | | | | | | | | | | | | | | | | | | | | | | | | | | | | | | | | | | | | | | | | | | | | | | | | | | | | | | | | | | | | | | | | | | | |  |
| **Context** | | | | | | | | | | | | | | | | | | | | | | | | | | | | | | | | | | | | | | | | | | | | | | | | | | | | | | | | | | | **Mechanisms** | | | | | | | | | | | | | | | | | | | | | | | | | | | | | | | | | | | | | | | | | | | | | | | | | | | | | | | | | | | | | | | | **Outcomes** | | | | | | | | | | | | | | | | | | | | | | | | | | | | | | | | | | | | | | | | | | | | | | | | | | | | | | | | **CMOCs** | | | | | | | | | | | | | | | | | | | | | | | | | | | | | | | | | | | | | | | | | | | | | | | | | | | | | | | | | | | | | | | | | | | | | | | | | | | | | | | | | | | | | | | | | | | | |  |
| Estimated prevalence of CP in Bangladesh is 22/1000 in children aged 2-9 years. Identified pairs underwent initial training in groups of 4-5 pairs, consisting of 6 fortnightly sessions at the local hospital. Sessions run by generic therapist who received specific training and who was supervised by author.  Training of mothers focused on: appropriate foods; appropriate food consistency; appropriate utensils; appropriate postural and physical support for positioning during feeding and sensitive/responsive manner.  Training for caregivers in feedback practices, including some provision of basic implements like plastic spoons | | | | | | | | | | | | | | | | | | | | | | | | | | | | | | | | | | | | | | | | | | | | | | | | | | | | | | | | | | | 1.Training caregivers to perform daily tasks for children feasible for some disabilities  2. Caregivers’ who recognize decrease in their stress level which may act as incentive to continue training and programme.  3. Acknowledgement of programme working may be incentive for caregivers to continue with training and programme.  4.Training outside of the home should be offered close to home as to ensure attendance.  5. Practical training and demonstration, which incorporates feedback. | | | | | | | | | | | | | | | | | | | | | | | | | | | | | | | | | | | | | | | | | | | | | | | | | | | | | | | | | | | | | | | | Significant improvements in children’s: respiratory health, cooperation during mealtimes, overall mood when mothers had minimum of four sessions.  Reductions in caregivers stress during feeding and length of mealtime.  Carer who received advice and training had higher scores than those who just received advice.  Minimum of 4 training sessions was found to be effective.  Dropout of 13/37 during training because they could not get to the center, usually moving back to the village because of financial difficulties | | | | | | | | | | | | | | | | | | | | | | | | | | | | | | | | | | | | | | | | | | | | | | | | | | | | | | | | 1.Training of families and parents of PWD (especially children) on appropriate and feasible in-home care or rehabilitation techniques is feasible for some services.  2.Training of carers on simple health techniques can reduce stress and improve both health outcomes for PWD and the carer.  3. With little training (a workshop), already working field workers are capable of identifying persons with disabilities and direct to appropriate services  4. Generic therapists can undergo short training of trainers and be effective teachers to caregivers in a specific field.  5. Training should be practical, and if possible involve the supervision of the intervention actively being done.  6. Training location must be sensitive to local context and conditions, especially in the case of multiple training sessions or follow-up. | | | | | | | | | | | | | | | | | | | | | | | | | | | | | | | | | | | | | | | | | | | | | | | | | | | | | | | | | | | | | | | | | | | | | | | | | | | | | | | | | | | | | | | | | | | | |  |
| **Title:** Evaluation of the Community Based Rehabilitation Programme in Uganda | | | | | | | | | | | | | | | | | | | | | | | | | | | | | | | | | | | | | | | | | | | | | | | | | | | | | | | | | | | | | | | | | | | | | | | | | | | | | | | | | | | | | | | | | | | | | | | | | | | | | | | | | | | | | | | | | | | | | | | | | | | | | | | | | | | | | | | | | | | | | | | | | | | | | | | | | | | | | | | | | | | | | | | | | | | | | | | | | | | | | | | | | | | | | | | | | | | | | | | | | | | | | | | | | | | | | | | | | | | | | | | | | | | | | | | | | | | | | | | | | | | | | | | | | | | | | | | | | | | | | | | | | | | | | | | |  |
| **Authors:** Claussen, J., Kandyomunda, B. & Jareg, P. | | | | | | | | | | | | | | | | | | | | | | | | | | | | | | | | | | | | | | | | | | | | | | | | | | | | | | | | | | | | | | | | | | | | | | | | | | | | | | | | | | | | | | | | | | | | | | | | | | | | | | | | | | | | | | | | | | | | | | | | | | | | | | | | | | | | | | | | | | | | | | | | | | | | | | | | | | | | | | | | | | | | | | | | | | | | | | | | | | | | | | | | | | | | | | | | | | | | | | | | | | | | | | | | | | | **Year:** 2005 | | | | | | | | | | | | | | | | | | | | | | | | | | | | | | | | | | | | | | | | | | | | | | | | | | | | | | | | | | | | |  |
| **Summary:** Community lay health workers and supervisors implementing CBR programme in Uganda | | | | | | | | | | | | | | | | | | | | | | | | | | | | | | | | | | | | | | | | | | | | | | | | | | | | | | | | | | | | | | | | | | | | | | | | | | | | | | | | | | | | | | | | | | | | | | | | | | | | | | | | | | | | | | | | | | | | | | | | | | | | | | | | | | | | | | | | | | | | | | | | | | | | | | | | | | | | | | | | | | | | | | | | | | | | | | | | | | | | | | | | | | | | | | | | | | | | | | | | | | | | | | | | | | | | | | | | | | | | | | | | | | | | | | | | | | | | | | | | | | | | | | | | | | | | | | | | | | | | | | | | | | | | | | | |  |
| **Setting** | **Design/**  **Methods** | | | | | | | | | | | | | | | | | | | | **Population** | | | | | | | | | | | | | | | | | | | | | | | | | | | | | | | | **Intervention** | | | | | | | | | | | | | | | | | | | | | | | | | | | | | | | | | | | | | | | | | **Workforce Characteristics** | | | | | | | | | | | | | | | | | | | | | | | | | | | | | | | | | | | | | | | | | | | | | | | | | | | | | | | | | | | | | | | | | | | | | | | | | | | | | | | | | | | | | | | | | | | | | | | | | | | | | | | | | | | | | | | | | | | | | | | | | | | | | | | | | | | | | | | | | | | | | | | | | | | | | | | | | | | | | | | | | | | | | | | | | | | | | | | | | |  |
|  |  |  |  |  |  |  |  |  |  |  |  |  |  |  |  |  |  |  |  |  |  |  |  |  |  |  |  |  |  |  |  |  |  |  |  |  |  |  |  |  |  |  |  |  |  |  |  |  |  |  |  |  |  |  |  |  |  |  |  |  |  |  |  |  |  |  |  |  |  |  |  |  |  |  |  |  |  |  |  |  |  |  |  |  |  |  |  |  |  |  |  |  |  | **Cadres** | | | | | | | | | | | | | | | | | | | | | | | | | | | | | | | | | | | | | | | | | | | | | | | | | | | **Description** | | | | | | | | | | | | | | | | | | | | | | | | | | | | | | | | | | | | | | | | | | | | | | | | | **Training** | | | | | | | | | | | | | | | | | | | | | | | | | | | | | | | | | | | | | | | | | **Supervision** | | | | | | | | | | | | | | | | | | | | | | | | | **Misc.** | | | | | | | | | | | |  |
| Tororo District, Uganda. | External evaluation of CBR programme (mixed methods) | | | | | | | | | | | | | | | | | | | | Persons with Disabilities and their communities. | | | | | | | | | | | | | | | | | | | | | | | | | | | | | | | | Decentralized –subcounties, creating awareness and building capacity at the community level, using volunteers at community level. Next level the District Rehabilitation Office operates, which manages programme and M&E. CBR Steering Committee at district manages, oversees and supervises all. | | | | | | | | | | | | | | | | | | | | | | | | | | | | | | | | | | | | | | | | | Volunteer community based workers, Community Development Officers/Assistants (CDO/As), Health Assistants (HAs) Special Needs Education Coordinators (SNECOs), DPOs. | | | | | | | | | | | | | | | | | | | | | | | | | | | | | | | | | | | | | | | | | | | | | | | | | | | Volunteers are selected by their communities and they work with CDOs, HAS, SNECOs, DPOs. Volunteers identify and assist PWD in communities and raise awareness. Volunteers provide home based activities and interventions, train parents and make simple assistive devices.  PWD receive home visit from volunteer approx. once per month. | | | | | | | | | | | | | | | | | | | | | | | | | | | | | | | | | | | | | | | | | | | | | | | | | Volunteers received two week comprehensive training which covers, range of disabilities, leadership skills, basic counseling skills, mobilization skills, assessment etc. Training mostly theoretical. | | | | | | | | | | | | | | | | | | | | | | | | | | | | | | | | | | | | | | | | | Steering Committees, National CBR Coordinator, local government. High monitoring and supervision at the National and district level. | | | | | | | | | | | | | | | | | | | | | | | | | Volunteers work on average with 100 PWD, with a range from 76-336.  **Quality – NA/**descriptive report | | | | | | | | | | | |  |
| **CMOCs** | | | | | | | | | | | | | | | | | | | | | | | | | | | | | | | | | | | | | | | | | | | | | | | | | | | | | | | | | | | | | | | | | | | | | | | | | | | | | | | | | | | | | | | | | | | | | | | | | | | | | | | | | | | | | | | | | | | | | | | | | | | | | | | | | | | | | | | | | | | | | | | | | | | | | | | | | | | | | | | | | | | | | | | | | | | | | | | | | | | | | | | | | | | | | | | | | | | | | | | | | | | | | | | | | | | | | | | | | | | | | | | | | | | | | | | | | | | | | | | | | | | | | | | | | | | | | | | | | | | | | | | | | | | | | | | |  |
| **Context** | | | | | | | | | | | | | | | | | | | | | | | | | | | | | | | | | | | | | | | | | | | | | | | **Mechanisms** | | | | | | | | | | | | | | | | | | | | | | | | | | | | | | | | | | | | | | | | | | | | | | **Outcomes** | | | | | | | | | | | | | | | | | | | | | | | | | | | | | | | | | | | | | | | | | | | | | | | | | | | | | | | | | | | | | | | | | | | | | | | | | | | | | | | | | | | | | | | | | | | | | | | | | | | | | | | | | | | | | | | | | **CMOCs** | | | | | | | | | | | | | | | | | | | | | | | | | | | | | | | | | | | | | | | | | | | | | | | | | | | | | | | | | | | | | | | | | |  |
| CBR programme in implementation since 2002/2003 – prior to CBR programme there was national level and supply driven model.  Inclusion of stakeholders (DPOs) in planning, execution and monitoring.  CBR Steering committees at sub-county level to facilitate greater participation of local communities.  Volunteers to receive bicycles and allowance for travel.  Volunteers spend approximately 1 day per week on CBR activities, 70% of which are related to ADLs, managing disability and mobilization of PWD.  One CBR workers is trained per parish.  Local artisans trained to carry out minor repairs on assistive devices.  DPOs assist in mobilization of communities and raising awareness. | | | | | | | | | | | | | | | | | | | | | | | | | | | | | | | | | | | | | | | | | | | | | | | 1.Decentralized services which are brought to the community increases identification and assessment of PWD.  2.Volunteers capable of working in communities to assist PWD.  3. Training should be longer than two weeks to have properly functioning CBR workers.  4. Lack of preparedness of health system of respond to increased demand due to programme and CBR workers can influence effectiveness  5. Programmes inability to adhere to mandate/promises to CBR workers may influence motivation/retention | | | | | | | | | | | | | | | | | | | | | | | | | | | | | | | | | | | | | | | | | | | | | | Identification and assessment of over 6500 PWD – higher outreach as opposed to previous model, but still not reaching approx. 50% of PWD.  PWD and families seeing themselves as partners in mobilization not only service users.  Increase in awareness of disability and mobilization of communities in supporting PWD.  Higher number of referrals.  More mainstreaming of services.  Not enough resources for appropriate number of refreshers, low number of volunteers.  Referral system not operating as efficiently as could or as intended, especially for assistive devices and surgery.  Communities and institutions have difficultly with sensory impaired individuals, notably in schools.  Limited number of volunteers have received their bicycles and allowance for home visits.  Training too short to have volunteers be proficient CBR workers. Too little refresher training.  Sign language seen has highly important by many stakeholders.  Identified need for more practical training.  Supervision possibly not coming down to the community level. | | | | | | | | | | | | | | | | | | | | | | | | | | | | | | | | | | | | | | | | | | | | | | | | | | | | | | | | | | | | | | | | | | | | | | | | | | | | | | | | | | | | | | | | | | | | | | | | | | | | | | | | | | | | | | | | | 1.Stakeholders need to be represented from all levels of service, including referral services.  2. Stakeholders (PWD, DPOs, communities) should be involved in the planning, execution and monitoring of programmes and the workforce  3. CBR workers (volunteers) should receive non-financial incentives  4. Situational analysis should occur prior to training workforce and implementing programme  5. Community based workers effective means of identifying PWDs in communities  6. Monitoring of training activities and CBR workers proficiency essential  7. Refresher courses essential for CBR worker’s skills  8. Supervision should be multi-tiered and throughout all programme levels  9. Self-efficacy of CBR workers and supervisors essential for motivation and retention | | | | | | | | | | | | | | | | | | | | | | | | | | | | | | | | | | | | | | | | | | | | | | | | | | | | | | | | | | | | | | | | | |  |
| **Title:** Needs Assessment of Programmes Integrating Community Based Rehabilitation into Health Activities | | | | | | | | | | | | | | | | | | | | | | | | | | | | | | | | | | | | | | | | | | | | | | | | | | | | | | | | | | | | | | | | | | | | | | | | | | | | | | | | | | | | | | | | | | | | | | | | | | | | | | | | | | | | | | | | | | | | | | | | | | | | | | | | | | | | | | | | | | | | | | | | | | | | | | | | | | | | | | | | | | | | | | | | | | | | | | | | | | | | | | | | | | | | | | | | | | | | | | | | | | | | | | | | | | | | | | | | | | | | | | | | | | | | | | | | | | | | | | | | | | | | | | | | | | | | | | | | | | | | | | | | | | | | | | | |  |
| **Authors:** Johnson, RS. & Latha, MP. | | | | | | | | | | | | | | | | | | | | | | | | | | | | | | | | | | | | | | | | | | | | | | | | | | | | | | | | | | | | | | | | | | | | | | | | | | | | | | | | | | | | | | | | | | | | | | | | | | | | | | | | | | | | | | | | | | | | | | | | | | | | | | | | | | | | | | | | | | | | | | | | | | | | | | | | | | | | | | | | | | | | | | | | | | | | | | | | | | | | | | | | | | | | | | | | | | | | | | | | | | | | | | | | | | | **Year:** 2004 | | | | | | | | | | | | | | | | | | | | | | | | | | | | | | | | | | | | | | | | | | | | | | | | | | | | | | | | | | | | |  |
| **Summary:** Study with NGOs and staff involved in health promotion on their resources for and acceptability to integrating community based rehabilitation into programmes. | | | | | | | | | | | | | | | | | | | | | | | | | | | | | | | | | | | | | | | | | | | | | | | | | | | | | | | | | | | | | | | | | | | | | | | | | | | | | | | | | | | | | | | | | | | | | | | | | | | | | | | | | | | | | | | | | | | | | | | | | | | | | | | | | | | | | | | | | | | | | | | | | | | | | | | | | | | | | | | | | | | | | | | | | | | | | | | | | | | | | | | | | | | | | | | | | | | | | | | | | | | | | | | | | | | | | | | | | | | | | | | | | | | | | | | | | | | | | | | | | | | | | | | | | | | | | | | | | | | | | | | | | | | | | | | |  |
| **Setting** | | | | | | **Design/**  **Methods** | | | | | | | | | | | | | | | | | | | | | | | | | | **Population** | | | | | | | | | | | | | | | | | | | | | | | | | | | | | | | | | | | | | | | | | | | | | | | | | | | **Intervention** | | | | | | | | | | | | | | | | | | | | | | | | | | | | | | | | | | | **Workforce Characteristics** | | | | | | | | | | | | | | | | | | | | | | | | | | | | | | | | | | | | | | | | | | | | | | | | | | | | | | | | | | | | | | | | | | | | | | | | | | | | | | | | | | | | | | | | | | | | | | | | | | | | | | | | | | | | | | | | | | | | | | | | | | | | | | | | | | | | | | | | | | | | | | | | | | | | | | | | | |  |
|  |  |  |  |  |  |  |  |  |  |  |  |  |  |  |  |  |  |  |  |  |  |  |  |  |  |  |  |  |  |  |  |  |  |  |  |  |  |  |  |  |  |  |  |  |  |  |  |  |  |  |  |  |  |  |  |  |  |  |  |  |  |  |  |  |  |  |  |  |  |  |  |  |  |  |  |  |  |  |  |  |  |  |  |  |  |  |  |  |  |  |  |  |  |  |  |  |  |  |  |  |  |  |  |  |  |  |  |  |  |  |  |  |  |  |  |  |  | **Cadres** | | | | | | | | | | | | | | | | | | | | | | | | | | | | | | | | | | | | | | | | | **Description** | | | | | | | | | | | | | | | | | | | | | | | | | | | | | | | | | | | **Training** | | | | | | | | | | | | | | | | | | | | | | | | | | | | | | | | | | | | | | | | | | | | | **Supervision** | | | | | | | | | | | | | | | | | | | | | | | | | | **Misc.** | | | | | | |  |
| Tamil Nadu, India. | | | | | | Mixed-method: survey, interviews, focus groups and field observations. | | | | | | | | | | | | | | | | | | | | | | | | | | 176 voluntary, non-governmental organizations (NGOs) for questionnaires. 107 CHWs in focus groups, and 30 chief functionaries had in depth interviews. | | | | | | | | | | | | | | | | | | | | | | | | | | | | | | | | | | | | | | | | | | | | | | | | | | | To assess need and demand among NGOs promoting health to incorporate CBR into their practices, and to identify resources available for working with PWD. | | | | | | | | | | | | | | | | | | | | | | | | | | | | | | | | | | | Community Health Workers (CHWs), chief functionaries | | | | | | | | | | | | | | | | | | | | | | | | | | | | | | | | | | | | | | | | | Would vary between NGO, but 37% chief functionaries had postgraduate degree, 13.6% had PhD, 33.4% had other educational qualification. | | | | | | | | | | | | | | | | | | | | | | | | | | | | | | | | | | | All chief functionaries reported having some training on issues for PWD. | | | | | | | | | | | | | | | | | | | | | | | | | | | | | | | | | | | | | | | | | | | | | Not reported – would vary depending on NGO. | | | | | | | | | | | | | | | | | | | | | | | | | | **Quality -1**  MMAT - MM | | | | | | |  |
| **CMOCs** | | | | | | | | | | | | | | | | | | | | | | | | | | | | | | | | | | | | | | | | | | | | | | | | | | | | | | | | | | | | | | | | | | | | | | | | | | | | | | | | | | | | | | | | | | | | | | | | | | | | | | | | | | | | | | | | | | | | | | | | | | | | | | | | | | | | | | | | | | | | | | | | | | | | | | | | | | | | | | | | | | | | | | | | | | | | | | | | | | | | | | | | | | | | | | | | | | | | | | | | | | | | | | | | | | | | | | | | | | | | | | | | | | | | | | | | | | | | | | | | | | | | | | | | | | | | | | | | | | | | | | | | | | | | | | | |  |
| **Context** | | | | | | | | | | | | | | | | | | | | | | | | | | | | | | | | | | | | | | | | | | | | | | | | | | | | | **Mechanisms** | | | | | | | | | | | | | | | | | | | | | | | | | | | | | | | | | | | | | | | | | | | | | | | | | | | | | | | | | | | | | | | | | | | | | | **Outcomes** | | | | | | | | | | | | | | | | | | | | | | | | | | | | | | | | | | | | | | | | | | | | | | | | | | | | | | | | | | | | | | | | | | | | | | | | | | | | | | | | | | | | | | | | | | | | | | | | | | | | | **CMOCs** | | | | | | | | | | | | | | | | | | | | | | | | | | | | | | | | | | | | | | | | | | | | | | | |  |
| 176 responded NGOs. 5 Focus groups with CHWs from varying organisations totaling 107 participants, and in-depth interviews with 30 chief functionaries of voluntary organisations.  Most respondents were community-based organisations, with only 14% from hospitals and 4% from dispensaries.  Low level of awareness of legal context, specifically Persons with Disability Act 1995, in the local region.  Aware of strategies like educational and vocational training.  Chief functionaries ‘highly’ educated.  All chief functionaries had participated in training programmes on issues relating to disability. | | | | | | | | | | | | | | | | | | | | | | | | | | | | | | | | | | | | | | | | | | | | | | | | | | | | | 1.NGOs and individuals working in communities are open and accepting to the concept of CBR and want more knowledge on how to implement and work with PWD in their communities, however there is limited knowledge on this topic.  2. A holistic approach to CBR, following the CBR Matrix and including prevention, and access to programmes/government schemes and establishment of services including schools etc. important to potential CBR workers.  3. NGOs working in health promotion recognize the importance of disability education and community-based rehabilitation, and require further training to increase knowledge.  4. Community-based rehabilitation knowledge concentrated in managerial positions, or with people with higher education. | | | | | | | | | | | | | | | | | | | | | | | | | | | | | | | | | | | | | | | | | | | | | | | | | | | | | | | | | | | | | | | | | | | | | | 28/176 involved in rehabilitation; 116 interested in CBR; 29 interested in rehabilitation; 3 not interested  Areas of interest identified by community health workers are components of CBR, prevention of disability, establishment of schools/homes, early intervention.  Concept of community based rehabilitation new to 90% of community health workers interviewed.  Training needs identified by CHWs interested in integrating CBR into programmes: basic understanding of disability, early identification and intervention, CBR implementation strategies, and procedures to access government schemes.  Chief functionaries identified need for further rehabilitation information and continuous improvement of knowledge.  Very limited knowledge on Acts for PWD (less than 1% of CHWs).  High (80%) amount of workers interested in learning how to integrated CBR into existing programmes and high (74%) amount of chief functionaries interested in training/orientation for CBR.  95% of them reported a willingness to depute 2-3 staff to CBR service of some kind (including training) | | | | | | | | | | | | | | | | | | | | | | | | | | | | | | | | | | | | | | | | | | | | | | | | | | | | | | | | | | | | | | | | | | | | | | | | | | | | | | | | | | | | | | | | | | | | | | | | | | | | | 1.Currently working CHWs and other staff in NGOs can be trained to incorporate CBR into their current work.  2. NGOs working in health promotion should be trained on resources for persons with disabilities, including but not limited to the policy and legal frameworks working with PWD.  3. CBR workers should be trained on the CBR Matrix, including how PWD can access important rights like employment and education.  4. CBR workers need training on community engagement.  5. As many in more managerial positions are aware of CBR, this knowledge should be shared with community based workers (CHWs), as a means of increasing awareness and knowledge sharing within organizations. | | | | | | | | | | | | | | | | | | | | | | | | | | | | | | | | | | | | | | | | | | | | | | | |  |
| **Title:** Controlled Trial of Psychotherapy for Congolese Survivors of Sexual Violence | | | | | | | | | | | | | | | | | | | | | | | | | | | | | | | | | | | | | | | | | | | | | | | | | | | | | | | | | | | | | | | | | | | | | | | | | | | | | | | | | | | | | | | | | | | | | | | | | | | | | | | | | | | | | | | | | | | | | | | | | | | | | | | | | | | | | | | | | | | | | | | | | | | | | | | | | | | | | | | | | | | | | | | | | | | | | | | | | | | | | | | | | | | | | | | | | | | | | | | | | | | | | | | | | | | | | | | | | | | | | | | | | | | | | | | | | | | | | | | | | | | | | | | | | | | | | | | | | | | | | | | | | | | | | | | |  |
| **Authors:** Bass, JK., Annan, J., McIvor Murray, S., Kaysen, D., Griffiths, S., Cetinoglu, T., Wachter, K., Murray, LK. & Bolton, PA. | | | | | | | | | | | | | | | | | | | | | | | | | | | | | | | | | | | | | | | | | | | | | | | | | | | | | | | | | | | | | | | | | | | | | | | | | | | | | | | | | | | | | | | | | | | | | | | | | | | | | | | | | | | | | | | | | | | | | | | | | | | | | | | | | | | | | | | | | | | | | | | | | | | | | | | | | | | | | | | | | | | | | | | | | | | | | | | | | | | | | | | | | | | | | | | | | | | | | | | | | | | | | | | | | | | **Year:** 2013 | | | | | | | | | | | | | | | | | | | | | | | | | | | | | | | | | | | | | | | | | | | | | | | | | | | | | | | | | | | | |  |
| **Summary:** Evaluation of adaption of group cognitive processing therapy by community based paraprofessionals. | | | | | | | | | | | | | | | | | | | | | | | | | | | | | | | | | | | | | | | | | | | | | | | | | | | | | | | | | | | | | | | | | | | | | | | | | | | | | | | | | | | | | | | | | | | | | | | | | | | | | | | | | | | | | | | | | | | | | | | | | | | | | | | | | | | | | | | | | | | | | | | | | | | | | | | | | | | | | | | | | | | | | | | | | | | | | | | | | | | | | | | | | | | | | | | | | | | | | | | | | | | | | | | | | | | | | | | | | | | | | | | | | | | | | | | | | | | | | | | | | | | | | | | | | | | | | | | | | | | | | | | | | | | | | | | |  |
| **Setting** | | | | | | **Design/Methods** | | | | | | | | | | | | | | | **Population** | | | | | | | | | | | | | | | | | | | | | | | | | | | | | | | | | | | | | | **Intervention** | | | | | | | | | | | | | | | | | | | | | | | | | | | | | | | **Workforce Characteristics** | | | | | | | | | | | | | | | | | | | | | | | | | | | | | | | | | | | | | | | | | | | | | | | | | | | | | | | | | | | | | | | | | | | | | | | | | | | | | | | | | | | | | | | | | | | | | | | | | | | | | | | | | | | | | | | | | | | | | | | | | | | | | | | | | | | | | | | | | | | | | | | | | | | | | | | | | | | | | | | | | | | | | | | | | | | | | | | | | | | | | |  |
|  |  |  |  |  |  |  |  |  |  |  |  |  |  |  |  |  |  |  |  |  |  |  |  |  |  |  |  |  |  |  |  |  |  |  |  |  |  |  |  |  |  |  |  |  |  |  |  |  |  |  |  |  |  |  |  |  |  |  |  |  |  |  |  |  |  |  |  |  |  |  |  |  |  |  |  |  |  |  |  |  |  |  |  |  |  |  |  |  |  | **Cadres** | | | | | | | | | | | | | | | | | | | | | | | | | | **Description** | | | | | | | | | | | | | | | | | | | | | | | | | | | | | | | | | | | | | | | | | | | | | | **Training** | | | | | | | | | | | | | | | | | | | | | | | | | **Supervision** | | | | | | | | | | | | | | | | | | | | | | | | | | | | | | | | | | | | | | | | | | | | | | | | | | | | | | **Misc.** | | | | | | | | | | | | | | | | | | | | | | | | | | | | | | |  |
| 14 villages in South Kivu province, 2 villages in North Kivu Province, Democratic Republic of the Congo. | | | | | | Mixed-methods | | | | | | | | | | | | | | | Female sexual-violence survivors with high levels of PTSD symptoms and combined depression and anxiety symptoms. | | | | | | | | | | | | | | | | | | | | | | | | | | | | | | | | | | | | | | 16 villages randomly assigned to either cognitive processing therapy (1 individual session and 11 group sessions, n=157) or individual support (n=248) to female sexual-violence survivors | | | | | | | | | | | | | | | | | | | | | | | | | | | | | | | Psychosocial assistants | | | | | | | | | | | | | | | | | | | | | | | | | | Community-based paraprofessionals. 1-9 years of experience in case management and individual supportive counseling to survivors of sexual violence. Minimum 4 years of post-primary education. Psychosocial assistants based in the same village. | | | | | | | | | | | | | | | | | | | | | | | | | | | | | | | | | | | | | | | | | | | | | | 5-6 day training session conducted by International Rescue Committee (IRC). Psychosocial assistants providing therapy had 2 weeks in-person training with international trainers. | | | | | | | | | | | | | | | | | | | | | | | | | Multi-tiered system, with Congolese psychosocial supervisors (IRC employees) providing direct supervision via weekly in-person on phone meetings, clinical social worker (trained internationally) for in-country support and communication with US trainers through weekly calls for quality insurance. | | | | | | | | | | | | | | | | | | | | | | | | | | | | | | | | | | | | | | | | | | | | | | | | | | | | | | Adherence to therapy protocol assessed by checklists of key treatment elements, as observed by supervisors at group sessions. Each psychosocial assistant lead 3 therapy groups at a time, maximum 8 women per group.  **Quality: 3**  MMAT - Qualitative | | | | | | | | | | | | | | | | | | | | | | | | | | | | | | |  |
| **CMOCs** | | | | | | | | | | | | | | | | | | | | | | | | | | | | | | | | | | | | | | | | | | | | | | | | | | | | | | | | | | | | | | | | | | | | | | | | | | | | | | | | | | | | | | | | | | | | | | | | | | | | | | | | | | | | | | | | | | | | | | | | | | | | | | | | | | | | | | | | | | | | | | | | | | | | | | | | | | | | | | | | | | | | | | | | | | | | | | | | | | | | | | | | | | | | | | | | | | | | | | | | | | | | | | | | | | | | | | | | | | | | | | | | | | | | | | | | | | | | | | | | | | | | | | | | | | | | | | | | | | | | | | | | | | | | | | | |  |
| **Context** | | | | | | | | | | | | | | | | | | | | | | | | | | | | | | | | | | | | | | | | | | | | | | | | | | | | | | | | | | | | | | | | | | | | | | | | | | | | | | | | | | | **Mechanisms** | | | | | | | | | | | | | | | | | | | | | | | | | | | | | | | | | | | | | | | | | | | | | | | | | | | | | | | | | | | | | | | | | | | | | | | | | | | | | | | **Outcomes** | | | | | | | | | | | | | | | | | | | | | | | | | | | | | | | | | | | | | | | | | | | | | | | | | | | | | | | | | | | | | | **CMOCs** | | | | | | | | | | | | | | | | | | | | | | | | | | | | | | | | | | | | | | | | | | | | | | | |  |
| 40% of women in area have experiences some form of sexual violence.  Psychosocial assistants were given quizzes and observed, if competency was questioned they were not included.  15 villages (7 provided therapy and 8 provided individual support), with a psychosocial assistant in each one. Assistants recruited up to 24 participants, and a maximum of 8 women per treatment group. In villages with individuals support, no limits on participants were given.  Intervention lasted around 4 months. Follow-up data collected within 1 month of treatment completion and at 6-month follow-up.  Cognitive therapy – 1 individual session lasting 1 hour, and 11 sessions with 6-8 women lasting 2 hours. Participants had access to assistant outside of group.  Supervised by psychosocial staff at an NGO, and expat clinical experts.  Topics for training include: case management, counseling, mediation, stress management, prevention of HIV and other STIs and clinical care. | | | | | | | | | | | | | | | | | | | | | | | | | | | | | | | | | | | | | | | | | | | | | | | | | | | | | | | | | | | | | | | | | | | | | | | | | | | | | | | | | | | 1.Lay trained psychosocial assistants (min 9 years experience and 4 years post-primary schooling) can assist in medication adherence, group therapy and individual therapy.  2.Lay trained (with experience) psychosocial assistants effective in improving anxiety, depression, and functional-impairment.  3.Training by experts (including international in this case) in an additional 2-week psychosocial therapy (on top of additional 5-6 days).  4..Strong supportive and training mechanisms (both internal and external) combined with lay health workers from the villages in which they work.  5.Adding of “cognitive processing therapy” compared to benefits of services offered by workers trained only in case management and individual supportive counseling.  6.Relateability of women to her counselor (due to being from same village), may have assisted in acceptance of intervention. | | | | | | | | | | | | | | | | | | | | | | | | | | | | | | | | | | | | | | | | | | | | | | | | | | | | | | | | | | | | | | | | | | | | | | | | | | | | | | | Participants in Individual and Cognitive Group Therapy had significant improvements during treatment (end of treatment assessment) that was maintained at 6 months.  Group therapy had significant improvements over individual therapy at end of treatment and 6mth follow up, with all treatment effect sizes greater than 1.0 for:  HSCL-25 score for combined depression and anxiety;  PTSD Checklist scores:  Functional-impairment scores:  Probable depression or anxiety (no/total no for %);  Probably PTSD (no/total no for %).  One psychosocial assistant who underwent training was not used as her competency to delivery service was in question (due to quizzes and observation). | | | | | | | | | | | | | | | | | | | | | | | | | | | | | | | | | | | | | | | | | | | | | | | | | | | | | | | | | | | | | | 1.Supervision structures should be multi-tiered, and having supervisors themselves be supervised.  2.Supervision should be frequent (1/week), and involve observation of adherence to protocol.  3. Community based workers should be from the communities in which they serve, especially for sensitive rehabilitation topics.  4. International experts can assist in the training and supervision of activities of community based rehabilitation, but services should be offered by individuals close to the community.  5. Paraprofessional workers are capable of providing rehabilitation services for mental health | | | | | | | | | | | | | | | | | | | | | | | | | | | | | | | | | | | | | | | | | | | | | | | |  |
| **Title:** Evaluation de la Réadaptation à Base Communautaire (RBC) au Ghana et au Bénin | | | | | | | | | | | | | | | | | | | | | | | | | | | | | | | | | | | | | | | | | | | | | | | | | | | | | | | | | | | | | | | | | | | | | | | | | | | | | | | | | | | | | | | | | | | | | | | | | | | | | | | | | | | | | | | | | | | | | | | | | | | | | | | | | | | | | | | | | | | | | | | | | | | | | | | | | | | | | | | | | | | | | | | | | | | | | | | | | | | | | | | | | | | | | | | | | | | | | | | | | | | | | | | | | | | | | | | | | | | | | | | | | | | | | | | | | | | | | | | | | | | | | | | | | | | | | | | | | | | | | | | | | | | | | | | |  |
| **Authors:** Jadin, O., Agbogbe, N. & Barima, O. | | | | | | | | | | | | | | | | | | | | | | | | | | | | | | | | | | | | | | | | | | | | | | | | | | | | | | | | | | | | | | | | | | | | | | | | | | | | | | | | | | | | | | | | | | | | | | | | | | | | | | | | | | | | | | | | | | | | | | | | | | | | | | | | | | | | | | | | | | | | | | | | | | | | | | | | | | | | | | | | | | | | | | | | | | | | | | | | | | | | | | | | | | | | | | | | | | | | | | | | | | | | | | | | | | | **Year:** 2005 | | | | | | | | | | | | | | | | | | | | | | | | | | | | | | | | | | | | | | | | | | | | | | | | | | | | | | | | | | | | |  |
| **Summary:** Describes CBR programme run by lay health workers in two countries will differing contexts | | | | | | | | | | | | | | | | | | | | | | | | | | | | | | | | | | | | | | | | | | | | | | | | | | | | | | | | | | | | | | | | | | | | | | | | | | | | | | | | | | | | | | | | | | | | | | | | | | | | | | | | | | | | | | | | | | | | | | | | | | | | | | | | | | | | | | | | | | | | | | | | | | | | | | | | | | | | | | | | | | | | | | | | | | | | | | | | | | | | | | | | | | | | | | | | | | | | | | | | | | | | | | | | | | | | | | | | | | | | | | | | | | | | | | | | | | | | | | | | | | | | | | | | | | | | | | | | | | | | | | | | | | | | | | | |  |
| **Setting** | | | | | | **Design/**  **Methods** | | | | | | | | | | | | | | | | | | | | | | | | | | | | | | | | **Population** | | | | | | | | | | | | | | | | | | | | | | | | | | | | | | | | | | | | | | | | | | | | | | | | | **Intervention** | | | | | | | | | | | | | | | | | | | | | | | | | | | | | | | | | | | | | **Workforce Characteristics** | | | | | | | | | | | | | | | | | | | | | | | | | | | | | | | | | | | | | | | | | | | | | | | | | | | | | | | | | | | | | | | | | | | | | | | | | | | | | | | | | | | | | | | | | | | | | | | | | | | | | | | | | | | | | | | | | | | | | | | | | | | | | | | | | | | | | | | | | | | | | | | | | | | |  |
|  |  |  |  |  |  |  |  |  |  |  |  |  |  |  |  |  |  |  |  |  |  |  |  |  |  |  |  |  |  |  |  |  |  |  |  |  |  |  |  |  |  |  |  |  |  |  |  |  |  |  |  |  |  |  |  |  |  |  |  |  |  |  |  |  |  |  |  |  |  |  |  |  |  |  |  |  |  |  |  |  |  |  |  |  |  |  |  |  |  |  |  |  |  |  |  |  |  |  |  |  |  |  |  |  |  |  |  |  |  |  |  |  |  |  |  |  |  |  |  |  |  |  |  | **Cadres** | | | | | | | | | | | | | | | | | | | | | | | | | | | | | | | | **Description** | | | | | | | | | | | | | | | | | | | | | | | | | | | | | | | | | **Training** | | | | | | | | | | | | | | | | | | | | | | | | | | | | | | | | | | | | | | | | | | | | | | | | | | | | | | **Supervision** | | | | | | | | | | | | | | | | | | | | | | | | | | | | **Misc.** |  |
| Ghana and Benin. | | | | | | Experimental CBR programs  Functional unit is the CBR district; rural or urban; population range of 50-100 000 inhabitants  Ghana  20 CBR districts (19 rural and 1 urban)  Benin  15 CBR districts (9 rural and 6 urban) | | | | | | | | | | | | | | | | | | | | | | | | | | | | | | | | Ghana:  200 000 across 20 CBR districts; no selection criteria for participants  Benin:  1 000 000 across 15 CBR districts; participants selected on the basis of age, type and severity of disability | | | | | | | | | | | | | | | | | | | | | | | | | | | | | | | | | | | | | | | | | | | | | | | | | CBR programs designed in line with WHO recommendations for CBR  CBR district activities are coordinated at the national level.  Local committees manage decentralized CBR districts.  Local committees are composed of voluntary community members. | | | | | | | | | | | | | | | | | | | | | | | | | | | | | | | | | | | | | 1.Intermediary level supervisors;  2. Local facilitators;  3.Family trainer | | | | | | | | | | | | | | | | | | | | | | | | | | | | | | | | Supervisors are appointed at the national level.  Cadre 1 recruits cadre 2.  Cadre 2 is voluntary.  Cadre 1 acts a technical adviser to the local committee.  Cadre 1 coordinates passage to referral services outside the remit of CBR. | | | | | | | | | | | | | | | | | | | | | | | | | | | | | | | | | Cadre 1 trains cadre 2 in the techniques and principles of community based rehabilitation as developed by the WHO.  Cadre 2 visits PH and trains a family member in CBR techniques and principles.  No indication of time and quality of training, | | | | | | | | | | | | | | | | | | | | | | | | | | | | | | | | | | | | | | | | | | | | | | | | | | | | | | Cadre 1 supervises cadre 2.  No indication on quality of supervision.  **Quality – NA**  Descriptive case studies report | | | | | | | | | | | | | | | | | | | | | | | | | | | |  |  |
| **CMOCs** | | | | | | | | | | | | | | | | | | | | | | | | | | | | | | | | | | | | | | | | | | | | | | | | | | | | | | | | | | | | | | | | | | | | | | | | | | | | | | | | | | | | | | | | | | | | | | | | | | | | | | | | | | | | | | | | | | | | | | | | | | | | | | | | | | | | | | | | | | | | | | | | | | | | | | | | | | | | | | | | | | | | | | | | | | | | | | | | | | | | | | | | | | | | | | | | | | | | | | | | | | | | | | | | | | | | | | | | | | | | | | | | | | | | | | | | | | | | | | | | | | | | | | | | | | | | | | | | | | | | | | | | | | | | | | | |  |
| **Context** | | | | | | | | | | | | | | | | | | | | | | | | | | | | | | | | | | **Mechanisms** | | | | | | | | | | | | | | | | | | | | | | | | | | | | | | | | | | | | | | | | | | | | | | | | | | | | | | | | | | | | | | | | | | | | | | | | | | | | | | | | | | | | | | | | | | | | **Outcomes** | | | | | | | | | | | | | | | | | | | | | | | | | | | | | | | | | | | | | | | | | | | | | | | | | | | | | | **CMOCs** | | | | | | | | | | | | | | | | | | | | | | | | | | | | | | | | | | | | | | | | | | | | | | | | | | | | | | | | | | | | | | | | | | | | | | | | | | | | | | | | | | | | | | | | | | | |  |
| Ghana  Overwhelmingly rural; small communities of 2000; sub-districts; multiple CBR committees. Centralized service.  Benin  Urban and rural; larger communities; no-sub districts; single CBR committee. Decentralized service, more community ethos, self-organization of groups, and supported by professional services. | | | | | | | | | | | | | | | | | | | | | | | | | | | | | | | | | | Ghana  Lack of support from intermediary and national level; little fund-raising by local committees; inadequate supervision of local facilitators; local facilitators lack motivation; large amounts of funding allocated to cover costs of national coordination; little financial support for supervisors, facilitators and communities; little or no contact between intermediary level supervisors and national coordination; no financial compensation for local facilitators; no facilitation for referrals to specialized services  Benin  Better support from intermediary and national level; higher levels of fund-raising by local committees; better communication between the three action levels; better allocation of funds between various action levels; financial support for supervisors, facilitators and communities; monthly financial compensation for local facilitators; better facilitation for referrals to specialized services | | | | | | | | | | | | | | | | | | | | | | | | | | | | | | | | | | | | | | | | | | | | | | | | | | | | | | | | | | | | | | | | | | | | | | | | | | | | | | | | | | | | | | | | | | | | Quality of life of PWD from CBR worker and caregiver perspective.  Sustainability of programme: Ghana lost programme in 20 districts, Benin expanded from 15-31.  Better and easier access to referral services in Benin.  CBR program in Benin more cost effective than in Ghana.  Ghanaian program deemed ineffective and inefficient in comparison to program in Benin.  Higher proportion of successful intervention in eyes of CBR worker and caregiver in Benin. Central control had CBR workers feeling less supportive and demotivated. | | | | | | | | | | | | | | | | | | | | | | | | | | | | | | | | | | | | | | | | | | | | | | | | | | | | | | 1.Systemic, decentralization and empowerment at the local community level.  2. Motivated CBR workers, who must feel supported by and through supervision and by central government with resources when and as needed.  3. Community supported at district level, community meetings and forums to address discrimination and self-mobilization.  4. Decentralized workforce more sustainable than centralized workforce  5.Supportive supervision of community based workers essential for motivation and retention of workers  6. CBR workers and supervisors require financial compensation and or expenses covered for retention  7. CBR workers require clear pathways and appropriate training for referrals to more specialized services | | | | | | | | | | | | | | | | | | | | | | | | | | | | | | | | | | | | | | | | | | | | | | | | | | | | | | | | | | | | | | | | | | | | | | | | | | | | | | | | | | | | | | | | | | | |  |
| **Title:** Challenges and opportunities in developing a psychosocial intervention for perinatal depression in rural Pakistan – a multi-method study | | | | | | | | | | | | | | | | | | | | | | | | | | | | | | | | | | | | | | | | | | | | | | | | | | | | | | | | | | | | | | | | | | | | | | | | | | | | | | | | | | | | | | | | | | | | | | | | | | | | | | | | | | | | | | | | | | | | | | | | | | | | | | | | | | | | | | | | | | | | | | | | | | | | | | | | | | | | | | | | | | | | | | | | | | | | | | | | | | | | | | | | | | | | | | | | | | | | | | | | | | | | | | | | | | | | | | | | | | | | | | | | | | | | | | | | | | | | | | | | | | | | | | | | | | | | | | | | | | | | | | | | | | | | | | | |  |
| **Authors:** Rahman, A. | | | | | | | | | | | | | | | | | | | | | | | | | | | | | | | | | | | | | | | | | | | | | | | | | | | | | | | | | | | | | | | | | | | | | | | | | | | | | | | | | | | | | | | | | | | | | | | | | | | | | | | | | | | | | | | | | | | | | | | | | | | | | | | | | | | | | | | | | | | | | | | | | | | | | | | | | | | | | | | | | | | | | | | | | | | | | | | | | | | | | | | | | | | | | | | | | | | | | | | | | | | | | | | | | | **Year:** 2007 | | | | | | | | | | | | | | | | | | | | | | | | | | | | | | | | | | | | | | | | | | | | | | | | | | | | | | | | | | | | | |  |
| **Summary:** Investigation with health workers and programme participants to investigate challenges and opportunities for community health workers to delivery mental health intervention | | | | | | | | | | | | | | | | | | | | | | | | | | | | | | | | | | | | | | | | | | | | | | | | | | | | | | | | | | | | | | | | | | | | | | | | | | | | | | | | | | | | | | | | | | | | | | | | | | | | | | | | | | | | | | | | | | | | | | | | | | | | | | | | | | | | | | | | | | | | | | | | | | | | | | | | | | | | | | | | | | | | | | | | | | | | | | | | | | | | | | | | | | | | | | | | | | | | | | | | | | | | | | | | | | | | | | | | | | | | | | | | | | | | | | | | | | | | | | | | | | | | | | | | | | | | | | | | | | | | | | | | | | | | | | | |  |
| **Setting** | | | | | | | **Design/**  **Methods** | | | | | | | | | | | | | | | | | | **Population** | | | | | | | | | | | | | | | | | | | | | | | | | | | | | | | | | | | | | | | | | | | | | | | | | | | | | | | | | | | | | | | | **Intervention** | | | | | | | | | | | | | | | | | | | | | | | | | | | | | | | | | **Workforce Characteristics** | | | | | | | | | | | | | | | | | | | | | | | | | | | | | | | | | | | | | | | | | | | | | | | | | | | | | | | | | | | | | | | | | | | | | | | | | | | | | | | | | | | | | | | | | | | | | | | | | | | | | | | | | | | | | | | | | | | | | | | | | | | | | | | | | | | | | | | | | | | | | | | | | | | | | |  |
|  | | | | | | |  | | | | | | | | | | | | | | | | | |  | | | | | | | | | | | | | | | | | | | | | | | | | | | | | | | | | | | | | | | | | | | | | | | | | | | | | | | | | | | | | | | |  | | | | | | | | | | | | | | | | | | | | | | | | | | | | | | | | | **Cadres** | | | | | | | | | | | | | | | | | | | | | | | | | | | | | | **Description** | | | | | | | | | | | | | | | | | | | | | | | | | | | | | | | | | | | | **Training** | | | | | | | | | | | | | | | | | | | | | | | | | | | | | | | | | | | | | | | | | | | | | | | | | **Supervision** | | | | | | | | | | | | | | | | | | | | | | | | | | | | | | | | | **Misc.** | |  |
| District of Rawalpindi, Pakistan. | | | | | | | Mixed-methods, formative research. In-depth interviews. | | | | | | | | | | | | | | | | | | Perinatal depressed mothers with low socioeconomic status, and Focus groups with a total of 24 LHWs exploring their experience providing health care to women and difficulties they face. This was used to explore how the intervention would fit within the primary and traditional health care system. | | | | | | | | | | | | | | | | | | | | | | | | | | | | | | | | | | | | | | | | | | | | | | | | | | | | | | | | | | | | | | | | Women diagnosed by trained psychiatrists. LHWs visit women in their areas provide cognitive based therapy. This study is from feedback from LHWs on the training and intervention. | | | | | | | | | | | | | | | | | | | | | | | | | | | | | | | | | Lady Health Workers (LHWs) | | | | | | | | | | | | | | | | | | | | | | | | | | | | | | Lady Health Workers – a formal sector within the Pakistani health systems providing basic primary health care at the village level – are members of the community, have completed high school, and trained on preventive mother and child health care and education. Each Lady Health Worker is responsible for a population of about 1000. | | | | | | | | | | | | | | | | | | | | | | | | | | | | | | | | | | | | Initial 2-day workshop, and a 1-day refresher three months after the initial training. Training includes video with actors conducting sessions, discussions and role-plays. Emphasis on active-listening, additional training on dealing with difficult situations and use of visuals to address literacy. | | | | | | | | | | | | | | | | | | | | | | | | | | | | | | | | | | | | | | | | | | | | | | | | | LHWs supervised monthly in groups of 10 by a public health expert and mental health professional. Supervision includes discussion of problems, and collaborative problem-solving, and sharing experiences. | | | | | | | | | | | | | | | | | | | | | | | | | | | | | | | | | **Quality:**  **4**  MMAT: Qualitative | |  |
| **CMOCs** | | | | | | | | | | | | | | | | | | | | | | | | | | | | | | | | | | | | | | | | | | | | | | | | | | | | | | | | | | | | | | | | | | | | | | | | | | | | | | | | | | | | | | | | | | | | | | | | | | | | | | | | | | | | | | | | | | | | | | | | | | | | | | | | | | | | | | | | | | | | | | | | | | | | | | | | | | | | | | | | | | | | | | | | | | | | | | | | | | | | | | | | | | | | | | | | | | | | | | | | | | | | | | | | | | | | | | | | | | | | | | | | | | | | | | | | | | | | | | | | | | | | | | | | | | | | | | | | | | | | | | | | | | | | | | | |  |
| **Context** | | | | | | | | | | | | | | | | | | | | | | | | | | | | | | | | | | | | | | | | | | | | | | | | | | | | **Mechanisms** | | | | | | | | | | | | | | | | | | | | | | | | | | | | | | | | | | | | | | | | **Outcomes** | | | | | | | | | | | | | | | | | | | | | | | | | | | | | | | | | | | | | | | | | | | | | | | | | | | | | | | | | | | | | | | | | | | | | | | | | | | | | | | | | | | | | | | | | | | | | | | | | | | | | | | | | | | | | | | | | | | | | | | | | | | | | | | **CMOCs** | | | | | | | | | | | | | | | | | | | | | | | | | | | | | | | | | | | | | | | | | | | | | | | | | | | | |  |
| Basic Health Units have one doctor, midwife, vaccinator and between 15-20 Lady Health Workers. In the district, there are no psychologists in the public sector and 3 psychiatrists, based only in Rawalpindi city.  Vast majority of depressions remain undetected and untreated in the local community.  High stigma surrounding mental illness and low levels of awareness. Majority of mental illness goes undiagnosed.  42 LHWs trained to deliver adapted CBT programme to women in their respective home areas.  Intervention focused on mother and all family/household, and was adapted to be culturally sensitive.  Family members given ‘homework’ to help practice more healthy thinking. | | | | | | | | | | | | | | | | | | | | | | | | | | | | | | | | | | | | | | | | | | | | | | | | | | | | 1.LHWs relatable to women and families and mothers may be more accepting.  2.Family members should be involved in programme.  Services worked in a multi-level, where specialized clinicians diagnosed participants, and then community-based workers ran interventions.  Community based workers already integrated into currently health care system (in this case LHWs) are highly accepting to additional work that CBR would include. | | | | | | | | | | | | | | | | | | | | | | | | | | | | | | | | | | | | | | | | Intervention needs to include families as well as mothers.  Some women felt stigmatized; as well some did not recognize depression as a problem.  LHW.  Programme should be integrated into existing LHW training and workload, as to not be seen as an extra burden due to existing heavy workload. Their programme should be simple and easy to follow, and be aware of the stigmatization that is attached to mental illness in the area.  Health staff stated should be called ‘training’ not ‘therapy’, to avoid or help avoid stigma, and emphasize the activity of people.  Due to stigma associated with mental illness, it was found to be important that such interventions are delivered at the home – and due to their already community-based role, LHWs seemed to be appropriate for this intervention.  Should be easy to follow by non-literate clients, and also culturally appropriate.  High acknowledgment that the training is relevant to LHWs work.  LHWs reported that they understand the concepts explained during the training, and that they are able to communicate these concepts to mothers that they visit.  At health system level, concerns around LHW attrition, selection and motivation are acknowledged. | | | | | | | | | | | | | | | | | | | | | | | | | | | | | | | | | | | | | | | | | | | | | | | | | | | | | | | | | | | | | | | | | | | | | | | | | | | | | | | | | | | | | | | | | | | | | | | | | | | | | | | | | | | | | | | | | | | | | | | | | | | | | | | 1.Community based workers, especially when dealing with sensitive topics such as mental health, should be relatable to their clients (i.e. women working with women).  2.Community based workers should have training to help reduce stigma of certain health issues, within individuals, families and communities.  3.Rehabilitation services should be integrated into a worker’s current role and not be run as parallel services, as many already have heavy burden of workload.  4.Mechanisms for training feedback from community based health workers should be implemented into programmes.  5.Pre-existing community-based workers can be trained on rehabilitation techniques to incorporate into their pre-existing job description | | | | | | | | | | | | | | | | | | | | | | | | | | | | | | | | | | | | | | | | | | | | | | | | | | | | |  |
| **Title:** Final Evaluation of a Community Based Rehabilitation Program: A Report | | | | | | | | | | | | | | | | | | | | | | | | | | | | | | | | | | | | | | | | | | | | | | | | | | | | | | | | | | | | | | | | | | | | | | | | | | | | | | | | | | | | | | | | | | | | | | | | | | | | | | | | | | | | | | | | | | | | | | | | | | | | | | | | | | | | | | | | | | | | | | | | | | | | | | | | | | | | | | | | | | | | | | | | | | | | | | | | | | | | | | | | | | | | | | | | | | | | | | | | | | | | | | | | | | | | | | | | | | | | | | | | | | | | | | | | | | | | | | | | | | | | | | | | | | | | | | | | | | | | | | | | | | | | | | | |  |
| **Authors:** Save the Children | | | | | | | | | | | | | | | | | | | | | | | | | | | | | | | | | | | | | | | | | | | | | | | | | | | | | | | | | | | | | | | | | | | | | | | | | | | | | | | | | | | | | | | | | | | | | | | | | | | | | | | | | | | | | | | | | | | | | | | | | | | | | | | | | | | | | | | | | | | | | | | | | | | | | | | | | | | | | | | | | | | | | | | | | | | | | | | | | | | | | | | | | | | | | | | | | | | | | | | | | | | | | | | | | | | | **Year:** 2010 | | | | | | | | | | | | | | | | | | | | | | | | | | | | | | | | | | | | | | | | | | | | | | | | | | | | | | | | | | | |  |
| **Summary:** Final evaluation report of a partnership programme in Nepal using CBR Matrix activities | | | | | | | | | | | | | | | | | | | | | | | | | | | | | | | | | | | | | | | | | | | | | | | | | | | | | | | | | | | | | | | | | | | | | | | | | | | | | | | | | | | | | | | | | | | | | | | | | | | | | | | | | | | | | | | | | | | | | | | | | | | | | | | | | | | | | | | | | | | | | | | | | | | | | | | | | | | | | | | | | | | | | | | | | | | | | | | | | | | | | | | | | | | | | | | | | | | | | | | | | | | | | | | | | | | | | | | | | | | | | | | | | | | | | | | | | | | | | | | | | | | | | | | | | | | | | | | | | | | | | | | | | | | | | | | |  |
| **Setting** | | | | | | | **Design/**  **Methods** | | | | | | | | | | | | | | | | | | | | | | | | | | | | | | | | **Population** | | | | | | | | | | | | | | | | | | | | | | | | | | | | | | | | | **Intervention** | | | | | | | | | | | | | | | | | | | | | | | | | | | | | | | | **Workforce Characteristics** | | | | | | | | | | | | | | | | | | | | | | | | | | | | | | | | | | | | | | | | | | | | | | | | | | | | | | | | | | | | | | | | | | | | | | | | | | | | | | | | | | | | | | | | | | | | | | | | | | | | | | | | | | | | | | | | | | | | | | | | | | | | | | | | | | | | | | | | | | | | | | | | | | | | | | | | | | | | | | | | | | | | | | | |  |
|  |  |  |  |  |  |  |  |  |  |  |  |  |  |  |  |  |  |  |  |  |  |  |  |  |  |  |  |  |  |  |  |  |  |  |  |  |  |  |  |  |  |  |  |  |  |  |  |  |  |  |  |  |  |  |  |  |  |  |  |  |  |  |  |  |  |  |  |  |  |  |  |  |  |  |  |  |  |  |  |  |  |  |  |  |  |  |  |  |  |  |  |  |  |  |  |  |  |  |  |  |  |  |  | **Cadres** | | | | | | | | | | | | | | | | | | | | | | | | | | | | | | | | | | | | | | | | | | | | | **Description** | | | | | | | | | | | | | | | | | | | | | | | | | **Training** | | | | | | | | | | | | | | | | | | | | | | | | | | | | | | | | | | | | | | | | | | | | | | | | | | | | | | | | | | | | | | | | | | **Supervision** | | | | | | | | | | | | | | | | | | | | | | | **Misc.** | | | | | | | | |  |
| Nepal, 7 Municipalities in Eastern and Central | | | | | | | Key informant interviews, focus groups, field observations.  Final Programme Evaluation | | | | | | | | | | | | | | | | | | | | | | | | | | | | | | | | Child and youth with disabilities and their families | | | | | | | | | | | | | | | | | | | | | | | | | | | | | | | | | Focus on partnership, for integrated programme involving advocacy and empowerment, capacity development of organizations, increased access to appropriate services. Includes educational, livelihoods, health | | | | | | | | | | | | | | | | | | | | | | | | | | | | | | | | Village rehabilitation facilitators, CBR coordinators, therapists | | | | | | | | | | | | | | | | | | | | | | | | | | | | | | | | | | | | | | | | | | | | | Differs depending on the partner organization. Involved in home visits, referrals and early identification | | | | | | | | | | | | | | | | | | | | | | | | | CBR capacity development training (100 days) for community workers and therapists. Disability specific on intellectual disability, Portage training, CP training, disability awareness, physiotherapy training, organizational management training, basic Nepali Sign Language | | | | | | | | | | | | | | | | | | | | | | | | | | | | | | | | | | | | | | | | | | | | | | | | | | | | | | | | | | | | | | | | | | Depends on partner organization | | | | | | | | | | | | | | | | | | | | | | | **Quality: 2**  MMAT – Qualitative | | | | | | | | |  |
| **CMOCs** | | | | | | | | | | | | | | | | | | | | | | | | | | | | | | | | | | | | | | | | | | | | | | | | | | | | | | | | | | | | | | | | | | | | | | | | | | | | | | | | | | | | | | | | | | | | | | | | | | | | | | | | | | | | | | | | | | | | | | | | | | | | | | | | | | | | | | | | | | | | | | | | | | | | | | | | | | | | | | | | | | | | | | | | | | | | | | | | | | | | | | | | | | | | | | | | | | | | | | | | | | | | | | | | | | | | | | | | | | | | | | | | | | | | | | | | | | | | | | | | | | | | | | | | | | | | | | | | | | | | | | | | | | | | | | | |  |
| **Context** | | | | | | | | | | | | | | | | | | | | | | | | | | | | | | | | | | | | | | | | | | | | | | | | | | | | | | | | | **Mechanisms** | | | | | | | | | | | | | | | | | | | | | | | | | | | | | | | | | | | | | | | | | | | | **Outcomes** | | | | | | | | | | | | | | | | | | | | | | | | | | | | | | | | | | | | | | | | | | | | | | | | | | | | | | | | | | | | | | | | | | | | | | | | | | | | | | | | | | | | | | | | | **CMOCs** | | | | | | | | | | | | | | | | | | | | | | | | | | | | | | | | | | | | | | | | | | | | | | | | | | | | | | | | | | | | | | | | | | | | | | | | | | | | | | | | | |  |
| Save the Children began supporting CBR activities in 1990.  10% of people in area with some form of disability.  Multiple partnership approach with variety of stakeholders, depending on district.  Child rights base approach to CBR, including using CBR Matrix approach.  Lobbying government and organizations for increased services central component of programme.  Community based rehabilitation  Village Disability Rehabilitation Committees established. | | | | | | | | | | | | | | | | | | | | | | | | | | | | | | | | | | | | | | | | | | | | | | | | | | | | | | | | | 1.Inclusive rehabilitation activities may increase acceptability of families towards programmes  2. Community ownership important for reducing stigma and increasing acceptability of persons with disabilities  3.Bringing services to the home important for ensuring access | | | | | | | | | | | | | | | | | | | | | | | | | | | | | | | | | | | | | | | | | | | | VDRCs recognized as committees ensuring rights of PWD.  Increased involvement of deaf children and those with intellectual disabilities in school in several districts.  Disability scholarships and allowance.  Reduced stigma by community and increased social awareness towards disability.  Reports of improved mobility and access to therapy services.  Increased participation in organizations and clubs.  More supportive attitudes from families towards PWD.  35000 children increased functional capabilities; 10000 children with disabilities in education; 50000 benefited from prevention and early detection activities.  Home visits most popular programme and stakeholders identify its sustainable impact on children.  Lack of refresher training, more training for referral and early identification. | | | | | | | | | | | | | | | | | | | | | | | | | | | | | | | | | | | | | | | | | | | | | | | | | | | | | | | | | | | | | | | | | | | | | | | | | | | | | | | | | | | | | | | | | 1. CBR workers should be trained on CBR Matrix activities  2. Home visits (community work) increases awareness towards disability and acceptance by community and families  3. CBR workers should have comprehensive training, including management skills  4. CBR workers should be training on advocacy and empowerment activities to increase awareness of persons with disabilities  5. Training should focus on referrals and identification of disability  6. Community ownership of programmes may increase acceptability and involvement | | | | | | | | | | | | | | | | | | | | | | | | | | | | | | | | | | | | | | | | | | | | | | | | | | | | | | | | | | | | | | | | | | | | | | | | | | | | | | | | | |  |
| **Title:** A mental health training program for community health workers in India: impact on knowledge and attitudes | | | | | | | | | | | | | | | | | | | | | | | | | | | | | | | | | | | | | | | | | | | | | | | | | | | | | | | | | | | | | | | | | | | | | | | | | | | | | | | | | | | | | | | | | | | | | | | | | | | | | | | | | | | | | | | | | | | | | | | | | | | | | | | | | | | | | | | | | | | | | | | | | | | | | | | | | | | | | | | | | | | | | | | | | | | | | | | | | | | | | | | | | | | | | | | | | | | | | | | | | | | | | | | | | | | | | | | | | | | | | | | | | | | | | | | | | | | | | | | | | | | | | | | | | | | | | | | | | | | | | | | | | | | | | | | |  |
| **Authors:** Armstrong, G., Kermode, M., Raja, S., Suja S., Chandra, P. & Jorm, A. | | | | | | | | | | | | | | | | | | | | | | | | | | | | | | | | | | | | | | | | | | | | | | | | | | | | | | | | | | | | | | | | | | | | | | | | | | | | | | | | | | | | | | | | | | | | | | | | | | | | | | | | | | | | | | | | | | | | | | | | | | | | | | | | | | | | | | | | | | | | | | | | | | | | | | | | | | | | | | | | | | | | | | | | | | | | | | | | | | | | | | | | | | | | | | | | | | | | | | | | | | | | | | | | | | | | **Year:** 2011 | | | | | | | | | | | | | | | | | | | | | | | | | | | | | | | | | | | | | | | | | | | | | | | | | | | | | | | | | | | |  |
| **Summary:** Lay health workers for mental health services including increasing recognition and providing support to communities | | | | | | | | | | | | | | | | | | | | | | | | | | | | | | | | | | | | | | | | | | | | | | | | | | | | | | | | | | | | | | | | | | | | | | | | | | | | | | | | | | | | | | | | | | | | | | | | | | | | | | | | | | | | | | | | | | | | | | | | | | | | | | | | | | | | | | | | | | | | | | | | | | | | | | | | | | | | | | | | | | | | | | | | | | | | | | | | | | | | | | | | | | | | | | | | | | | | | | | | | | | | | | | | | | | | | | | | | | | | | | | | | | | | | | | | | | | | | | | | | | | | | | | | | | | | | | | | | | | | | | | | | | | | | | | |  |
| **Setting** | | | | | | | | | | | | | | | **Design/**  **Methods** | | | | | | | | | | | | | | | | | | **Population** | | | | | | | | | | | | | | | | | | | | | | | | | | | | | **Intervention** | | | | | | | | | | | | | | | | | | | | | | | | | | | | | | | | | | | | | | | | | | | | | | | | | | | | | | | | | | | | | | | **Workforce Characteristics** | | | | | | | | | | | | | | | | | | | | | | | | | | | | | | | | | | | | | | | | | | | | | | | | | | | | | | | | | | | | | | | | | | | | | | | | | | | | | | | | | | | | | | | | | | | | | | | | | | | | | | | | | | | | | | | | | | | | | | | | | | | | | | | | | | | | | | | | | | | | | | | | | | |  |
|  |  |  |  |  |  |  |  |  |  |  |  |  |  |  |  |  |  |  |  |  |  |  |  |  |  |  |  |  |  |  |  |  |  |  |  |  |  |  |  |  |  |  |  |  |  |  |  |  |  |  |  |  |  |  |  |  |  |  |  |  |  |  |  |  |  |  |  |  |  |  |  |  |  |  |  |  |  |  |  |  |  |  |  |  |  |  |  |  |  |  |  |  |  |  |  |  |  |  |  |  |  |  |  |  |  |  |  |  |  |  |  |  |  |  |  |  |  |  |  |  |  |  |  |  | **Cadres** | | | | | | | | | | | | | | | | | | | | | | | | | | | | | | | | | | | | | | | | **Description** | | | | | | | | | | | | | | | | | | | | | | | | | | | | **Training** | | | | | | | | | | | | | | | | | | | | | | | | | | | | | | | | | | | | | | | | | | | | | | | | | | | | | **Supervision** | | | | | | | | | | | | | | | | | | | | | | | | **Misc.** | |  |
| Doddaballapur Taluk, Bangalore Rural District, Karnataka, India. | | | | | | | | | | | | | | | Evaluation using pre-test post-test design. | | | | | | | | | | | | | | | | | | Cadre of Community Health Workers (n=70) | | | | | | | | | | | | | | | | | | | | | | | | | | | | | An “introduction to mental health for uninitiated community health workers”. Training course aiming to increase recognition of mental illness, enhance appropriate response and referral, support individual with mental disorders and their families, improve mental health promotion in their area. | | | | | | | | | | | | | | | | | | | | | | | | | | | | | | | | | | | | | | | | | | | | | | | | | | | | | | | | | | | | | | | Community Health Workers (CHWs), including: Junior Health Assistants, Village Rehabilitation Workers, and ASHA workers. | | | | | | | | | | | | | | | | | | | | | | | | | | | | | | | | | | | | | | | | CHWs sourced through Gramina Abrudaya Seva Samstha (GASS), an NGO in the area. All government funded cadres | | | | | | | | | | | | | | | | | | | | | | | | | | | | In three separate groups of 23-24 participants, with 2 facilitators. Local facilitators only had ‘moderate’ understanding of community mental health to reflect real world scenario. 4 day course | | | | | | | | | | | | | | | | | | | | | | | | | | | | | | | | | | | | | | | | | | | | | | | | | | | | |  | | | | | | | | | | | | | | | | | | | | | | | | **Quality: 3**  MMAT- Quant4 | |  |
| **CMOCs** | | | | | | | | | | | | | | | | | | | | | | | | | | | | | | | | | | | | | | | | | | | | | | | | | | | | | | | | | | | | | | | | | | | | | | | | | | | | | | | | | | | | | | | | | | | | | | | | | | | | | | | | | | | | | | | | | | | | | | | | | | | | | | | | | | | | | | | | | | | | | | | | | | | | | | | | | | | | | | | | | | | | | | | | | | | | | | | | | | | | | | | | | | | | | | | | | | | | | | | | | | | | | | | | | | | | | | | | | | | | | | | | | | | | | | | | | | | | | | | | | | | | | | | | | | | | | | | | | | | | | | | | | | | | | | | |  |
| **Context** | | | | | | | | | | | | | | | | | | | | | | | **Mechanisms** | | | | | | | | | | | | | | | | | | | | | | | | | | | | | | | | | | | | | | | | | | | | | | | | | | | | | | | | | | | | | | | | | | | | **Outcomes** | | | | | | | | | | | | | | | | | | | | | | | | | | | | | | | | | | | | | | | | | | | | | | | | | | | | | | | | | | | | | | | | | | | | | | | | | | | | | | | | | | | | | | | | | | | | | | | | | | | | | | | | | | | | | | | | | | | | | | | | | | | | **CMOCs** | | | | | | | | | | | | | | | | | | | | | | | | | | | | | | | | | | | | | | | | | | | | | | | | | | | | | | | | |  |
| Community health workers identified from an NGO operating in the area, all government funded, and working Junior Health Assistants, Village Rehabilitation Workers or ASHA workers.  Low levels of mental health literacy at baseline (9.1% could identify psychosis, 22% depression). High levels of individuals advocating for inappropriate treatment for mental illness. Also assessed perceived outcomes and attitudes towards people with mental disorders. | | | | | | | | | | | | | | | | | | | | | | | 1.Poor perceptions of PWD even after training. Could be due to training did not integrate people with mental illness.  2.Trainers only had moderate experience, so though it reflects the most likely scenario in this situations, could be detrimental to have trainers who are not specifically trained.  3. Training that does not incorporate persons with disabilities may not be as effective in increasing awareness or reducing stigma.  4.With little training (4 days) already working health workers can improve healthy behaviours towards individuals with disability, specifically in areas of pharmacological interventions and ability to recognize depression and psychosis.  5.Training courses that that don’t have focus on social aspects of mental illness and disability do little to change a health worker’s perception of these individuals. | | | | | | | | | | | | | | | | | | | | | | | | | | | | | | | | | | | | | | | | | | | | | | | | | | | | | | | | | | | | | | | | | | | | Participant’s ability to recognize mental disorders (depression and psychosis) was improved, with a significantly significant increase in identification after training, with a drop after 3 months. However, still remained significant (22.7% to 50.0% to 43.9% for depression) and 9.1% to 27.3% to 34.8% for psychosis.  Sustained decrease in the percentage of participants endorsing potentially useless pharmacological interventions for both depression and psychosis.  No clear difference in the use of non-pharmacological interventions.  Significant difference in participants endorsing hospital admission for a person with depression and reduction in participants endorsing marriage as a helpful intervention for psychosis.  The training had little effect on participant’s perceived outcomes for persons with mental illnesses, except that are significant reduction was seen in the percentage of participants identifying full recover or no further problems for individuals with psychosis if appropriate help was received.  There was little change in participant’s attitude towards individuals with mental illness after training. Changes were seen however in: it is best to avoid people (with depression) from 21.2% to 6.1% to 4.5%, and that depression is a sign of weakness (84.8% to 89.4% to 62.1%). However, no changes were seen in: people with this problem can snap out of it (64.6% to 53.0%); This problem is not a real medical illness (54.5% to 59.1%); people with this problem are dangerous (30.8% to 33.3%); people with this problem are erratic (77.3% to 80.3%); I would not vote for a person with this problem (50.0% to 53.0%).  For psychosis there were no sustained changes that could be attributed to the training, that were sustained. In fact, some were even increased (though not significantly). | | | | | | | | | | | | | | | | | | | | | | | | | | | | | | | | | | | | | | | | | | | | | | | | | | | | | | | | | | | | | | | | | | | | | | | | | | | | | | | | | | | | | | | | | | | | | | | | | | | | | | | | | | | | | | | | | | | | | | | | | | | | 1.Community-based workers need to acquire relevant knowledge and skills to recognize, refer and support individuals experiencing mental disorders.  2.Task-shifting, having already working CHWs and training them specifically, may help to meet the need to increase human resources for mental health.  4. Persons with disabilities should be included in the training sessions.  5. Training for health works should include social aspects of disability, especially mental illness.  6. The perceptions that health workers have on persons with disabilities, especially mental health, should be considered and assessed prior to intervention with the appropriate training to follow findings.  7.Trainers with only moderate experience in mental health should undergo further training as well as training of trainers before training others.  8. Training alone is not enough to be effective, proper training that meets the needs of populations as well as the workers themselves, with adequate trainers. | | | | | | | | | | | | | | | | | | | | | | | | | | | | | | | | | | | | | | | | | | | | | | | | | | | | | | | | |  |
| **Title:** Integrating Mental Health and Development: A Study of the BasicNeeds Model in Nepal | | | | | | | | | | | | | | | | | | | | | | | | | | | | | | | | | | | | | | | | | | | | | | | | | | | | | | | | | | | | | | | | | | | | | | | | | | | | | | | | | | | | | | | | | | | | | | | | | | | | | | | | | | | | | | | | | | | | | | | | | | | | | | | | | | | | | | | | | | | | | | | | | | | | | | | | | | | | | | | | | | | | | | | | | | | | | | | | | | | | | | | | | | | | | | | | | | | | | | | | | | | | | | | | | | | | | | | | | | | | | | | | | | | | | | | | | | | | | | | | | | | | | | | | | | | | | | | | | | | | | | | | | | | | | | | |  |
| **Authors:** Raja, S., Underhill, C., Shrestha, P., Sunder, U., Mannarath, S.,Wood, SK. & Patel, V. | | | | | | | | | | | | | | | | | | | | | | | | | | | | | | | | | | | | | | | | | | | | | | | | | | | | | | | | | | | | | | | | | | | | | | | | | | | | | | | | | | | | | | | | | | | | | | | | | | | | | | | | | | | | | | | | | | | | | | | | | | | | | | | | | | | | | | | | | | | | | | | | | | | | | | | | | | | | | | | | | | | | | | | | | | | | | | | | | | | | | | | | | | | | | | | | | | | | | | | | | | | | | | | | | **Year:** 2012 | | | | | | | | | | | | | | | | | | | | | | | | | | | | | | | | | | | | | | | | | | | | | | | | | | | | | | | | | | | | | | |  |
| **Summary:** This paper reports on a Case Study from an NGO (Basic Needs) working with a community-based integrated Mental Health and Development (MHD) programme in Nepal. | | | | | | | | | | | | | | | | | | | | | | | | | | | | | | | | | | | | | | | | | | | | | | | | | | | | | | | | | | | | | | | | | | | | | | | | | | | | | | | | | | | | | | | | | | | | | | | | | | | | | | | | | | | | | | | | | | | | | | | | | | | | | | | | | | | | | | | | | | | | | | | | | | | | | | | | | | | | | | | | | | | | | | | | | | | | | | | | | | | | | | | | | | | | | | | | | | | | | | | | | | | | | | | | | | | | | | | | | | | | | | | | | | | | | | | | | | | | | | | | | | | | | | | | | | | | | | | | | | | | | | | | | | | | | | | |  |
| **Setting** | | | **Design/Methods** | | | | | | | | | | | | | | **Population** | | | | | | | | | | | | | | | | | | | | | | | | | | | **Intervention** | | | | | | | | | | | | | | | | | | | | | | | | | | | | | | | | | | | | | | | | | | | | | | | | | | | | | | | | | | | | | | | | | | | | | | | | | | | | | | | | | | | | | | | | | | | | | | | | | | | | | | | | | | | | | | | | | | | | | **Workforce Characteristics** | | | | | | | | | | | | | | | | | | | | | | | | | | | | | | | | | | | | | | | | | | | | | | | | | | | | | | | | | | | | | | | | | | | | | | | | | | | | | | | | | | | | | | | | | | | | | | | | | | | | | | | | | | | | | | |  |
|  |  |  |  |  |  |  |  |  |  |  |  |  |  |  |  |  |  |  |  |  |  |  |  |  |  |  |  |  |  |  |  |  |  |  |  |  |  |  |  |  |  |  |  |  |  |  |  |  |  |  |  |  |  |  |  |  |  |  |  |  |  |  |  |  |  |  |  |  |  |  |  |  |  |  |  |  |  |  |  |  |  |  |  |  |  |  |  |  |  |  |  |  |  |  |  |  |  |  |  |  |  |  |  |  |  |  |  |  |  |  |  |  |  |  |  |  |  |  |  |  |  |  |  |  |  |  |  |  |  |  |  |  |  |  |  |  |  |  |  |  |  |  |  |  |  |  |  |  |  |  |  |  |  |  |  |  |  |  |  |  | **Cadres** | | | | | | | | | | | | | | | | | | | | | | | | | | | | | | **Description** | | | | | | | | | | | | | | | | | | | | | | | | | | | | | | | | | | | | **Training** | | | | | | | | | | | | | | | | | | **Supervision** | | | | | | | | | | | | | | | | | | | | | | | | | **Misc.** | |  |
| Baglung and Myagdi Districts, Nepal. | | | Case Study. | | | | | | | | | | | | | | Individuals affected by mental health issues. | | | | | | | | | | | | | | | | | | | | | | | | | | | Programme: Treatment at mental health clinics (regional hospital), where government supplies space and staff. Mental health focal person (senior health assistant) coordinates the services, psychiatrist who diagnoses and prescribes medication, VDCs provide FCHVs to refer and assist in follow-up clinics.  Follow-up clinics – have MH focal person and other trained staff to conduct telephone consultations with psychiatrist at regional hospital.  Home visits – for monitoring and support, with FCHVs monitor medicines, support families and assist in livelihood activities. NGO community based workers compliment FCHVs. | | | | | | | | | | | | | | | | | | | | | | | | | | | | | | | | | | | | | | | | | | | | | | | | | | | | | | | | | | | | | | | | | | | | | | | | | | | | | | | | | | | | | | | | | | | | | | | | | | | | | | | | | | | | | | | | | | | | | VDCs (Village Development Committee) and Female Community Health Volunteers (FCHVs), Community Based Workers (CBWs), Auxiliary Health Workers, Health Assistant, and clinical staff. | | | | | | | | | | | | | | | | | | | | | | | | | | | | | | Identification of PWD in villages, after coaching and supervision run follow-up clinics in villages. | | | | | | | | | | | | | | | | | | | | | | | | | | | | | | | | | | | | Not discussed, though situational assessment conducted prior to programme | | | | | | | | | | | | | | | | | | Due to demand and services being brought to local clinics, CBW given cell phones to connect with chief psychiatrist | | | | | | | | | | | | | | | | | | | | | | | | | **Quality: NA** Case study | |  |
| **CMOCs** | | | | | | | | | | | | | | | | | | | | | | | | | | | | | | | | | | | | | | | | | | | | | | | | | | | | | | | | | | | | | | | | | | | | | | | | | | | | | | | | | | | | | | | | | | | | | | | | | | | | | | | | | | | | | | | | | | | | | | | | | | | | | | | | | | | | | | | | | | | | | | | | | | | | | | | | | | | | | | | | | | | | | | | | | | | | | | | | | | | | | | | | | | | | | | | | | | | | | | | | | | | | | | | | | | | | | | | | | | | | | | | | | | | | | | | | | | | | | | | | | | | | | | | | | | | | | | | | | | | | | | | | | | | | | | | |  |
| **Context** | | | | | | | | | | | | | | | | | | | | | | | | | | | | | | | | | | | | | | | | | | | | | | | | | | | | | | | | | | | | | | | | | | | | | | | | | | | | | | | | | | | | | | | | | | | | | | | | | | | | **Mechanisms** | | | | | | | | | | | | | | | | | | | | | | | | | | | | | | | | | | | | | | | | | | | | | | | | | | | | | | | | | | | | | | | | | | | | **Outcomes** | | | | | | | | | | | | | | | | | | | | | | | | | | | | | | | | | | | | | | | | | | | | | | | | | | | | **CMOCs** | | | | | | | | | | | | | | | | | | | | | | | | | | | | | | | | | | | | | | | | | | | | | | | | | | | |  |
| Approx. 0.14% of health expenditure spent on mental health, and Nepal has no mental health legislation. Only one public hospital offering psychiatric services and only 32 psychiatrics for a population of over 28 million.  Nepal programme works through local NGO with an expertise in CBR and Livelihoods, titled Livelihoods Education and Development Society (LEADS). The 4-year programme in Nepal began in 2010 and operates in two districts, Baglung and Myagdi – where situational assessment found no mental health human resources trained.  MHD programme works within 5 modules for people with mental illness and epilepsy: management, research, community mental health, livelihoods and capacity building.  Works in a referral, and multi-tiered system with specialist services at mental health camps, and community based workers and community committees in the village. List of human resources as follows:  Mental health camp: team of health professions to carry out in-patient services in a community setting at a specified interval; village health workers supported by village committees, find and refer individuals to mental health camps.  They identify and mobilize individuals via home visits for field consultations.  Community mobilization through distribution of materials in village development committee areas, sing NGO, community and government staff.  Training consisting of workshops for physicians, health staff, female community health volunteers and community based workers at district hospital and health centres. NGO funds and organized training, with government providing psychiatrist who runs training. | | | | | | | | | | | | | | | | | | | | | | | | | | | | | | | | | | | | | | | | | | | | | | | | | | | | | | | | | | | | | | | | | | | | | | | | | | | | | | | | | | | | | | | | | | | | | | | | | | | | 1.Increasing awareness of community, acceptability of community, and knowledge of health workers.  2.Utilizing a multi-tiered treatment system, including facility based and community, involving government and NGO, can have positive impacts on individual’s health.  3.Bringing services to the community by increasing awareness on mental health and providing community workers may increase number of referrals due to decreased stigma and increased awareness.  4.Community based services can more efficiently identify individuals with mental illness and then refer.  5.Increasing training and skills of health workforce to respond to PWD/MI need to be done in consideration with other aspects of the health service. | | | | | | | | | | | | | | | | | | | | | | | | | | | | | | | | | | | | | | | | | | | | | | | | | | | | | | | | | | | | | | | | | | | | 311 patients registered between May 2010 and March 2011.  269/311 patients reported to show improvements.  Increasing number of referrals from home visits and also self-referral.  Reduction in out-of-pocket expenditure on medications and services.  Increased number of individuals generating income based on livelihoods programme.  Still struggle with the capacity of the health centre to respond to the demand for services. With key challenges being availability of qualified staff, and medicines.  SIM cards provided to CBR workers to maintain contact for supervision and referrals with chief psychiatrist. | | | | | | | | | | | | | | | | | | | | | | | | | | | | | | | | | | | | | | | | | | | | | | | | | | | | 1.Focal person required, especially in a tiered-delivery system  2. Situational assessment of HR, demands, training etc. prior to intervention development for increased efficiency  3.Multi-facated (CBR Matrix) programme at the community level  4. Tiered system including treatment services, follow-up and home based care.  5. Collaboration between NGOs, community, and MoH important for bringing services to communities.  6. Ability to system to respond to challenges creatively (SIM cards for supervision).  7. Skills in empowerment and community development for community based health workers  8. HR workers to work towards strengthening health systems and influencing policy  9. Planning for increasing demand in services with appropriate supply of HR.  10. CBW for referrals and follow-up home visits for PWD. | | | | | | | | | | | | | | | | | | | | | | | | | | | | | | | | | | | | | | | | | | | | | | | | | | | |  |
| **Title:** Evaluating the impact of a community-based rehabilitation intervention | | | | | | | | | | | | | | | | | | | | | | | | | | | | | | | | | | | | | | | | | | | | | | | | | | | | | | | | | | | | | | | | | | | | | | | | | | | | | | | | | | | | | | | | | | | | | | | | | | | | | | | | | | | | | | | | | | | | | | | | | | | | | | | | | | | | | | | | | | | | | | | | | | | | | | | | | | | | | | | | | | | | | | | | | | | | | | | | | | | | | | | | | | | | | | | | | | | | | | | | | | | | | | | | | | | | | | | | | | | | | | | | | | | | | | | | | | | | | | | | | | | | | | | | | | | | | | | | | | | | | | | | | | | | | | | |  |
| **Authors:** Hartley, S. | | | | | | | | | | | | | | | | | | | | | | | | | | | | | | | | | | | | | | | | | | | | | | | | | | | | | | | | | | | | | | | | | | | | | | | | | | | | | | | | | | | | | | | | | | | | | | | | | | | | | | | | | | | | | | | | | | | | | | | | | | | | | | | | | | | | | | | | | | | | | | | | | | | | | | | | | | | | | | | | | | | | | | | | | | | | | | | | | | | | | | | | | | | | | | | | | | | | | | | | | | | | | | | | | | | | **Year:** 2001-2003 | | | | | | | | | | | | | | | | | | | | | | | | | | | | | | | | | | | | | | | | | | | | | | | | | | | | | | | | | | | |  |
| **Summary**: Brief report on project designed to increase knowledge on interventions for children with communication problems in Kenya, by evaluating the impact of using local women’s groups for community-based action. | | | | | | | | | | | | | | | | | | | | | | | | | | | | | | | | | | | | | | | | | | | | | | | | | | | | | | | | | | | | | | | | | | | | | | | | | | | | | | | | | | | | | | | | | | | | | | | | | | | | | | | | | | | | | | | | | | | | | | | | | | | | | | | | | | | | | | | | | | | | | | | | | | | | | | | | | | | | | | | | | | | | | | | | | | | | | | | | | | | | | | | | | | | | | | | | | | | | | | | | | | | | | | | | | | | | | | | | | | | | | | | | | | | | | | | | | | | | | | | | | | | | | | | | | | | | | | | | | | | | | | | | | | | | | | | |  |
| **Setting** | | | | | | | **Design/**  **Methods** | | | | | | | | | | | | | | | | | | | | | | **Population** | | | | | | | | | | | | | | | | | | | | | | | | | | | | | | | | | | | | | **Intervention** | | | | | | | | | | | | | | | | | | | | | | | | | | | | | | | | | | | | | | | | | | | | | | | | | **Workforce Characteristics** | | | | | | | | | | | | | | | | | | | | | | | | | | | | | | | | | | | | | | | | | | | | | | | | | | | | | | | | | | | | | | | | | | | | | | | | | | | | | | | | | | | | | | | | | | | | | | | | | | | | | | | | | | | | | | | | | | | | | | | | | | | | | | | | | | | | | | | | | | | | | | | | | | | | | | | | | | | | |  |
|  |  |  |  |  |  |  |  |  |  |  |  |  |  |  |  |  |  |  |  |  |  |  |  |  |  |  |  |  |  |  |  |  |  |  |  |  |  |  |  |  |  |  |  |  |  |  |  |  |  |  |  |  |  |  |  |  |  |  |  |  |  |  |  |  |  |  |  |  |  |  |  |  |  |  |  |  |  |  |  |  |  |  |  |  |  |  |  |  |  |  |  |  |  |  |  |  |  |  |  |  |  |  |  |  |  |  |  |  |  |  |  |  |  |  | **Cadres** | | | | | | | | | | | | | | | | | | | | | | | | | | | | | | | | | | | | | | | **Description** | | | | | | | | | | | | | | | | | | | **Training** | | | | | | | | | | | | | | | | | | | | | | | | | | | | | | | | | | | | | | | | | | | | | | | | | | | | | | | | | **Supervision** | | | | | | | | | | | | | | | | | | | | | | | **Misc.** | | | | | | | | | | | | | | | | | | |  |
| Kilifi District, Kenya | | | | | | | Report, prospective. | | | | | | | | | | | | | | | | | | | | | | 334 Children with communication difficulties and their mothers. | | | | | | | | | | | | | | | | | | | | | | | | | | | | | | | | | | | | | Randomly selected 8 already existing women’s groups in the area. Series of workshops for each group to work out individual intervention plans for children (20 per group approx.), for community based action. Women’s groups develop individual intervention plans for children. | | | | | | | | | | | | | | | | | | | | | | | | | | | | | | | | | | | | | | | | | | | | | | | | | Local women’s groups | | | | | | | | | | | | | | | | | | | | | | | | | | | | | | | | | | | | | | | Local women’s groups already established in the area | | | | | | | | | | | | | | | | | | | Series of workshops for women’s groups to assist in development of individual intervention plans for children. | | | | | | | | | | | | | | | | | | | | | | | | | | | | | | | | | | | | | | | | | | | | | | | | | | | | | | | | | Not discussed. | | | | | | | | | | | | | | | | | | | | | | | Approximately 20 children per group.  **Quality – NA** Descriptive report | | | | | | | | | | | | | | | | | | |  |
| **CMOCs** | | | | | | | | | | | | | | | | | | | | | | | | | | | | | | | | | | | | | | | | | | | | | | | | | | | | | | | | | | | | | | | | | | | | | | | | | | | | | | | | | | | | | | | | | | | | | | | | | | | | | | | | | | | | | | | | | | | | | | | | | | | | | | | | | | | | | | | | | | | | | | | | | | | | | | | | | | | | | | | | | | | | | | | | | | | | | | | | | | | | | | | | | | | | | | | | | | | | | | | | | | | | | | | | | | | | | | | | | | | | | | | | | | | | | | | | | | | | | | | | | | | | | | | | | | | | | | | | | | | | | | | | | | | | | | | |  |
| **Context** | | | | | | | | | | | | | | | | | | | | | | | | | | | | | | | | | | | | | | | | | | | | | | | | | | | | | | | | | | | | | | | | | | | | | | **Mechanisms** | | | | | | | | | | | | | | | | | | | | | | | | | | | | | | | | | | | | | | | | | | | | | | | | | | | | | | | | | | | | | | | | | | | | | | | | | | | | | | **Outcomes** | | | | | | | | | | | | | | | | | | | | | | | | | | | | | | | | | | | | | | | | | | | | | | | | | | **CMOCs** | | | | | | | | | | | | | | | | | | | | | | | | | | | | | | | | | | | | | | | | | | | | | | | | | | | | | | | | | | | | | | | | | | | | | | | | | |  |
| Half of all disabled children in LICs have communication difficulties.  Approximately 20 million children worldwide and half a million children in Kenya alone have communication difficulties.  Kilifi region, in Kenya, estimated to have 40,000 of 800,000 children affected by communication issues.  4 study phases – developing and modifying tools and measurements for intervention; identification of 334 children with communication problems; assignment of children to two groups, intervention and control.  Develop an outcome measuring tool (COMT) for “Communication Outcome Measuring Tool.  Develop a QoL tool (questionnaire) to measuring children’s level of happiness, needs, and sense of security and self-confidence and access to information. | | | | | | | | | | | | | | | | | | | | | | | | | | | | | | | | | | | | | | | | | | | | | | | | | | | | | | | | | | | | | | | | | | | | | | 1.Dividing children depending on disability might not be an effective tool or culturally acceptable in community work. Speculating that a wider ambit and inclusiveness may be more culturally appropriate and acceptable.  2.Already existing social groups (i.e. women’s groups) can be effective in providing support to PWD and their families, and lead to increased community awareness and advocacy.  3. Working groups can assist in the development of intervention plans for children with communication difficulties | | | | | | | | | | | | | | | | | | | | | | | | | | | | | | | | | | | | | | | | | | | | | | | | | | | | | | | | | | | | | | | | | | | | | | | | | | | | | | Women’s groups were enthusiastic about the project, indicating to the authors that they may be effective CBR workers to recruit.  Women’s group enthusiastic about incorporating mothers of disabled children and representing other disabled people in their groups. Some have started their own activities for persons with disabilities outside the project.  Women’s groups have difficult discerning between disabilities in children and include all children with disabilities. | | | | | | | | | | | | | | | | | | | | | | | | | | | | | | | | | | | | | | | | | | | | | | | | | | 1.Rehabilitation should be inclusive and not segregate by type of disability or rehabilitation service needed.  2.Rehabilitation workers should be knowledgeable of all types of disability and have appropriate training to be able to discern between types of disability.  3. Community groups should be trained in identification and rehabilitation of persons with disabilities  4.Training of lay community rehabilitation workers needs to focus on appropriate identification of persons with disability.  5. Community groups/lay workers accepted by parents for rehabilitation for children interventions. | | | | | | | | | | | | | | | | | | | | | | | | | | | | | | | | | | | | | | | | | | | | | | | | | | | | | | | | | | | | | | | | | | | | | | | | | |  |
| **Title:** Evaluation of the CBR programme in Palestine – from the perspective of persons with disabilities themselves | | | | | | | | | | | | | | | | | | | | | | | | | | | | | | | | | | | | | | | | | | | | | | | | | | | | | | | | | | | | | | | | | | | | | | | | | | | | | | | | | | | | | | | | | | | | | | | | | | | | | | | | | | | | | | | | | | | | | | | | | | | | | | | | | | | | | | | | | | | | | | | | | | | | | | | | | | | | | | | | | | | | | | | | | | | | | | | | | | | | | | | | | | | | | | | | | | | | | | | | | | | | | | | | | | | | | | | | | | | | | | | | | | | | | | | | | | | | | | | | | | | | | | | | | | | | | | | | | | | | | | | | | | | | | | | |  |
| **Authors:** Nilsson, A. & Qutteina, M. | | | | | | | | | | | | | | | | | | | | | | | | | | | | | | | | | | | | | | | | | | | | | | | | | | | | | | | | | | | | | | | | | | | | | | | | | | | | | | | | | | | | | | | | | | | | | | | | | | | | | | | | | | | | | | | | | | | | | | | | | | | | | | | | | | | | | | | | | | | | | | | | | | | | | | | | | | | | | | | | | | | | | | | | | | | | | | | | | | | | | | | | | | | | | | | | | | | | | | | | | | | | | | | | | | | | **Year:** 2005 | | | | | | | | | | | | | | | | | | | | | | | | | | | | | | | | | | | | | | | | | | | | | | | | | | | | | | | | | | | |  |
| **Summary:** Evaluation of CBR programme in Palestine using lay health workers in a tier delivery model, in order to provide recommendations for more effective programming | | | | | | | | | | | | | | | | | | | | | | | | | | | | | | | | | | | | | | | | | | | | | | | | | | | | | | | | | | | | | | | | | | | | | | | | | | | | | | | | | | | | | | | | | | | | | | | | | | | | | | | | | | | | | | | | | | | | | | | | | | | | | | | | | | | | | | | | | | | | | | | | | | | | | | | | | | | | | | | | | | | | | | | | | | | | | | | | | | | | | | | | | | | | | | | | | | | | | | | | | | | | | | | | | | | | | | | | | | | | | | | | | | | | | | | | | | | | | | | | | | | | | | | | | | | | | | | | | | | | | | | | | | | | | | | |  |
| **Setting** | | | | | | | | | | | **Design/Methods** | | | | | | | | | | | | | | | | | | | | | | | | | **Population** | | | | | | | | | | | | | | | | | | | | | | | | | | | | | | | | | | | | | | | | | | | | | | | | | | **Intervention** | | | | | | | | | | | | | | | | | | | | | | | | | | | | | | | | | | | | | | | | | | | | | | **Workforce Characteristics** | | | | | | | | | | | | | | | | | | | | | | | | | | | | | | | | | | | | | | | | | | | | | | | | | | | | | | | | | | | | | | | | | | | | | | | | | | | | | | | | | | | | | | | | | | | | | | | | | | | | | | | | | | | | | | | | | | | | | | | | | | | | | | | | | | | | | | | | | | | |  |
|  |  |  |  |  |  |  |  |  |  |  |  | | | | | | | | | | | | | | | | | | | | | | | | |  | | | | | | | | | | | | | | | | | | | | | | | | | | | | | | | | | | | | | | | | | | | | | | | | | |  | | | | | | | | | | | | | | | | | | | | | | | | | | | | | | | | | | | | | | | | | | | | | **Cadres** | | | | | | | | | | | | | | | | | | | | | | | | | | | | **Description** | | | | | | | | | | | | | | | | | | | | | | | | | | | | | | | | | | **Training** | | | | | | | | | | | | | | | | | | | | | | | | | | | | | | | | | | | | | | | | | **Supervision** | | | | | | | | | | | | | | | | | | | | | | | | | | **Misc.** | | | | | | | | | | | | |
| Palestine, 5 regions: Jenin, Nablus, Sough, Central and Gaza. | | | | | | | | | | External evaluation report using qualitative methods of interviews, focus groups and observations. | | | | | | | | | | | | | | | | | | | | | | | | | 25 PWDs, 90 PWDs of 5-7 focus groups, DPOs and other CBR staff of all five regions, finally by observations during visits to homes communities and schools | | | | | | | | | | | | | | | | | | | | | | | | | | | | | | | | | | | | | | | | | | | | | | | | | | Tiered based CBR programme using outreach services with CBR workers, immediate services including therapies, and specialized services, with CBR implemented by NGOs | | | | | | | | | | | | | | | | | | | | | | | | | | | | | | | | | | | | | | | | | | | | | Community based rehabilitation (CBR) workers. | | | | | | | | | | | | | | | | | | | | | | | | | | | | Role and training focusing on social counseling, skills training and referrals. Work at the primary level, conducting outreach in coordination with NGOs. Provide support in the home. | | | | | | | | | | | | | | | | | | | | | | | | | | | | | | | | | | Focused on social counseling, skills training for PWD and referrals to more specialized services. | | | | | | | | | | | | | | | | | | | | | | | | | | | | | | | | | | | | | | | | | Not mentioned specifically, however CBR workers work with, or are employed by NGOs in their region. | | | | | | | | | | | | | | | | | | | | | | | | | | CBR workers have 50-70 active cases on average.  **Quality: 2.5**  MMAT - Qualitative | | | | | | | | | | | | |  |
| **CMOCs** | | | | | | | | | | | | | | | | | | | | | | | | | | | | | | | | | | | | | | | | | | | | | | | | | | | | | | | | | | | | | | | | | | | | | | | | | | | | | | | | | | | | | | | | | | | | | | | | | | | | | | | | | | | | | | | | | | | | | | | | | | | | | | | | | | | | | | | | | | | | | | | | | | | | | | | | | | | | | | | | | | | | | | | | | | | | | | | | | | | | | | | | | | | | | | | | | | | | | | | | | | | | | | | | | | | | | | | | | | | | | | | | | | | | | | | | | | | | | | | | | | | | | | | | | | | | | | | | | | | | | | | | | | | | | | | |  |
| **Context** | | | | | | | | | | | | | | | | | | | | | | | | | | | | | | | | | | | | | | | | | | | | | | | | | | | | | | | | | | | | | | | | | | | | | | | | | | | | | | | | | | | | **Mechanisms** | | | | | | | | | | | | | | | | | | | | | | | | | | | | | | | | | | | | | | | | | | | | | | | | | | | | | | | | | | | | | | | | | | | **Outcomes** | | | | | | | | | | | | | | | | | | | | | | | | | | | | | | | | | | | | | | | | | | | | | | | | | | | | | | | | | | | | | | | | | | | | | | | | | | | | | | | | | | | | | **CMOCs** | | | | | | | | | | | | | | | | | | | | | | | | | | | | | | | | | | | |  |
| CBRP programme has been running since 1990 in Palestine, with NAD assistance, through technical support to 17 partnering NGOs. Active in more than 200 areas, and covers 60% of West Bank and Gaza, reaching over 35,000 persons with disabilities. Network of NGOs have organized themselves into 5 regional committees, responsible for planning and implementing the programme.  Gaza and central region of Palestine more urban and have access to more specialized medical care. More implementing NGO partners working on programme.  Other regions of Palestine more rural and have less access to formal care, also work with fewer NGOs implementing programme. Services for PWD organized at 3 levels: primary, secondary, and tertiary. Primary is outreach and CBR by NGOs. CBR workers have 50-70 active cases. CBR workers engage individuals in their house to provide support, and work with communities.Secondary level offers immediate services including therapies and assistive devices, which are organized by both governmental and NGOs, though the minority (13/114) are under the supervision of the government. Most of these are concentrated in the urban cities.Tertiary – medical centers that provide specialized services for PWD, and there are only 4 mostly located in urban areas.Most individuals cannot access secondary or tertiary centres.CBR workers main source of care for individuals, though role and training focusing on social counseling, skills training and referrals. | | | | | | | | | | | | | | | | | | | | | | | | | | | | | | | | | | | | | | | | | | | | | | | | | | | | | | | | | | | | | | | | | | | | | | | | | | | | | | | | | | | | 1.In contexts with use of devices CBR workers needs more training on devices and physical rehabilitation, or how to properly manage and implement or where to refer.  2.CBR workers impact on PWD self-esteem and relationships, especially in areas with more specialized services are lacking.  3.Having PWD as the community health workforce can increase respect for workers.  4.Rural based areas with limited access to specialized services rely on CBR work.  5. Even if not trained specifically for, CBR workers often act as counselors or peer-supporters for PWD.  6.CBR workers often assist PWD in social aspects of disability and rehabilitation.  7. In tier systems of delivery, important for all levels to be strengthened and reflect demand for service.  8. More rural areas with lack of access to specialized services heavily rely on CBR services and might lack health rehabilitation. | | | | | | | | | | | | | | | | | | | | | | | | | | | | | | | | | | | | | | | | | | | | | | | | | | | | | | | | | | | | | | | | | | | CBR programme found to have a large impact of individual self-esteem and emotional well-being, especially: interpersonal relations; social inclusion; personal development. With users often attributing CBR workers. Moral support identified by all (except Gaza participants) as the most useful intervention.  Limited impact on physical well being, especially in areas other than Gaza and Central region where there is access to more secondary and tertiary care. Level of satisfaction dependent on access to services.  Limited impact on individual’s rights, self-determination, and influence in community. No self-help groups organized, very little political agenda. Though parents expressed wanting to deal with issues within families. Respect and strong influence when PWD were CBR workers, however rare for PWD to become CBR workers or decision makers.  CBR workers often assisted with areas other than disability. Home visits effective way to build relationships, and people requested more of them, and more CBR workers. Referral system in Gaza and central region of CBR to secondary considered important. CBR workers in region also more specialized (nurses) and more men.  Some individuals with disability (especially severe and deaf persons) were either left out or not receiving appropriate care, as workers unsure of services to provide. Some CBR workers identified that it is emotionally difficult with some cases. | | | | | | | | | | | | | | | | | | | | | | | | | | | | | | | | | | | | | | | | | | | | | | | | | | | | | | | | | | | | | | | | | | | | | | | | | | | | | | | | | | | | | 1.PWD should be given priority to be trained as CBR workers.  2.All CBR workers should have training on basic psychosocial support, counseling skill and peer-support skills.  3.CBR workers should have their own counseling sessions and systems in place for seeking support, to allow for coping and debriefing of difficulties of job.  4.Access to and HR allocation, especially in rural areas, can limit the health related rehabilitation of PWD.  5. CBR workers visiting individuals at home effective and accepted means of reaching PWD.  6. CBR workers requiring training on assistive devices  7. CBR workers in tier system, especially in rural areas, require basic rehabilitation skills that are not limited to social, referrals and skills training. | | | | | | | | | | | | | | | | | | | | | | | | | | | | | | | | | | | |  |
| **Title:** Evaluation of Support to CBR Programme in Lesotho | | | | | | | | | | | | | | | | | | | | | | | | | | | | | | | | | | | | | | | | | | | | | | | | | | | | | | | | | | | | | | | | | | | | | | | | | | | | | | | | | | | | | | | | | | | | | | | | | | | | | | | | | | | | | | | | | | | | | | | | | | | | | | | | | | | | | | | | | | | | | | | | | | | | | | | | | | | | | | | | | | | | | | | | | | | | | | | | | | | | | | | | | | | | | | | | | | | | | | | | | | | | | | | | | | | | | | | | | | | | | | | | | | | | | | | | | | | | | | | | | | | | | | | | | | | | | | | | | | | | | | | | | | | | | | | |  |
| **Authors:** Mendis, P., Kachingwe, A. & Khabele, M.I. | | | | | | | | | | | | | | | | | | | | | | | | | | | | | | | | | | | | | | | | | | | | | | | | | | | | | | | | | | | | | | | | | | | | | | | | | | | | | | | | | | | | | | | | | | | | | | | | | | | | | | | | | | | | | | | | | | | | | | | | | | | | | | | | | | | | | | | | | | | | | | | | | | | | | | | | | | | | | | | | | | | | | | | | | | | | | | | | | | | | | | | | | | | | | | | | | | | | | | | | | | | | | | | | | | | | | | **Year:** 2009 | | | | | | | | | | | | | | | | | | | | | | | | | | | | | | | | | | | | | | | | | | | | | | | | | | | | | | | | | |  |
| **Summary:** Evaluation to provide recommendations to strengthen CBR programme run by voluntary lay health workers. | | | | | | | | | | | | | | | | | | | | | | | | | | | | | | | | | | | | | | | | | | | | | | | | | | | | | | | | | | | | | | | | | | | | | | | | | | | | | | | | | | | | | | | | | | | | | | | | | | | | | | | | | | | | | | | | | | | | | | | | | | | | | | | | | | | | | | | | | | | | | | | | | | | | | | | | | | | | | | | | | | | | | | | | | | | | | | | | | | | | | | | | | | | | | | | | | | | | | | | | | | | | | | | | | | | | | | | | | | | | | | | | | | | | | | | | | | | | | | | | | | | | | | | | | | | | | | | | | | | | | | | | | | | | | | | |  |
| **Setting** | | | | | | | | **Design/**  **Methods** | | | | | | | | | | | | | | | | | | | | | | | | | | | | | | | | **Population** | | | | | | | | | | | | | | | | | | | | | | | | | | | | | | | | | | | | **Intervention** | | | | | | | | | | | | | | | | | | | | | | | | | | | | | | **Workforce Characteristics** | | | | | | | | | | | | | | | | | | | | | | | | | | | | | | | | | | | | | | | | | | | | | | | | | | | | | | | | | | | | | | | | | | | | | | | | | | | | | | | | | | | | | | | | | | | | | | | | | | | | | | | | | | | | | | | | | | | | | | | | | | | | | | | | | | | | | | | | | | | | | | | | | | | | | | | | | | | | | | | | | | | | | |  |
|  |  |  |  |  |  |  |  |  |  |  |  |  |  |  |  |  |  |  |  |  |  |  |  |  |  |  |  |  |  |  |  |  |  |  |  |  |  |  |  |  |  |  |  |  |  |  |  |  |  |  |  |  |  |  |  |  |  |  |  |  |  |  |  |  |  |  |  |  |  |  |  |  |  |  |  |  |  |  |  |  |  |  |  |  |  |  |  |  |  |  |  |  |  |  |  |  |  |  |  |  |  |  |  |  |  | **Cadres** | | | | | | | | | | | | | | | | | | | | | | | | | | | | | | | | | | | | **Description** | | | | | | | | | | | | | | | | | | | | | | | | | | | | | | | | | | | | | | | | | | | | | | **Training** | | | | | | | | | | | | | | | | | | | | | | | | | | **Supervision** | | | | | | | | | | | | | | | | | | | | | | | | | | | | | | | | | | | | | | | | | | | | | | | **Misc.** | | | | | | | | | | |  |
| Lesotho | | | | | | | | Evaluation Report using intensive interviews and meetings with stakeholders. | | | | | | | | | | | | | | | | | | | | | | | | | | | | | | | | Persons with physical disabilities | | | | | | | | | | | | | | | | | | | | | | | | | | | | | | | | | | | | Home visits by local supervisors to persons with disabilities. | | | | | | | | | | | | | | | | | | | | | | | | | | | | | | Local Supervisors (LS) | | | | | | | | | | | | | | | | | | | | | | | | | | | | | | | | | | | | Voluntary, though health workers now paid stipend of M300. 37/45 LS are women. | | | | | | | | | | | | | | | | | | | | | | | | | | | | | | | | | | | | | | | | | | | | | |  | | | | | | | | | | | | | | | | | | | | | | | | | | Supervision from District Rehabilitation Officers | | | | | | | | | | | | | | | | | | | | | | | | | | | | | | | | | | | | | | | | | | | | | | | **Quality: 3**  MMAT: Qualitative | | | | | | | | | | |  |
| **CMOCs** | | | | | | | | | | | | | | | | | | | | | | | | | | | | | | | | | | | | | | | | | | | | | | | | | | | | | | | | | | | | | | | | | | | | | | | | | | | | | | | | | | | | | | | | | | | | | | | | | | | | | | | | | | | | | | | | | | | | | | | | | | | | | | | | | | | | | | | | | | | | | | | | | | | | | | | | | | | | | | | | | | | | | | | | | | | | | | | | | | | | | | | | | | | | | | | | | | | | | | | | | | | | | | | | | | | | | | | | | | | | | | | | | | | | | | | | | | | | | | | | | | | | | | | | | | | | | | | | | | | | | | | | | | | | | | | |  |
| **Context** | | | | | | | | | | | | | | | | | | | | | | | | | | | | | | | | | | | | | | | | | | | | | | | | | | | | | | | **Mechanisms** | | | | | | | | | | | | | | | | | | | | | | | | | | | | | | | | | | | | | | | | | | | **Outcomes** | | | | | | | | | | | | | | | | | | | | | | | | | | | | | | | | | | | | | | | | | | | | | | | | | | | | | | | | | | | | | | | | | | | | | | | | | | | | | | | | | | | | | | | | | | | | | | | | | | | | | | | **CMOCs** | | | | | | | | | | | | | | | | | | | | | | | | | | | | | | | | | | | | | | | | | | | | | | | | | | | | | | | | | | | | | | | | | | | | | | |  |
| Government of Lesotho and Norwegian Association of the Disabled have worked together to develop CBR programme since 2003.  Local supervisors work mostly in remote areas and individually.  Monthly visits to households with persons with disabilities by Local Supervisors.  Majority of disabilities are mobility impairments or visual impairments.  Persons with mental impairments are typically referred to health centres by local supervisors.  Persons with disabilities consulted on issues most pertinent to them for purposes of programme.  Expensive health facility visits often barrier for persons to seek care. | | | | | | | | | | | | | | | | | | | | | | | | | | | | | | | | | | | | | | | | | | | | | | | | | | | | | | | 1.Community based workers not trained in in empowerment skills.  2. Community based workers not training on advocacy and empowerment skills lack ability to mobilize communities  3. Lack of communications, and coordinated, clear job descriptions can result in inefficiency | | | | | | | | | | | | | | | | | | | | | | | | | | | | | | | | | | | | | | | | | | | Travel costs and other barriers often impeded or inhibit referrals.  Visits from District Rehabilitation Officers are infrequent, leaving Local Supervisors to work independently with little supervision.  Local Supervisors have taken initiative to meet in informal groups when possible to support each other.  Local supervisors have low drop out rate (5/50 in approx. 4 years).  Livelihoods recognized as being in urgent need of support due to depth of poverty.  Ministry of Health considering having CHWs train as LS, however questions regarding workload are raised.  Very limited community engagement, ownership and participation,  Little coordination between organizations resulting in workforce duplication.  Lack of community empowerment.  Lack of monitoring skills of health workforce.  Local supervisors face multiple barriers in providing services including difficulty with terrain and lack of support from supervisors. | | | | | | | | | | | | | | | | | | | | | | | | | | | | | | | | | | | | | | | | | | | | | | | | | | | | | | | | | | | | | | | | | | | | | | | | | | | | | | | | | | | | | | | | | | | | | | | | | | | | | | | 1. Community based rehabilitation offered by workers who come to communities especially important for marginalized and vulnerable populations to decrease barriers to accessing services.  2.Community based works should be trained on Rights Based Approach and have skills in mobilization, advocacy and empowerment  3. Rehabilitation workers require training on monitoring and ability to conduct group activities  4. Clear job descriptions including roles and responsibilities of different cadres should be established and communicated throughout health system and organizations working in rehabilitation  5. Establishment of Steering Committees or focal person to coordinate rehabilitation activities. | | | | | | | | | | | | | | | | | | | | | | | | | | | | | | | | | | | | | | | | | | | | | | | | | | | | | | | | | | | | | | | | | | | | | | |  |
| **Title:** Outcomes of the mental health and development model in rural Kenya: a 2-year prospective cohort intervention study | | | | | | | | | | | | | | | | | | | | | | | | | | | | | | | | | | | | | | | | | | | | | | | | | | | | | | | | | | | | | | | | | | | | | | | | | | | | | | | | | | | | | | | | | | | | | | | | | | | | | | | | | | | | | | | | | | | | | | | | | | | | | | | | | | | | | | | | | | | | | | | | | | | | | | | | | | | | | | | | | | | | | | | | | | | | | | | | | | | | | | | | | | | | | | | | | | | | | | | | | | | | | | | | | | | | | | | | | | | | | | | | | | | | | | | | | | | | | | | | | | | | | | | | | | | | | | | | | | | | | | | | | | | | | | | |  |
| **Authors:** Lund, C., Waruguru, M., Kingori, J., Kippen-Wood, S., Breuer, E., Mannarath, S. & Raja, S. | | | | | | | | | | | | | | | | | | | | | | | | | | | | | | | | | | | | | | | | | | | | | | | | | | | | | | | | | | | | | | | | | | | | | | | | | | | | | | | | | | | | | | | | | | | | | | | | | | | | | | | | | | | | | | | | | | | | | | | | | | | | | | | | | | | | | | | | | | | | | | | | | | | | | | | | | | | | | | | | | | | | | | | | | | | | | | | | | | | | | | | | | | | | | | | | | | | | | | | | | | | | | | | | | | | | | | | | | **Year:** 2013 | | | | | | | | | | | | | | | | | | | | | | | | | | | | | | | | | | | | | | | | | | | | | | | | | | | | | | |  |
| **Summary:** Reports on a mental health and development (MHD) programme run by BasicNeeds in rural Kenya using lay health workers in an multifaceted tiered delivery system | | | | | | | | | | | | | | | | | | | | | | | | | | | | | | | | | | | | | | | | | | | | | | | | | | | | | | | | | | | | | | | | | | | | | | | | | | | | | | | | | | | | | | | | | | | | | | | | | | | | | | | | | | | | | | | | | | | | | | | | | | | | | | | | | | | | | | | | | | | | | | | | | | | | | | | | | | | | | | | | | | | | | | | | | | | | | | | | | | | | | | | | | | | | | | | | | | | | | | | | | | | | | | | | | | | | | | | | | | | | | | | | | | | | | | | | | | | | | | | | | | | | | | | | | | | | | | | | | | | | | | | | | | | | | | | |  |
| **Setting** | | | | | | | | **Design/**  **Methods** | | | | | | | | | | | | | | | | | | | **Population** | | | | | | | | | | | | | | | | | | | | | | | | | | | | | | | | | | | | | | | | | | | | | | **Intervention** | | | | | | | | | | | | | | | | | | | | | | | | | | | | | | | | | | | | | **Workforce Characteristics** | | | | | | | | | | | | | | | | | | | | | | | | | | | | | | | | | | | | | | | | | | | | | | | | | | | | | | | | | | | | | | | | | | | | | | | | | | | | | | | | | | | | | | | | | | | | | | | | | | | | | | | | | | | | | | | | | | | | | | | | | | | | | | | | | | | | | | | | | | | | | | | | | | | | | | | | | | | | | | | | | |  |
|  |  |  |  |  |  |  |  |  |  |  |  |  |  |  |  |  |  |  |  |  |  |  |  |  |  |  |  |  |  |  |  |  |  |  |  |  |  |  |  |  |  |  |  |  |  |  |  |  |  |  |  |  |  |  |  |  |  |  |  |  |  |  |  |  |  |  |  |  |  |  |  |  |  |  |  |  |  |  |  |  |  |  |  |  |  |  |  |  |  |  |  |  |  |  |  |  |  |  |  |  |  |  |  |  |  |  |  |  |  | **Cadres** | | | | | | | | | | | | | | | | | | | | | | | | | | | | | | | | | | **Description** | | | | | | | | | | | | | | | | | | | | | | | | | | | | | | | | | | | | | | | | | | | | | | | | | | | | **Training** | | | | | | | | | | | | | | | | | | | | | | | | | | | | | | | | | | | | | | | | | | | | | | | | | | | | **Supervision** | | | | | | | | | | | | | | | | | | **Misc.** | | | | | |  |
| Rural Meru South and Nyeri North districts, Kenya | | | | | | | | Single group cohort, with quantitative analysis. | | | | | | | | | | | | | | | | | | | Persons with severe mental or neurological disorder enrolled in BasicNeeds’ Mental Health and Development Programme (n=203) | | | | | | | | | | | | | | | | | | | | | | | | | | | | | | | | | | | | | | | | | | | | | | Mental Health and Development Programme – community engagement and mobilization, with diagnosis and treatment prescription by psychiatric nurse and follow-up by CBWs. CBWs lead group support groups. | | | | | | | | | | | | | | | | | | | | | | | | | | | | | | | | | | | | | Community based workers (CBWs), Psychiatric Nurse | | | | | | | | | | | | | | | | | | | | | | | | | | | | | | | | | | CBWs are lay workers that operate under Kenyan MoH, must be literate, from community, and nominated by community. Role in programme to identify, refer, medication management, facilitate user and carer self-help groups (with 15-25 people).  Nurses diagnose and prescribe medicine. | | | | | | | | | | | | | | | | | | | | | | | | | | | | | | | | | | | | | | | | | | | | | | | | | | | | 5 days minimum, covering introduction to mental illness and counseling skills. Must have attended training, had practiced 3 sessions of group facilitation and mastered the 10 steps of self-help groups before working. | | | | | | | | | | | | | | | | | | | | | | | | | | | | | | | | | | | | | | | | | | | | | | | | | | | | Not discussed | | | | | | | | | | | | | | | | | | Serve 20 HH or 500 persons.  **Quality: 3**  MMAT Quant 4 | | | | | |  |
| **CMOCs** | | | | | | | | | | | | | | | | | | | | | | | | | | | | | | | | | | | | | | | | | | | | | | | | | | | | | | | | | | | | | | | | | | | | | | | | | | | | | | | | | | | | | | | | | | | | | | | | | | | | | | | | | | | | | | | | | | | | | | | | | | | | | | | | | | | | | | | | | | | | | | | | | | | | | | | | | | | | | | | | | | | | | | | | | | | | | | | | | | | | | | | | | | | | | | | | | | | | | | | | | | | | | | | | | | | | | | | | | | | | | | | | | | | | | | | | | | | | | | | | | | | | | | | | | | | | | | | | | | | | | | | | | | | | | | | |  |
| **Context** | | | | | | | | | | | | | | | | | | | | | | | | | | | | | | | | | | | | | | | | | | | | | | | | | | | | | | | | | | | | | | | | | | | | | | | | | | | | | | | | | | | | | | | | | | | **Mechanisms** | | | | | | | | | | | | | | | | | | | | | | | | | | | | | | | | | | | | | | | | | | | | | | | | | | | | | | | | | | | | | | | | | | **Outcomes** | | | | | | | | | | | | | | | | | | | | | | | | | | | | | | | | | | | | | | | | | | | | | | | | | | | | | | | | | | | | | | **CMOCs** | | | | | | | | | | | | | | | | | | | | | | | | | | | | | | | | | | | | | | | | | | | | | | | | | | | | |  |
| BasicNeeds established NGO, running 16 programmes in 10 countries since 2000. MHD programme has been used in different contexts, and has 5 interlinked modules: capacity building, community mental health, sustainable livelihoods, research and management/administration. Rural Meru South and Nyeri North districts, with limited health coverage. One psychiatric nurse in each district and no public psychiatrists or psychologists. Some primary health care staff received mental health training but their services in primary care are in high demand. Intervention began with community meetings in collaboration with other local NGOs. Individuals with mental illness or family members invited to identify themselves to enroll in programme, and also created awareness and spread information on the self-help groups. Individuals with mental illness go to psychiatric nurse at health centre who diagnoses and treats. Trained and employed by the MoH. Community based health workers (CBWs) that are a formal part of health system and criteria set by MoH given opportunity to volunteer with BasicNeeds and undergo further training. Role is to work with community with purpose of assisting health professionals in MoH clinics. In MHD programme, role: identification of symptoms of mental disorders; refer to local primary care psychiatric clinics for assessment and medication management; facilitate support groups. Role advertised in local health facilities. Then underwent training: concepts of mental illness, myths, signs and symptoms, common conditions, referrals, basic counseling, recording, motivating groups. | | | | | | | | | | | | | | | | | | | | | | | | | | | | | | | | | | | | | | | | | | | | | | | | | | | | | | | | | | | | | | | | | | | | | | | | | | | | | | | | | | | | | | | | | | | 1.Reputation of NGO may have increased individuals’ willingness to identify themselves and participant in programme  2. Reputation of CBWs, as already identified and from community, may have increased individuals willingness to participant  3. Involving families/carers in programmes may have increased acceptance by individuals  4. Tier system for delivery of services can assist in ensuring programme followed through by participants, with monitoring checks in communities  5. Programmes and the workforce they employ should work within the existing workforce and MoH priorities  6. CBHWs can have strong influence on referrals, medication use, follow-ups, increasing group meets and counseling sessions, and diagnoses. | | | | | | | | | | | | | | | | | | | | | | | | | | | | | | | | | | | | | | | | | | | | | | | | | | | | | | | | | | | | | | | | | | 203 participants enrolled at baseline, and at 24 month follow-up 174 (attrition rate of 14.3%, with majority of loss due to moving (10), deceased (8), cannot locate (8) and only 2 declined interview and 1 defaulted on treatment.  Significant improvements in general health (GHQ-12); WHOQOL quality of life; proportion engaged in income or productive work; Global Assessment of Functioning (GAF) scores; median monthly family income.  Significant reduction in: proportion of participants reporting receiving help from carers at home; and proportion of participants reporting that carers in home left their jobs to care for them.  Referrals from baseline-endline rose 15.8% - 45.1%; meds received rom 65.2% to 99.4%; follow-up form 6.4% - 88.4%; group meetings from 21.9% - 97.1%; self help groups from 6.9% - 95.4%; counseling from 72.9% - 100%; diagnosis from 89.2% - 100%. | | | | | | | | | | | | | | | | | | | | | | | | | | | | | | | | | | | | | | | | | | | | | | | | | | | | | | | | | | | | | | 1.Further train already working lay health workers  2.Lay health workers should be integrated into formal health system and supported by NGOs  3. Carers should be involved in programmes for persons with mental illness, with specific support structures for them  4.Psychiatric nurses should diagnose and prescribe medication  5.CBWs should identify, refer and monitor medication management in communities.  6. CBWs should be trained on mobilization of communities  7.CBWs should be trained specifically on group therapy/management  8.Tier system of service delivery with general skills in community to more specialized skills in health centre  9. CBWs are effective in providing support for persons with mental illness in communities  10. Programmes and their workforce should be integrated into existing MoH initiatives | | | | | | | | | | | | | | | | | | | | | | | | | | | | | | | | | | | | | | | | | | | | | | | | | | | | |  |
| **Title: Vietnam CBR Project – Opinions of Project Beneficiaries** | | | | | | | | | | | | | | | | | | | | | | | | | | | | | | | | | | | | | | | | | | | | | | | | | | | | | | | | | | | | | | | | | | | | | | | | | | | | | | | | | | | | | | | | | | | | | | | | | | | | | | | | | | | | | | | | | | | | | | | | | | | | | | | | | | | | | | | | | | | | | | | | | | | | | | | | | | | | | | | | | | | | | | | | | | | | | | | | | | | | | | | | | | | | | | | | | | | | | | | | | | | | | | | | | | | | | | | | | | | | | | | | | | | | | | | | | | | | | | | | | | | | | | | | | | | | | | | | | | | | | | | | | | | | | | | |  |
| **Authors: Deepak, S.** | | | | | | | | | | | | | | | | | | | | | | | | | | | | | | | | | | | | | | | | | | | | | | | | | | | | | | | | | | | | | | | | | | | | | | | | | | | | | | | | | | | | | | | | | | | | | | | | | | | | | | | | | | | | | | | | | | | | | | | | | | | | | | | | | | | | | | | | | | | | | | | | | | | | | | | | | | | | | | | | | | | | | | | | | | | | | | | | | | | | | | | | | | | | | | | | | | | | | | | | | | | | | | | | | | | | | | | | | **Year: 2010** | | | | | | | | | | | | | | | | | | | | | | | | | | | | | | | | | | | | | | | | | | | | | | | | | | | | | | |  |
| **Summary:** Midterm evaluation including assessment of CBR volunteer and supervisions perceptions of training, programme and impact of CBR programme | | | | | | | | | | | | | | | | | | | | | | | | | | | | | | | | | | | | | | | | | | | | | | | | | | | | | | | | | | | | | | | | | | | | | | | | | | | | | | | | | | | | | | | | | | | | | | | | | | | | | | | | | | | | | | | | | | | | | | | | | | | | | | | | | | | | | | | | | | | | | | | | | | | | | | | | | | | | | | | | | | | | | | | | | | | | | | | | | | | | | | | | | | | | | | | | | | | | | | | | | | | | | | | | | | | | | | | | | | | | | | | | | | | | | | | | | | | | | | | | | | | | | | | | | | | | | | | | | | | | | | | | | | | | | | | |  |
| **Setting** | | | | | | | | | | | | | | | | **Design/**  **Methods** | | | | | | | | | | | | | | | | | | | | | | | | | | | | | | **Population** | | | | | | | | | | | | | | | | | | | | | | | | | | | | | | | **Intervention** | | | | | | | | | | | | | | | | | | | | | | | | | | | | | | **Workforce Characteristics** | | | | | | | | | | | | | | | | | | | | | | | | | | | | | | | | | | | | | | | | | | | | | | | | | | | | | | | | | | | | | | | | | | | | | | | | | | | | | | | | | | | | | | | | | | | | | | | | | | | | | | | | | | | | | | | | | | | | | | | | | | | | | | | | | | | | | | | | | | | | | | | | | | | | | | | | | | | | | | | | | | | | |  |
|  |  |  |  |  |  |  |  |  |  |  |  |  |  |  |  |  |  |  |  |  |  |  |  |  |  |  |  |  |  |  |  |  |  |  |  |  |  |  |  |  |  |  |  |  |  |  |  |  |  |  |  |  |  |  |  |  |  |  |  |  |  |  |  |  |  |  |  |  |  |  |  |  |  |  |  |  |  |  |  |  |  |  |  |  |  |  |  |  |  |  |  |  |  |  |  |  |  |  |  |  |  |  |  |  |  |  | **Cadres** | | | | | | | | | | | | | | | | | | | | | | | | | | | | | | | | | | | | | **Description** | | | | | | | | | | | | | | | | | | | | | | | | | | | | | | | | **Training** | | | | | | | | | | | | | | | | | | | | | | | | | | | | | | | | | | | | | | | | | | | | | **Supervision** | | | | | | | | | | | | | | | | | | | | | | | | | | | | | | **Misc.** | | | | | | | | | | | | | | | | | | | | |  |
| Hai Phong, Phy Tho provinces in North, and Thua Thien Hue, Nghe An, Da Nang and Binh Dinh provinces in central, Vietnam | | | | | | | | | | | | | | | | Mid-term evaluation – questionnaires | | | | | | | | | | | | | | | | | | | | | | | | | | | | | | Persons with disabilities | | | | | | | | | | | | | | | | | | | | | | | | | | | | | | | Follows CBR Matrix, mainstreamed in education and vocational activities involving networks with different governmental and NGO organizations. | | | | | | | | | | | | | | | | | | | | | | | | | | | | | | CBR Volunteers; primary school teachers, self help groups, | | | | | | | | | | | | | | | | | | | | | | | | | | | | | | | | | | | | | Identified by community, and trained through the CBR programme. No income or in-kind compensation for their work. 2,340 CBR volunteers trained from Jan 2008-Dec 2009. | | | | | | | | | | | | | | | | | | | | | | | | | | | | | | | | For CBR supervisors, CBR volunteers in community level. Also training on other development activities (livelihoods, education etc.). | | | | | | | | | | | | | | | | | | | | | | | | | | | | | | | | | | | | | | | | | | | | | Rehabilitation Department of University of Hanoi. Supervisors are volunteers, | | | | | | | | | | | | | | | | | | | | | | | | | | | | | | Cover around 3-4 villages, and 20 persons with disability.  **Quality – 2**  MMAT - qualitative | | | | | | | | | | | | | | | | | | | | |  |
| **CMOCs** | | | | | | | | | | | | | | | | | | | | | | | | | | | | | | | | | | | | | | | | | | | | | | | | | | | | | | | | | | | | | | | | | | | | | | | | | | | | | | | | | | | | | | | | | | | | | | | | | | | | | | | | | | | | | | | | | | | | | | | | | | | | | | | | | | | | | | | | | | | | | | | | | | | | | | | | | | | | | | | | | | | | | | | | | | | | | | | | | | | | | | | | | | | | | | | | | | | | | | | | | | | | | | | | | | | | | | | | | | | | | | | | | | | | | | | | | | | | | | | | | | | | | | | | | | | | | | | | | | | | | | | | | | | | | | | |  |
| **Context** | | | | | | | | | | | | | | | | | | | | | | | | | | | | | | | | | | | | | | | | | | | | | | | | | | | | | | | | | | | | **Mechanisms** | | | | | | | | | | | | | | | | | | | | | | | | | | | | | | | | | | | | | | | | | | | | | | | | | | | | | | | | | | | | | | | | | | | | **Outcomes** | | | | | | | | | | | | | | | | | | | | | | | | | | | | | | | | | | | | | | | | | | | | | | | | | | | | | | | | | | | | | | | | | | | | | | | | | **CMOCs** | | | | | | | | | | | | | | | | | | | | | | | | | | | | | | | | | | | | | | | | | | | | | | | | | | | | | | | | | | | | | | | | | | | | | | |  |
| Programme management is based in Hanoi, but the activities of individual provinces in partnership with differing institutions within that province. All projects co-funded by Italian Development Cooperation of Italian Foreign Ministry and coordinated by AIFO.  Conception of programme in 2001, with first year 2008/09 and by 2010 covered 234 communities in 18 districts  CBR volunteers are frontline but also involved are primary school teachers, vocational training centres, self-help groups etc.  Questionnaires with CBR volunteers and CBR supervisors on training and work.  Training courses for district level supervisors for training 200 persons; 47 training courses for CBR volunteers at community level, training 1410 persons. | | | | | | | | | | | | | | | | | | | | | | | | | | | | | | | | | | | | | | | | | | | | | | | | | | | | | | | | | | | | 1. Proposed mechanism for intra-programme, inter-project organizational learning is that groups meet to discuss differences in their mean scores in perception.  2. Individuals selected from and by their communities accepting of volunteering roles  3. Community programmes that integrate CBR Matrix activities, and focus on livelihoods, can have positive impact on PWD | | | | | | | | | | | | | | | | | | | | | | | | | | | | | | | | | | | | | | | | | | | | | | | | | | | | | | | | | | | | | | | | | | | | Satisfaction with their roles was high amongst volunteers.  Volunteers report high quality of CBR training.  Volunteers report that communities have high level of consideration for PWD.  Both workers and supervisors are positive about training and community appreciation of their work.  80 to 90% of schoolteachers confirm that they received information about disability issues.  Among male volunteers, (32% of sample of volunteers, modal category was high school education 46.5% had it to this level. Among female volunteers, high school education was again the modal category (50%). | | | | | | | | | | | | | | | | | | | | | | | | | | | | | | | | | | | | | | | | | | | | | | | | | | | | | | | | | | | | | | | | | | | | | | | | | 1.Inter-organizational and intra-organizational communication about differences in perceived levels of service.  2. Workforce volunteers should be selected from and by the communities in which they serve  3. Working within the CBR Matrix is essential for rehabilitation in the community  4. Integration and cooperation of varying organizations for the rehabilitation of PWD, under the guidance of the CBR Matrix | | | | | | | | | | | | | | | | | | | | | | | | | | | | | | | | | | | | | | | | | | | | | | | | | | | | | | | | | | | | | | | | | | | | | | |  |
| **Title: Pacific Rehabilitation Health Workforce: WHO Discussion Paper Series, Paper No. 1** | | | | | | | | | | | | | | | | | | | | | | | | | | | | | | | | | | | | | | | | | | | | | | | | | | | | | | | | | | | | | | | | | | | | | | | | | | | | | | | | | | | | | | | | | | | | | | | | | | | | | | | | | | | | | | | | | | | | | | | | | | | | | | | | | | | | | | | | | | | | | | | | | | | | | | | | | | | | | | | | | | | | | | | | | | | | | | | | | | | | | | | | | | | | | | | | | | | | | | | | | | | | | | | | | | | | | | | | | | | | | | | | | | | | | | | | | | | | | | | | | | | | | | | | | | | | | | | | | | | | | | | | | | | | | | | |  |
| **Authors:** Llewellyn, G., Gargett, A. & Short, S. | | | | | | | | | | | | | | | | | | | | | | | | | | | | | | | | | | | | | | | | | | | | | | | | | | | | | | | | | | | | | | | | | | | | | | | | | | | | | | | | | | | | | | | | | | | | | | | | | | | | | | | | | | | | | | | | | | | | | | | | | | | | | | | | | | | | | | | | | | | | | | | | | | | | | | | | | | | | | | | | | | | | | | | | | | | | | | | | | | | | | | | | | | | | | | | | | | | | | | | | | | | | | | | | | | | | | | | | | **Year:** 2012 | | | | | | | | | | | | | | | | | | | | | | | | | | | | | | | | | | | | | | | | | | | | | | | | | | | | | | |  |
| **Summary:** Report on the Pacific Island countries rehabilitation workforce with evidence from literature and stakeholders | | | | | | | | | | | | | | | | | | | | | | | | | | | | | | | | | | | | | | | | | | | | | | | | | | | | | | | | | | | | | | | | | | | | | | | | | | | | | | | | | | | | | | | | | | | | | | | | | | | | | | | | | | | | | | | | | | | | | | | | | | | | | | | | | | | | | | | | | | | | | | | | | | | | | | | | | | | | | | | | | | | | | | | | | | | | | | | | | | | | | | | | | | | | | | | | | | | | | | | | | | | | | | | | | | | | | | | | | | | | | | | | | | | | | | | | | | | | | | | | | | | | | | | | | | | | | | | | | | | | | | | | | | | | | | | |  |
| **Setting** | | | | | | | | **Design/Methods** | | | | | | | | | | | | | | | | | | | | | | | | | | | | | | | | | **Population** | | | | | | | | | | | | | | | | | | | | | | | | | | | | | | | | | | | | **Intervention** | | | | | | | | | | | | | | | | | | | | | | | | | | | | | | **Workforce Characteristics** | | | | | | | | | | | | | | | | | | | | | | | | | | | | | | | | | | | | | | | | | | | | | | | | | | | | | | | | | | | | | | | | | | | | | | | | | | | | | | | | | | | | | | | | | | | | | | | | | | | | | | | | | | | | | | | | | | | | | | | | | | | | | | | | | | | | | | | | | | | | | | | | | | | | | | | | | | | | | | | | | | | | |  |
|  |  |  |  |  |  |  |  |  |  |  |  |  |  |  |  |  |  |  |  |  |  |  |  |  |  |  |  |  |  |  |  |  |  |  |  |  |  |  |  |  |  |  |  |  |  |  |  |  |  |  |  |  |  |  |  |  |  |  |  |  |  |  |  |  |  |  |  |  |  |  |  |  |  |  |  |  |  |  |  |  |  |  |  |  |  |  |  |  |  |  |  |  |  |  |  |  |  |  |  |  |  |  |  |  |  |  | **Cadres** | | | | | | | | | | | | | | | | | | | | | | | | | | | | | | | | | | | | | | | | | | | | | | | | **Description** | | | | | | | | | | | | | | | | | | | | | **Training** | | | | | | | | | | | | | | | | | | | | | | | | | | | | | | | | | | | | | | | | | **Supervision** | | | | | | | | | | | | | | | | | | | | | | | | | | | | | | | | | | **Misc.** | | | | | | | | | | | | | | | | | | | | |  |
| Pacific Island Countries | | | | | | | | Desk review, key informant interviews, workshop, and priorities ranking | | | | | | | | | | | | | | | | | | | | | | | | | | | | | | | | | Rehabilitation workforce in Pacific Island countries | | | | | | | | | | | | | | | | | | | | | | | | | | | | | | | | | | | | Not discussed though varied between countries | | | | | | | | | | | | | | | | | | | | | | | | | | | | | | 38 services in rehabilitation identified (p. 4). Most often physiotherapy (31%) down to speech therapy (5%). | | | | | | | | | | | | | | | | | | | | | | | | | | | | | | | | | | | | | | | | | | | | | | | | Majority of services concentrated at the national level (60%) and only 2% were reported at the community/household level. | | | | | | | | | | | | | | | | | | | | | Country specific.  Large variety with a many reports of Tertiary training in several countries; | | | | | | | | | | | | | | | | | | | | | | | | | | | | | | | | | | | | | | | | | Country specific | | | | | | | | | | | | | | | | | | | | | | | | | | | | | | | | | | **Quality: NA** Descriptive report | | | | | | | | | | | | | | | | | | | | |  |
| **CMOCs** | | | | | | | | | | | | | | | | | | | | | | | | | | | | | | | | | | | | | | | | | | | | | | | | | | | | | | | | | | | | | | | | | | | | | | | | | | | | | | | | | | | | | | | | | | | | | | | | | | | | | | | | | | | | | | | | | | | | | | | | | | | | | | | | | | | | | | | | | | | | | | | | | | | | | | | | | | | | | | | | | | | | | | | | | | | | | | | | | | | | | | | | | | | | | | | | | | | | | | | | | | | | | | | | | | | | | | | | | | | | | | | | | | | | | | | | | | | | | | | | | | | | | | | | | | | | | | | | | | | | | | | | | | | | | | | |  |
| **Context** | | | | | | | | | | | | | | | | | | | | | | | | | | | | | | | | | | | | | | | | | **Mechanisms** | | | | | | | | | | | | | | | | | | | | | | | | | | | | | | | | | | | | | | | | | | | | | | | | | | | **Outcomes** | | | | | | | | | | | | | | | | | | | | | | | | | | | | | | | | | | | | | | | | | | | | | | | | | | | | | | | | | | | | | | | | | | | | | | | | | | | | | | | | | | | | | | | | | | | | | | | | | | | | | | | | | | | | | | | | | | | | | | | | | | | | | | | | | **CMOCs** | | | | | | | | | | | | | | | | | | | | | | | | | | | | | | | | | | | | | | | | | | | | | | | | | | |  |
| Varied depending on country and context.  Lack of available rehabilitation workforce information and data kept at country level.  Most literature focused on specific approaches.  More than one third of the reports were focused on two countries, Fiji and Papua New Guinea.  Most research papers and reports (as is the case more widely) don’t focus on the workforce; they focus on the interventions and services.  Dispersed and often small populations.  Costly travel, varying language and cultural groups with differing views of disabilities and appropriate services.  Country reports primarily address mortality and/or prevention, with little information being provided on morbidity and disability. | | | | | | | | | | | | | | | | | | | | | | | | | | | | | | | | | | | | | | | | | 1.Lack of information on the rehabilitation workforce, especially from less specialized cadres that were not trained in tertiary institutions, may bias estimates (numbers, cadres type etc.) of individuals working on rehabilitation.  2.The majority of reports or publications focus on programmes and lack details on the workforce that are implementing such interventions  3. Lack of information from health officials and health workers on the benefits or scope of rehabilitation work could be a limiting factor due to lack of prioritization or commitment to such services | | | | | | | | | | | | | | | | | | | | | | | | | | | | | | | | | | | | | | | | | | | | | | | | | | | Majority of services concentrated at the national level (60%) and only 2% were reported at the community/household level.  50 rehabilitation personnel reported, mostly physiotherapists and 38 services in rehabilitation identified - most often physiotherapy (31%) down to speech therapy (5%).  Much of the reported health workforce was an international volunteer on short-term placements.  Most services focused at national level, not at the level of households.  Lack of reliable data on disability and needs for rehabilitation health services.  Lack of health workforce data “at the level of granularity to determine rehabilitation health workers”.  Lack of awareness of what rehabilitation services could potentially offer.  Need for culturally relevant training delivered by host country nationals who will stay, and connect with their people as clients.  Need for integrated services, from primary care to a tertiary institutional base for more complex referrals.  Educating cadres internationally not likely in the foreseeable future, therefore require a localized, problem-focused mechanism.  Regional approaches might afford some economy of scale and shared models based on shared assumptions, values etc.  Only 4 countries reported using clinical guidelines, most services available only at national level.  Teamwork and collaboration was reported to be lacking.  Recognition that community based rehabilitation services are needed – including appropriate referrals, collaborative relationships and community involvement. | | | | | | | | | | | | | | | | | | | | | | | | | | | | | | | | | | | | | | | | | | | | | | | | | | | | | | | | | | | | | | | | | | | | | | | | | | | | | | | | | | | | | | | | | | | | | | | | | | | | | | | | | | | | | | | | | | | | | | | | | | | | | | | | | 1. Need for an integrated rehabilitation model that links general (community) service to tertiary (specialized) services  2. Rehabilitation workforce requires specific a cadre to support locally assessable and acceptable interventions  3. International collaboration of training for specialized skills with locally relevant techniques and examples  4. Stronger record keeping and data management of the workforce itself  5. Dissemination activities of rehabilitation programmes should include more details on the workforce who implement such programmes  6. Bringing rehabilitation services to the community is an effective technique to reach individuals who may have barriers to seeking more specialized or tertiary care  7. Education on benefits of rehabilitation, especially for decisions makers, is important to increase its prioritization within the health sector | | | | | | | | | | | | | | | | | | | | | | | | | | | | | | | | | | | | | | | | | | | | | | | | | | |  |
